# Integrative multi-omics identifies AP-1 transcription factor as a targetable mediator of acquired osimertinib resistance in non-small cell lung cancer

**DOI:** 10.1038/s41419-025-07711-z

**Published:** 2025-05-25

**Authors:** Bengisu Dayanc, Sude Eris, Nazife Ege Gulfirat, Gulden Ozden-Yilmaz, Ece Cakiroglu, Ozlem Silan Coskun Deniz, Gökhan Karakülah, Serap Erkek-Ozhan, Serif Senturk

**Affiliations:** 1 Izmir Biomedicine and Genome Center, Izmir, Türkiye; 2 Izmir International Biomedicine and Genome Institute, Dokuz Eylul University, Izmir, Türkiye

**Keywords:** Cancer genomics, Gene silencing, Non-small-cell lung cancer

## Abstract

Osimertinib, a third-generation EGFR tyrosine kinase inhibitor (EGFR-TKI), has dramatically transformed the treatment landscape for patients with *EGFR*-mutant NSCLC. However, the long-term success of this therapy is often compromised by the onset of acquired resistance, with non-genetic mechanisms increasingly recognized as pivotal contributors. Here, we exploit a multi-omics approach to profile genome-wide chromatin accessibility and transcriptional landscapes between drug sensitive and resistant *EGFR*-mutant cells. Our findings reveal a robust concordance between epigenetic regulome and transcriptomic changes that characterize the osimertinib resistant state. Through CRISPR-based functional genomics screen targeting epigenetic regulators and transcription factors, we uncover a critical regulatory network featuring key members of the NuRD and PRC2 complexes that mediate resistance. Most critically, our screen identifies FOSL1 and JUN, two subunits of the AP-1 transcription factor within this network, as the most significant hits. Mechanistically, we demonstrate that *cis*-regulatory elements exhibiting altered chromatin accessibility in the resistant state are enriched for cognate AP-1 binding motifs, enabling AP-1 to orchestrate a gene expression program that underpins the druggable MEK/ERK signaling axis, potentially enhancing cell viability and fitness of resistant cells. Importantly, genetic depletion or pharmacological inhibition of AP-1 reinstates cellular and molecular sensitivity, and reverts resistance-associated phenotypes, such as epithelial-to-mesenchymal transition, upon anti-EGFR rechallenge by repressing AKT and ERK signaling. These findings provide novel insights into the epigenetic and transcriptional control of osimertinib resistance in *EGFR*-mutant NSCLC, highlighting AP-1 as a targetable vulnerability of resistance-related hallmarks and offering a promising avenue for developing resistance reversal strategies.

## Introduction

Lung cancer is the leading cause of cancer-related mortality worldwide. Non-small cell lung cancer (NSCLC), accounting for 85% of lung cancer cases, is frequently driven by oncogenic mutations in the epidermal growth factor receptor (EGFR) kinase domain. These mutations instigate ligand-independent activation of EGFR signaling and serve as primary targets for genotype directed therapy [1]. Osimertinib, a third generation EGFR tyrosine kinase inhibitor (EGFR-TKI), was developed to counteract resistance to earlier EGFR-TKIs and has been approved for the management of locally advanced or metastatic NSCLC [2]. Despite its initial clinical efficacy, the long-term success of osimertinib is limited by the emergence of resistance.

Genetically encoded resistance mechanisms are well-described, with on-target *EGFR* mutations impeding osimertinib binding to EGFR [3]. However, in contrast to earlier EGFR-TKIs, only a small fraction of resistant cases is mediated by EGFR-centric alterations [4]. Alternative mechanisms involve compensatory activation of bypass signaling pathways through MET amplification, FGFR activation, and AXL upregulation, along with oncogenic fusions [3, 4]. Phenotypic adaptations, particularly those linked to epithelial-mesenchymal transition (EMT) and small cell lung cancer transformation, further complicate the resistance landscape [3]. However, in many resistant tumors (>50%), particularly in cases where non-genetic regulation is implicated, the molecular basis of resistance remains elusive.

Epigenetic and transcriptional control of gene expression is a key enabler of drug resistance in several types of cancers [5]. In *EGFR*-mutant NSCLC, recent findings indicate that chromatin reprogramming, and transcription factors dynamically rewire transcriptomic landscape under therapeutic pressure, allowing tumor cells to evade anti-cancer treatment. Notably, dysregulation of Hippo signaling, interaction of EZH2/G9a, and upregulation of the neuroendocrine lineage transcription factor ASCL1 represent important non-genetic programs in reversible and intrinsic drug-tolerant cell states [6–9]. Furthermore, mammalian SWI/SNF chromatin remodeling complexes have been implicated in modulating chromatin accessibility and driving adaptive gene expression programs associated with osimertinib resistance [10, 11]. As such, components of these complexes represent potential therapeutic vulnerabilities in osimertinib resistant NSCLC. Therefore, a deeper understanding of the epigenetic and transcriptional drivers of resistance could reveal novel and effective treatment strategies.

Advances in high-throughput functional genomics and multi-omics approaches have dramatically expanded our ability to systematically interrogate the genetic and molecular determinants of cancer cell fitness [12, 13]. In this study, we undertake a comprehensive investigation of non-genetic aspects of acquired resistance to osimertinib. We establish and characterize an osimertinib resistant (OsiR) cell model derived from *EGFR*-mutant HCC827 cells, which recapitulate phenotypic and functional hallmarks of EGFR-TKI resistant models [10]. Through integrated ATAC-seq and RNA-seq analyses, we map chromatin accessibility and transcriptional changes revealing a significant interplay between epigenetic regulome and transcriptome of osimertinib resistant cells. A CRISPR-based screen targeting epigenetic regulators, transcription factors, and nuclear proteins (EpiTransNuc) informs key mediators of osimertinib resistance. Most notably, our screen identifies FOSL1 and JUN, two essential subunits of the AP-1 transcription factor complex, as high-confidence hits whose genetic depletion or pharmacological inhibition restores cellular and molecular sensitivity to osimertinib rechallenge. Mechanistically, AP-1 drives a transcriptional program that sustains resistance-related MEK/ERK signaling. Our study provides novel insights into the evolving non-genetic landscape of osimertinib resistance in *EGFR*-mutant NSCLC, establishing AP-1 transcription factor as a targetable determinant of state-specific hallmarks, offering therapeutic potential to reinstate drug sensitivity.

## Methods

### Cell lines and cell culture

HCC827, PC9 and H1975 cells were cultured in RPMI-1640 media (21875-034, Gibco) supplemented with 10% fetal bovine serum (FBS) (10500-064, Gibco) and 1% Penicillin/Streptomycin (10.000 U/mL) (15140-122, Gibco). Cells were maintained in a 5% CO_2_ atmosphere at 37 °C and passaged every 2–3 days with fresh media. HEK293T cells were cultured in high-glucose DMEM media (41965-039, Gibco) supplemented with 10% FBS and 1% Penicillin/Streptomycin. All cell lines were routinely screened for mycoplasma contamination using the PCR Mycoplasma Detection kit (G238, ABM). Only early passage cells were used in experiments.

### Generation of drug resistant cell line

The osimertinib resistant HCC827 subline, termed as HCC827-OsiR, was established using stepwise drug-escalation method. Initially, cells were exposed to osimertinib (HY-15772, MedChemExpress) at a sub-IC50 dose of 0.0015 μM. Surviving cells were cultured in drug-free RPMI-1640 medium to allow growth and recovery. The drug-free culture typically lasted 3 to 4 days, with extensions based on cell viability. In each successive round of osimertinib application, the dose was escalated by two-folds, and cells were subjected to drug-free conditions between treatments. Dose escalation continued until a maximum dose of 1.5 μM was reached. Cells demonstrating resistance at this dose were maintained in 1.5 μM osimertinib added media for an additional month. The entire process took ~6 months.

### IC50 calculations and MTT assay

Cellular sensitivity to EGFR-TKIs, namely osimertinib, dacomitinib (PZ0330, Sigma), and erlotinib (SML2156, Sigma), and the chemotherapeutic agent paclitaxel (S1150, Selleckchem), was assessed using MTT (1334GR001, Neofroxx). This assay measures cellular metabolic activity through the conversion of MTT to insoluble formazan crystals by NAD(P)H-dependent oxidoreductase enzymes. Cells were plated in six replicates in 96-well plates at various densities. After 24 h, cells were treated with a range of drug concentrations. Following 72 h of treatment, the media were aspirated and replaced with MTT containing media (5 mg/ml, final concentration: 0.5 mg/ml). After a 4 h incubation at 37 °C, the MTT solution was removed, and formazan crystals were dissolved in 100 μL of dimethyl sulfoxide (DMSO; 472301, Sigma) for 30–40 min on an orbital shaker. Absorbance was measured at 570 nm with background correction at 720 nm using a Multiskan GO Microplate Spectrophotometer (Thermo Fisher Scientific).

### Colony formation and anchorage-independent soft agar assays

Drug responses were evaluated using a 2D colony formation assay. Depending on the assay, cells were seeded at a density of 1500–5000 cells per well in triplicate onto 6-well culture plates. After allowing to adhere, cells were treated with predetermined doses of drugs for 7–12 days, with drug-containing media refreshed every 3–4 days. Following treatment, cells were fixed with 3.7% formaldehyde for 20 min and stained with 0.1% crystal violet (C6158, Sigma) dissolved in 10% ethanol for 40 min in the dark. The plates were rinsed with tap water, allowed to dry overnight at room temperature, and scanned with LI-COR Odyssey CLx Imaging System (LI-COR Biosciences). Analysis was performed using LI-COR Image Studio or ImageJ software.

For the anchorage-independent 3D growth assay, the bottom surface of 12-well plates was coated with a mixture of 1 mL of culture medium and 2% noble agar (w/v) (10907, Alfa Aesar) in equal ratios, which was allowed to solidify at room temperature for at least 30 min. HCC827 and HCC827-OsiR cells were then seeded into each well at a density of 10,000 cells/mL. This was achieved by mixing equal volumes of the growth medium and 0.6% noble agar (w/v). Subsequently, 1 mL of growth medium was added to each well, and cells were cultured for 3–4 weeks, with fresh medium containing SR11302 and/or osimertinib added every 3 to 4 days. The spheroids were stained with 0.1% crystal violet for 15 min and subsequently washed to enhance their visualization. Spheroids were observed using a light microscope (Olympus CKX41).

### Cell proliferation assay

Cells were seeded onto 18 mm coverslips placed in 12-well plates and incubated with osimertinib for 72 h. For proliferation assessment, cells were treated with 30 μM Bromodeoxyuridine (BrdU; B5002, Sigma) in drug-containing media for an additional 16 h. Cells were then fixed with 70% ice-cold ethanol for 10 min on ice, followed by incubation in 2 N HCl for 40 min at room temperature. After rinsing with PBS-T (PBS and %0.1 Tween 20), cells were labeled with anti-BrdU antibody (1:1,000 in 1% BSA in 1X PBS; 5292, Cell Signaling Technology) for 2 h at room temperature, followed by PBS-T washing and 1 h incubation with secondary antibody (1:1000, anti-mouse Alexa Fluor 488; 150105, Abcam). Coverslips were then incubated with DAPI, mounted onto slides, and visualized using fluorescence microscopy (BX61 Olympus). BrdU-labeled cells were quantified from at least five distinct microscopic areas and compared against DAPI-stained cells.

### Spheroid formation assays

To assess spheroid formation, the hanging drop cell culture method was utilized [14]. Briefly, FOSL1 or JUN knockout cells were treated with 1 μM osimertinib for 3 days prior to the formation of droplets. Then, in a 6-well cell culture plate, 20 μL suspensions containing 4,000 cells were added to the lid of each well, with 7 droplets per well. To prevent evaporation of the droplets, 1 mL 1X PBS was added to each well. The cells were incubated for 4 days to allow spheroid formation. Imaging was performed using a stereo microscope (Olympus SZX10).

To evaluate spheroid growth, ultra-low attachment 96-well plates were used [14]. Briefly, 200 μL of cell suspension containing 4000 cells was added to each well. The remaining wells were filled with 1X PBS to prevent evaporation. The culture plate was centrifuged at 800 rpm for 5 min to allow the cells to gather at the center. After a 4 day incubation period for spheroid formation, 100 μL of media was aspirated from each well, and an equal volume of osimertinib was added to initiate drug treatment. The drug treatment continued for 10 days. Spheroids were imaged using a light microscope (Olympus CKX41), and size analysis was quantitatively evaluated using ImageJ software.

### Cell cycle and apoptosis analysis

For cell cycle analysis, cells were detached from culture plates with Trypsin-EDTA (25200056, Gibco) and fixed for 15 min with ice-cold ethanol (final conc: 70%) added dropwise. Fixed cells were pelleted by centrifugation at 1200 rpm for 15 min at 4 °C and resuspended in 70 μL of propidium iodide (PI) mixture containing 50 μg/mL PI, 0.1 mg/mL RNaseA, and 0.05% Triton X-100 in 1X PBS. The cell suspension was incubated at 37 °C for 40 min with vortexing every 10 min. Stained cells were transferred into flow tubes with 100 μL of FACS Buffer and analyzed using a flow cytometer (LSR Fortessa, BD).

For apoptosis analysis, cells were collected and stained using the FITC Annexin V Apoptosis Detection Kit with PI (640914, Biolegend). Briefly, cells were stained in dark with 36 μL of staining solution (2 μL of Annexin V-FITC and 4 μL of PI solution in 30 μL binding buffer) for 15 min at room temperature. Following staining, 120 µL of Annexin V Binding Buffer was added to each tube, and samples were transferred to flow tubes for analysis using LSR Fortessa. Cell cycle and apoptosis data were processed with FlowJo v10.8.0 software (BD Biosciences).

### Immunofluorescence staining

Cells were seeded onto coverslips and allowed to grow until reaching ~80% confluency. They were then rinsed with 1X PBS and fixed with 3.7% formaldehyde for 10 min at room temperature. Cells were then permeabilized with 0.5% Triton X-100 for 5 min. After washing and blocking steps, cells were incubated with the primary antibodies (Supplementary Table 1) for 1 h at room temperature. This was followed by a 1 h incubation with the secondary antibodies (Supplementary Table 1). Nuclei were stained with DAPI and visualized using confocal microscopy (LSM880, Zeiss).

### Western blotting and proteome profiling

Cells were lysed on ice using a modified RIPA buffer solution [50 mM Tris pH 8.0, 150 mM NaCl, 1% Triton X-100, 0.5% Na-deoxycholate, 0.1% SDS, 1 mM EDTA, 1 mM EGTA, 10 mM β-glycerophosphate, 10 mM NaF, 1 mM PMSF, 1 mM Na-orthovanadate, Leupeptin, Aprotinin, and Pepstatin (each 10 μg/mL)] for 30 min. Lysates were centrifuged at 13,300 rpm for 30 min at 4 °C, and the supernatants were collected and stored at -80 °C. Protein concentrations were measured using the Pierce BCA Protein Assay Kit (23227, Thermo Scientific), following the manufacturer’s protocol.

For western blotting, samples were loaded onto SDS-PAGE gels (10-12% gradient) and subjected to electrophoresis. Proteins were transferred onto 0.2 μm nitrocellulose membrane (10600004, GE Healthcare) using a wet-transfer method at 350 mA for 90 min. After blocking with 5% skimmed milk (70166, Sigma) or 5% BSA (1126GR100, Neofroxx) in 1X TBS for 1 h at room temperature, the membranes were incubated with primary antibodies at 4 °C overnight. The membranes were rinsed with TBS-T (Tris-buffered saline and 0.1% Tween 20 (BP337, Fisher BioReagents)), followed by incubation with secondary antibodies for 1 h at room temperature. Primary and secondary antibodies, and their working conditions, are listed in the Supplementary Table 1. Protein levels were detected using the LI-COR Odyssey Imaging System.

Changes in intracellular signaling pathways were explored using the Human Phosphorylation Pathway Profiling Array C55 (AAH-PPP-1-2, RayBiotech), according to the manufacturer’s instructions. In brief, nitrocellulose membrane arrays were incubated with diluted cell lysates (250 μg protein in 1 mL solution) overnight at 4 °C, followed by a 2 h incubation with the antibody cocktail. After thorough rinsing, the arrays were treated with HRP-conjugated secondary antibodies for 2 h at room temperature. Dots were visualized with the Chemiluminescence system (ChemiDoc MP, Bio-Rad), and analyzed by ImageJ software with Protein Array Analyze tool. Statistical calculations of the images were performed with the analysis tool provided by the manufacturer.

### RNA Isolation, cDNA synthesis and qRT-PCR

Total RNA was isolated using the NucleoSpin RNA kit (740955, Macherey-Nagel), following the manufacturer’s instructions. RNA concentrations were quantified using a NanoDrop 2000 spectrophotometer (ND-2000, Thermo Fisher Scientific), and samples were stored at -80 °C. cDNA synthesis was performed using the iScript cDNA Synthesis Kit (1708891, BioRad). Expression levels of target genes were analyzed through quantitative real-time reverse transcriptase PCR (qRT-PCR), with β-actin or GAPDH serving as housekeeping genes. qRT-PCR reactions were performed on an ABI Prism 7500 Fast Real-Time PCR machine, using BlasTaq Green 2X qPCR Master mix (G892, ABM). Relative gene expression was calculated with the 2^–ΔΔCt^ method. A complete list of oligonucleotides is provided in Supplementary Table 2.

### Molecular cloning

Guide RNA (gRNA) sequences were cloned into lentiviral vectors, lentiCRISPR v2 (Addgene plasmid #52961) or lentiGuide-Hygro-dTomato (Addgene plasmid #99376), depending on the experimental context. A gRen targeting *Renilla* luciferase was used as a non-human targeting control. Oligonucleotides (Supplementary Table 3) were annealed and ligated into predigested backbones, followed by transformation into Stbl3 competent bacteria (C3040, NEB). For RNA interference, shRNA sequences targeting specific genes were obtained from the RNAi Consortium and cloned into the pLKO.1 puro backbone (Addgene plasmid #8453). Target sequences for FOSL1 and JUN shRNAs were TRCN0000019541 (CCTCAGCTCATCGCAAGAGTA) and TRCN0000010366 (TAGTACTCCTTAAGAACACAA), respectively. Synthesized oligonucleotide pairs were annealed and ligated into the AgeI (R3552S, NEB) and EcoRI (R3101S, NEB) sites of pLKO.1. Vectors were propagated in Stbl3. pLKO.1 puro scrambled shRNA (shSCR, Addgene Plasmid #1864) was used as a non-targeting control. All constructs were verified by Sanger sequencing.

### Lentivirus production and transduction

Stable Cas9 expressing HCC827-OsiR cells were generated using lentiCas9-Blast vector (Addgene plasmid #52962). HEK293T cells were seeded at a density of 1 × 10^6^ in 6-well plates. Lentiviral particles were produced by co-transfecting HEK293T cells with psPAX2 (Addgene plasmid #12260) and pMD2.G (Addgene plasmid #12259) helper plasmids at an optimized 4:2:1 ratio (Target vector:psPAX2:pMD2.G), using polyethylenimine (PEI). Viral supernatants were collected at 48 h or 72 h post transfection, filtered through 0.45 μm SFCA filters (431220, Corning), and either used immediately or stored at -80 °C. Infected cells were selected with Blasticidin (15 μg/mL, ant-bl-05, Invivogen) for 6 days, and Cas9 expression was confirmed by western blotting and functional assays. This protocol was similarly applied for packaging other lentiviral vectors based on lentiCRISPR v2, pECPV or pLKO.1 backbones. For large-scale virus production, cell number, plasmid and PEI quantities were scaled accordingly. Puromycin (1 μg/mL, ant-pr-1, Invivogen) was used for the selection of clones generated using lentiCRISPR v2 or pLKO.1.

### EpiTransNuc gRNA library design and construction

The EpiTransNuc gRNA library, enriched for epigenetic regulators, transcription factors, and nuclear proteins, was constructed using the lentiGuide-Puro backbone (Addgene plasmid #52963). This custom-built library is improved upon the “nuclear” gRNA sub-pool of the Human CRISPR knockout libraries [15], and consists of 40,820 gRNAs, averaging 10 gRNAs per gene, with an additional 100 non-targeting control gRNAs. Oligonucleotides compatible with BsmBI restriction sites were synthesized by CustomArray (GenScript), incorporating 5’ (TATCTTGTGGAAAGGACGAAACACCG) and 3’ flanking sequences (GTTTTAGAGCTAGAAATAGCAAGTTAAAAT). Library assembly was achieved using the CPEC (Circular Polymerization Extension Cloning) method as previously described [16, 17]. Briefly, backbone linearization was performed by PCR with primers: Forward (GTTTTAGAGCTAGAAATAGCAAGTTAAAATAAGGCTAGTCCGTTATCAACTTGAAAAAGTGGCACC), and Reverse (CGGTGTTTCGTCCTTTCCACAAGATATATAAAGCCAAGAAATCGAAATACTTTCAAGTTACGG). Ten reactions were pooled, and the digested linear backbone was treated with BsmBI to minimize gRNA-free clones. Amplification of the EpiTransNuc library was carried out using NEBNext High-Fidelity 2X PCR Master Mix (M0541S, NEB), with forward (GTAACTTGAAAGTATTTCGATTTCTTGGCTTTATATATCTTGTGGAAAGGACGAAACACC) and reverse (ACTTTTTCAAGTTGATAACGGACTAGCCTTATTTTAACTTGCTATTTCTAGCTCTAAAAC) primers. PCR cycle number was limited to 20 to reduce gRNA representation bias. Following CPEC-mediated cloning, 600 ng of the plasmid library was electroporated into Endura electrocompetent bacteria (60242-1, Lucigen) using the BioRad MicroPulser. Bacteria were spread on two 500 cm² bioassay dishes (240835, Thermo Fisher) and cultured for 18 h at 37 °C, and plasmids were isolated with the Endotoxin free HiSpeed Plasmid DNA Maxi kit (12362, Qiagen). The final library had an estimated coverage of 45x, and gRNA representation was assessed using next generation sequencing (NGS), followed by analysis with *count_spacers.py* script [18].

### CRISPR screening and experimental procedure

For large-scale packaging of the EpiTransNuc library, HEK293T cells (5 × 10^6^) were seeded into four T75 flasks. A total of 33.2 μg of the gRNA library, 16.6 μg of psPAX2 vector, and 8.3 μg of pMD2.G were co-transfected into HEK293T cells using PEI at a DNA/PEI ratio of 1:5 (wt/wt) [14]. Viral supernatants were collected, filtered, and aliquots were stored at -80 °C until infection. Screening experiments were conducted by transducing HCC827-OsiR-Cas9 cells (168 × 10^6^) at a multiplicity of infection (MOI) of ~0.3–0.4 for 48 h, maintaining 600× coverage ( ~ 24 × 10^6^ cells). Infected cells were selected with Puromycin (1 µg/mL) for 5 days, after which the initial population (T0, at least 24 × 10^6^) was harvested. Cells ( ~ 24 × 10^6^ per group) were seeded into a total of 96 dishes (150 mm) at a density of ~1.5 × 10^6^ cells per dish and divided into control or osimertinib-treated groups (IC_20_: 1 μM) with three replicates per condition. Cells were cultured for 24 days, with re-seeding every 3–4 days. Cell pellets were collected at the final time point (T24).

### Genomic DNA extraction, targeted DNA sequencing and data processing

Genomic DNA (gDNA) was extracted from T0 and T24 cell pellets using the QIAamp DNA Blood Maxi kit (51194, QIAGEN) per the manufacturer’s protocols. PCR amplification was performed with 50 μg of input gDNA using NebNext High Fidelity 2X Master Mix (2X) (M0541L, NEB) and Illumina TruSeq adapters P5 and P7. Each PCR reaction included common P5 forward primers and reverse primers with different index regions (i1 - i7). PCR products were verified by agarose gel electrophoresis, excised, purified with the NucleoSpin Gel and PCR Clean-up Kit (740609, Macherey-Nagel), and quantified using NanoDrop2000. Libraries were pooled in equimolar quantities and sequenced on Illumina HiSeq X platform (Macrogen, Seoul, Republic of Korea). Sequencing data were processed using the Cutadapt tool (v3.7) to trim low-quality reads, and gRNA sequences were mapped to the EpiTransNuc library using the MAGeCK algorithm (v0.5.9) [19]. Read counts were normalized, and differentially enriched gRNAs were identified via Robust Rank Aggregation (RRA) analysis. Maximum-Likelihood Estimation (MLE) method was used to assess gene essentiality, based on beta-scores. Quality control and integrative analyses were performed using the MAGeCKFlute algorithm [20]. A CRISPR fitness score was calculated for each gene (treatment vs control) by normalizing the median of z-score-transformed logFC in the relative abundance of gRNAs targeting the gene. Z-scores were derived using the following formula:

$${\rm{z}}-\mathrm{score}=\frac{\left(\mathrm{gRNA}\,\mathrm{fold}\,\mathrm{change}\right)-\mathrm{mean}\left(\mathrm{gRNA}\,\mathrm{fold}\,\mathrm{change}\right)}{\mathrm{Standard}\,\mathrm{deviation}\left(\mathrm{gRNA}\,\mathrm{fold}\,\mathrm{change}\right)}$$

### Competitive cell proliferation assay

Competitive cell proliferation assays were conducted in HCC827-OsiR-Cas9 cells, with lentiGuide-Hygro-dTomato vector. The distribution of cells expressing dTomato fluorescent protein was monitored by flow cytometry at regular intervals. This enabled the validation of genes whose depletion restores sensitivity to osimertinib. gRNA targeting essential gene *RPA3* served as a positive control.

### Wound healing and invasion assays

For the wound healing assay, cells were seeded at 300,000 cells/well in 6-well plates and grown to near confluence. A vertical scratch was made using a 200 μL pipette tip, and after washing twice with PBS, RPMI-1640 containing 2% serum and osimertinib was added. Wound closure was monitored over consecutive days. For the invasion assay, 24-well culture inserts (37224, SPL Life Sciences) were coated with Matrigel (1:40 in serum-free media, 356234, Corning) and incubated at 37 °C for 2 h. After removing the coating media, cells (50,000 in 200 μL serum-free medium) were seeded onto the upper layer. The lower chamber was filled with 700 μL of RPMI-1640 with 10% FBS. After 24 h, the medium was aspirated, and cells were fixed with 3.7% formaldehyde for 2 min, permeabilized with absolute methanol for 30 min, and stained with crystal violet for 15 min. Non-invading cells were gently removed with a cotton swab and allowed to air-dry. Invaded cells were imaged under a light microscope (Olympus CKX41) and quantified by ImageJ.

### RNA sequencing and analysis

Transcriptome profiling of HCC827 and HCC827-OsiR samples (triplicates), and *FOSL1* or *JUN* knockout cells relative to gRen control HCC827-OsiR clone (duplicates), were performed on the Illumina NovaSeq 6000 platform (Macrogen, Seoul, Republic of Korea). Over 100 million paired-end reads (151-bp) per sample were generated. Quality control was performed using FASTQC (v0.11.7), and low-quality reads were removed from the transcriptome libraries using Cutadapt (v3.7). Remaining reads were aligned to the GRCh38 human reference genome (Gencode release 34) using the Rsubread package (v1.34.7). Gene expression levels were quantified using the featureCounts function of Rsubread, and converted to counts per million (CPM). Differential expression analysis between groups was performed using the edgeR package (v3.24.3) in the R computational environment (v3.5.2). Gene expression data were visualized using the ggplot2 package in the R environment. Genes with significant expression changes were listed based on adjusted *p*-values (adj-*p*-val < 0.05), with various visualizations generated to represent significant gene sets.

### ATAC sequencing and analysis

ATAC sequencing samples were prepared using an ATAC-seq kit (53150, Active Motif) and following a modified protocol [21]. Briefly, 100,000 freshly cultivated HCC827 and HCC827-OsiR cells (duplicates) were resuspended in 100 μL ice-cold ATAC Lysis Buffer for nuclei extraction. The cell pellet was resuspended and transferred to PCR tubes on ice, followed by centrifugation at 500 g for 10 min at 4 °C. Samples were tagmented with 50 μL Tagmentation master mix containing assembled transposons and incubated at 37 °C for 30 min. Post-tagmentation, 250 μL DNA Purification Binding Buffer and 5 μL of 3 M sodium acetate were added. Samples were transferred to kit supplied columns and eluted in 35 μL of elution buffer. The tagmented DNA was PCR amplified using indexed i5 and i7 primers. Index primers i7 N703 and i5 (N501 and N502) were used for HCC827 duplicates, while i7 N703 and i5 (N503 and N504) were used for HCC827-OsiR duplicates. Sequencing was performed on the Illumina NovaSeqX platform (Macrogen, Seoul, Republic of Korea), generating over 50 million paired-end reads (151 bp) per sample.

ATAC-seq data were aligned to the GRCh38 human reference genome using the nf-core/atac-seq pipeline v2.0 [22] (https://nf-co.re/atacseq/2.1.2, accessed on 01 April 2024). Broad peaks were called with MACS2, and peak annotation was performed. Differentially accessible regions were identified using the DiffBind package and the DESeq2 algorithm. Statistically significant differentially accessible regions were defined by an absolute log2 fold-change (log2FC) greater than a chosen threshold of 0.5 or 1, with a *p*-value of <0.05 across all comparisons between the two cell states. Gene Ontology enrichment analysis was performed using the R package ClusterProfiler v3.19. ATAC-seq tracks were visualized using Integrated Genomic Viewer (IGV2.12.3). Overlapping regions between ATAC-seq peaks and GeneHancer database were identified using the GenomicRanges package (v1.56.1) with the findOverlaps function. Motif enrichment analysis was subsequently performed on these regions using the HOMER (Hypergeometric Optimization of Motif EnRichment) tool (v5.1) [23].

### Gene-set enrichment analysis

Gene Set Enrichment Analysis (GSEA) was performed as previously described [24]. Instead of evaluating individual gene expressions, this approach assesses the significance of collective gene movements sharing common attributes. The HCC827 and HCC827-OsiR groups were analyzed using the GSEA tool from the Broad Institute. A list of expressed genes with phenotype labels was uploaded to the GSEA tool, and normalized enrichment scores (NES), nominal *p*-values (*p*), and false discovery rates (FDR) were calculated after 1000 random permutations of the gene set. Bubble chart visualization and further analysis were carried out using Bubble GUM (GSEA Unlimited Map) software. Statistically significant results were defined as NES > 1, *p* < 0.05, and FDR < 0.25.

### Animal experiments

HCC827 cells (5 × 10^6^), mixed with Matrigel (1:1 ratio, vol/vol) in 100 μL, were subcutaneously injected into the right and left flanks of 7–8 week-old female athymic (nu/nu) mice (*n* = 4). Osimertinib was administered at 25 mg/kg daily, 5 days a week via oral gavage, beginning when tumor volumes reached ~75 mm^3^. Mice were randomized into control and drug treatment groups. Control groups received the vehicle (5% DMSO, 40% PEG400, 5% Tween-80, and 50% PBS). Tumor volumes were measured every 2–3 days using a digital caliper. All xenograft experiments were conducted at the IBG Vivarium with approval from the Ethics Committee for Animal Experiments of Izmir Biomedicine and Genome Center (IBG-HADYEK, protocol number: 2022-035).

### Statistical analysis

Data were presented as mean ± standard deviation (SD). For comparisons between two conditions or groups, a two-tailed unpaired Student’s *t*-test was used. For more than two groups, one-way or two-way analysis of variance (ANOVA) followed by Dunnett’s multiple comparisons test was used. Correlations of gene expression were analyzed using the Pearson correlation test. Specific details on statistical significance are provided in the figure legends. Statistical analyses were performed using GraphPad Prism version 8.0 (GraphPad Software, San Diego, USA). A *p*-value of <0.05 was considered statistically significant, indicated as *p < 0.05, **p < 0.01, ***p < 0.001.

## Results

### Osimertinib resistant cells display functional and phenotypic hallmarks of EGFR-TKI-resistant cancer cells

**Fig. 1:**
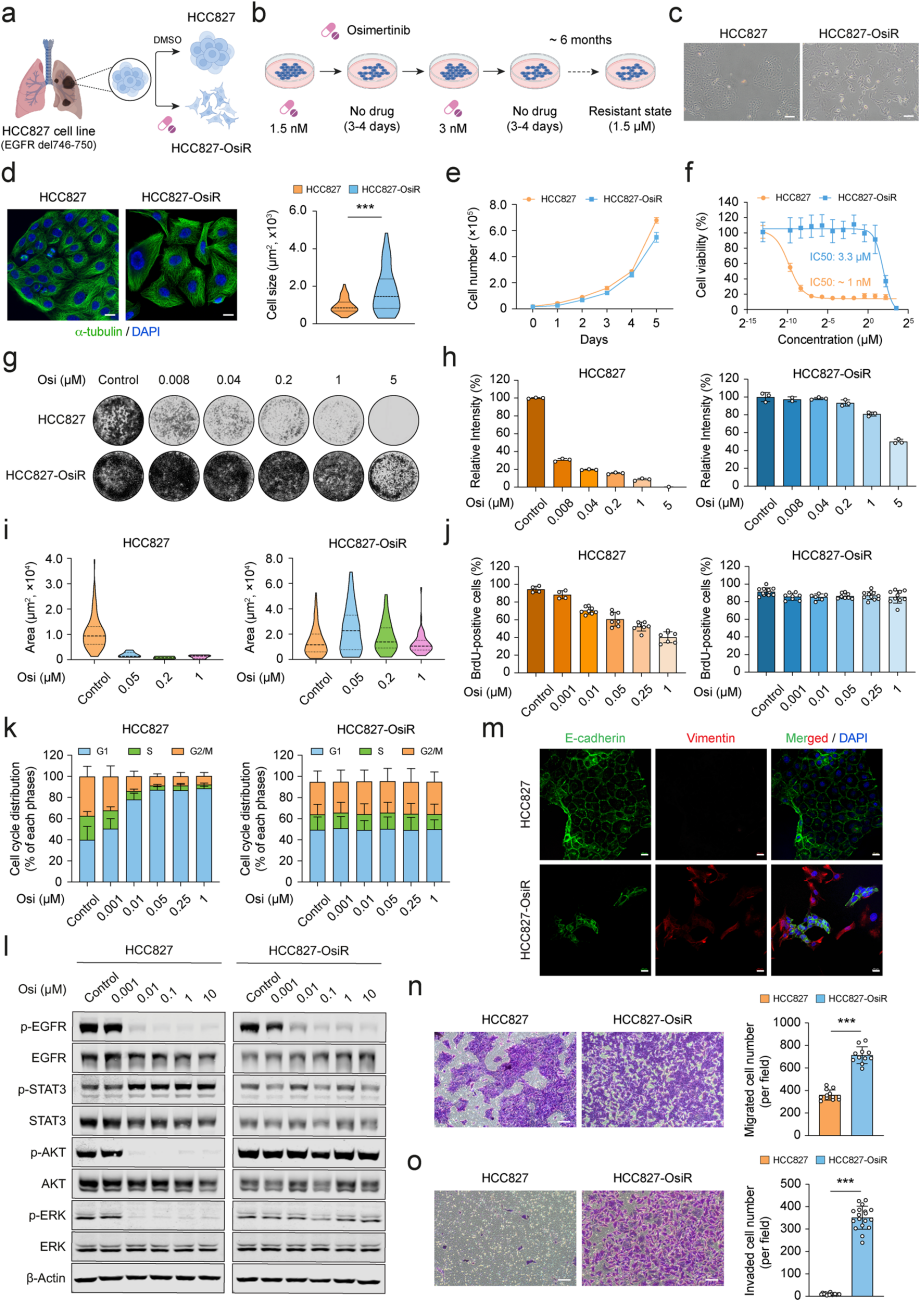
Establishment and characterization of osimertinib resistant NSCLC cells. **a**, **b** Schematic representation for the generation of osimertinib resistant cells. HCC827 cells were chronically exposed to osimertinib using a stepwise drug-escalation approach, where drug doses were doubled in each round. Control cells were treated with DMSO as a vehicle. **c** Morphological differences between HCC827 and HCC827-OsiR cells were observed under light microscopy (magnification 20×, scale bar: 50 μm). **d** Representative immunofluorescence (IF) images of α-tubulin staining (magnification 40×, scale bar: 20 μm). DAPI served as nuclear counterstain (blue). Cellular dimensions (μm^2^) were quantified for HCC827 (*n* = 62) and HCC827-OsiR cells (*n* = 147). Significance was calculated using an unpaired Student’s *t*-test. ^***^p < 0.001. **e** Proliferation dynamics of HCC827 and HCC827-OsiR cells (*n* = 4). **f** Osimertinib dose-response curves comparing IC50 values for HCC827 and HCC827-OsiR cells (*n* = 6). **g** Representative images of crystal violet colony formation assays conducted in 6-well culture plates. **h** Quantification of 2D colony formation data from independent replicates (*n* = 3). **i** Quantification of 3D anchorage-independent soft agar data (representative images are shown in Fig. S1h). Colony areas were quantified for HCC827 (data range, *n* = 6-89) and HCC827-OsiR cells (data range, *n* = 59-195). **j** Osimertinib dose-response of DNA synthesis measured by BrdU incorporation assay. Representative images are provided in Fig. S1k. **k** Osimertinib dose-response of cell cycle phase distribution from independent replicates (*n* = 3). **l** Western blots of EGFR and alternative signaling pathways after 6 h of osimertinib treatment. β-actin was used as a loading control. **m** Representative IF images displaying E-cadherin and Vimentin distribution (magnification 25×, scale bar: 20 μm). DAPI served as nuclear counterstain (blue) **n**, **o** Representative images of migration and transwell invasion assays for HCC827 and HCC827-OsiR cells (magnification 10×, scale bar: 100 μm). Quantification of migrated and invaded cell numbers per field is shown on the right. Data are presented as mean ± SD. ^***^p < 0.001.

To model EGFR-TKI resistance in vitro, we developed an acquired osimertinib resistant subline derived from HCC827 NSCLC cells, which harbor an activating *EGFR* mutation (exon 19, del746–750). These cells are inherently sensitive to osimertinib, as confirmed by in vitro assays and in vivo xenograft models (Supplementary Fig. 1a–c). Genetic targeting of EGFR confirmed their over-reliance on EGFR signaling (Supplementary Fig. 1d–f). Resistance was induced by chronic exposure to osimertinib, exploiting a dose-escalation strategy with alternating cycles of drug-free or drug-containing media (Fig. 1a, b). The resulting line, dubbed HCC827-OsiR, acquired resistance to a clinically relevant concentration of osimertinib (1.5 µM) [25]. Immediate comparison with drug-naive cells revealed striking morphological changes, including axon-like cell membrane protrusions (Fig. 1c), increased cellular dimensions (Fig. 1d), and variability in nuclear size (Supplementary Fig. 1g). While growth kinetics remained comparable to parental cells (Fig. 1e), HCC827-OsiR cells lost EGFR dependency, evidenced by >1000-fold increase in osimertinib IC50 (resistance index (RI): ~3500) (Fig. 1f) and confirmed by CRISPR-knockout of *EGFR* (Supplementary Fig. 1d–f). Resistant cells displayed robust survival under osimertinib pressure in monolayer (2D) and spheroid (3D) cultures, although they were relatively more sensitive in the 3D environment (Fig. 1g–i, Supplementary Fig. 1h). HCC827-OsiR cells were also cross-resistant to earlier EGFR-TKIs (Supplementary Fig. 1i), with minimal differential response to the microtubule stabilizing agent paclitaxel [26] (Supplementary Fig. 1j). As expected, these cells sustained proliferative capacity despite drug exposure (Fig. 1j and Supplementary Fig. 1k). Unlike HCC827 cells, which experienced dose-dependent G1 phase arrest by osimertinib treatment, resistant cells were largely unaffected (Fig. 1k and Supplementary Fig. 2a, b). Additionally, although osimertinib effectively induced apoptosis in parental cells (Supplementary Fig. 2c), resistant cells showed minimal response, even at higher doses of the drug (Supplementary Fig. 2d).

At the molecular level, the resistant state was marked by a pronounced reduction in EGFR phosphorylation and expression (Supplementary Fig. 2e), with no evidence of on-target *EGFR* mutations (Supplementary Fig. 2f). Similar to other EGFR-TKI resistance models, we observed hyperactivation of compensatory pathways, particularly AKT and ERK signaling (Supplementary Fig. 2e and Fig. 1l). Phosphorylation proteome profiling further confirmed the activation of multiple alternative signaling mechanisms (Supplementary Fig. 3a). Crucially, the acquisition of resistance was associated with EMT-related traits, such as altered protein levels and cellular patterns of e-cadherin and vimentin (Fig. 1m and Supplementary Fig. 3b). These changes correlated with the expression profiles of EMT signature genes (Supplementary Fig. 3c). Functionally, the resistant state was defined by increased cell motility, migration, and invasive potential (Supplementary Fig. 3d and Fig. 1n, o). Lastly, clonal sublines of HCC827-OsiR cells revealed notable heterogeneity in resistance, yet all clones exhibited consistent activation of bypass signaling pathways and acquisition of mesenchymal phenotype (Supplementary Fig. 4). These data provide compelling evidence for the successful establishment and comprehensive characterization of osimertinib resistant HCC827-OsiR cells, which faithfully recapitulate the functional and phenotypic hallmarks of EGFR-TKI-resistant models.

### Integrative analysis of chromatin accessibility and transcriptome landscapes reveals gene expression programs that characterize osimertinib resistant cells

**Fig. 2:**
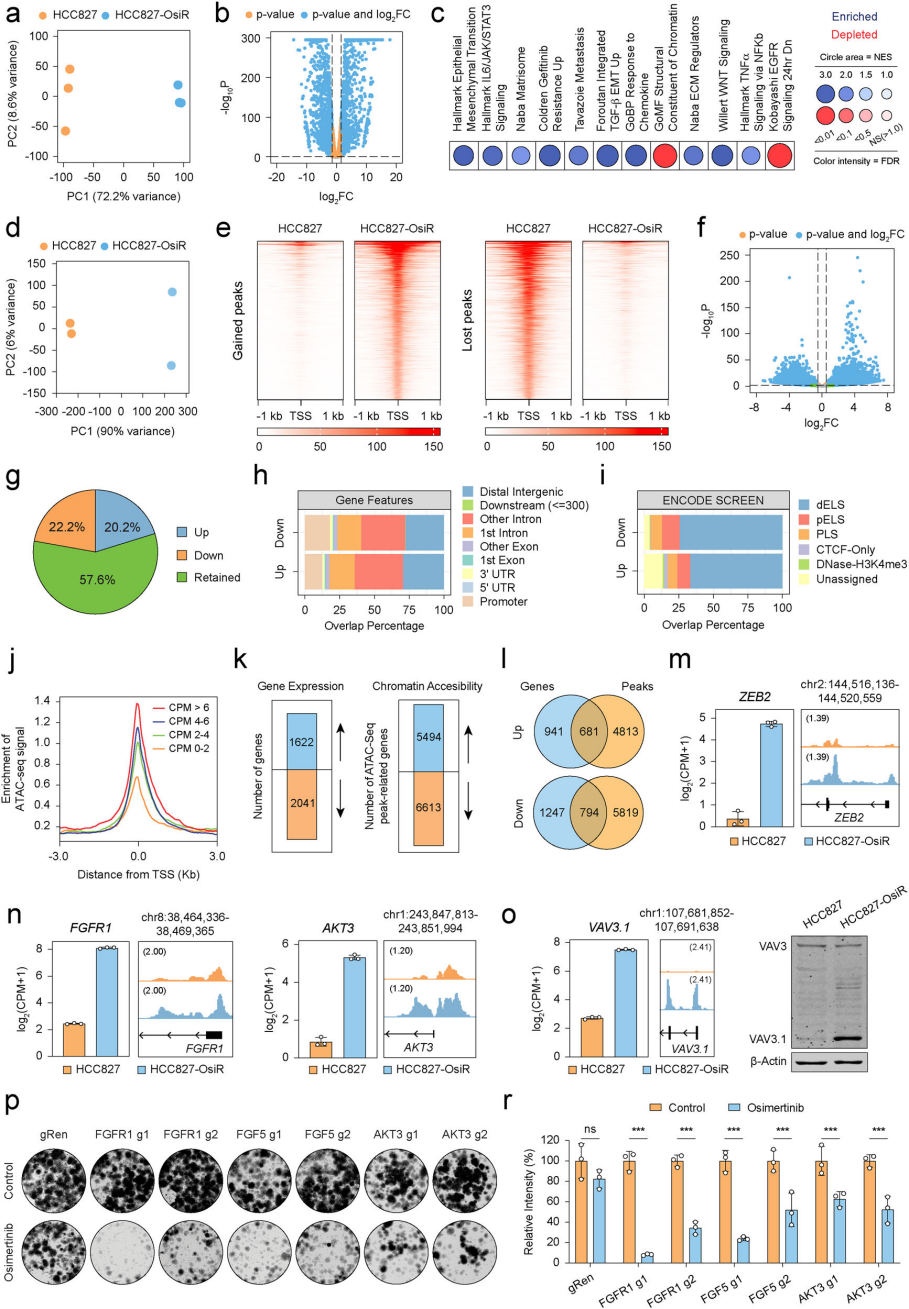
Epigenetic and transcriptomic landscape of osimertinib sensitive and resistant states. **a** Principal component analysis (PCA) plot displaying variance (%) in transcriptome data of HCC827 and HCC827-OsiR cells. Each dot represents a replicate. **b** Volcano plot illustrating differential gene expression patterns between HCC827 and HCC827-OsiR cells. **c** Gene set enrichment analysis (GSEA) visualized using BubbleGUM, depicting pathway enrichment for enriched and depleted gene signatures in HCC827 and HCC827-OsiR cell lines. Circle area indicates NES, and color intensity represents FDR. NS, not significant. **d** PCA plot showing variance (%) in chromatin accessibility changes between HCC827 and HCC827-OsiR. Each dot represents a replicate. **e** Heatmap representation of ATAC-seq gained and lost peaks in HCC827 and HCC827-OsiR cells. TSS, transcription start site. Colored bar represents *z-score* calculations. **f** Volcano plot showing differential changes in chromatin accessibility of the resistant state (HCC827-OsiR vs HCC827). **g** Pie chart representing the proportion of altered (up or down) and retained ATAC-seq peaks in HCC827-OsiR cells compared to HCC827. **h** Distribution of chromatin accessible regions annotated by gene features for significantly gained (up) or lost (down) peaks. **i** Overlap percentages of gained (up) or lost (down) ATAC-seq tracks annotated by ENCODE SCREEN regulatory elements (Registry V3) (dELS: distal enhancer like signatures, pELS: proximal enhancer like signatures, PLS: promoter like signatures, CTCF-only: CCCTC-Binding factor only). **j** Enrichment of ATAC-seq signals around TSS of differentially expressed genes (HCC827-OsiR vs HCC827), grouped by average log_2_(CPM + 1) values. **k** The number of differentially expressed genes and ATAC-seq peak-related genes. Blue color represents upregulated genes and gained peaks, while orange color denotes downregulated genes and lost peaks. **l** Proportion of the intersection between differentially expressed genes and ATAC-seq peak-associated genes for both upregulation and downregulation. **m**, **n** ATAC-seq tracks over the *ZEB2* (**m**) *FGFR1* and *AKT3* (**n**) loci in HCC827 and HCC827-OsiR cells. Gene expression log_2_(CPM + 1) values are displayed in bar graphs. Error bars represent the mean ± SD of the expression level for each cell line. **o** ATAC-seq tracks over the *VAV3.1* variant in the HCC827 and HCC827-OsiR cells. Gene expression log_2_(CPM + 1) values are displayed in bar graphs (left). Error bars represent the mean ± SD of the expression level for each cell line. Western blot of VAV3 and VAV3.1 in HCC827 and HCC827-OsiR cells. β-actin was used as a loading control (right). **p** Representative images of crystal violet colony formation assay in *FGFR1*, *FGF5* and *AKT3* depleted clones, in the absence or presence of osimertinib (1 μM). Each gene is targeted by two unique gRNAs. gRen served as a negative control. **r** Quantification of 2D colony formation data for gRen, *FGFR1*, *FGF5*, and *AKT3* knockout clones in the absence or presence of osimertinib from independent replicates (*n* = 3). Each control is normalized to 100 and data are presented as mean ± SD. ^***^p < 0.001, ns not significant.

To explore gene expression alterations underpinning osimertinib resistance, we performed comparative RNA-seq between drug-sensitive and resistant states. Principal component analysis (PCA) and heatmap visualization revealed substantial changes in the transcriptomic landscape of resistant cells (Fig. 2a and Supplementary Fig. 5a). A total of 3665 genes were differentially expressed, with 44.3% upregulated and 55.7% downregulated in HCC827-OsiR cells (Fig. 2b). These changes included a plethora of genes implicated in EGFR-TKI resistance in NSCLC patients and experimental models (Supplementary Fig. 5b), corroborating findings from earlier studies [27–30]. Gene set enrichment analysis (GSEA) revealed enrichment of hallmark gene signatures related to EMT, extracellular matrix regulators and matrisome, and metastatic potential, consistent with the motile and invasive nature of resistant cells (Fig. 2c and Supplementary Fig. 5c). Moreover, TGF-β, IL6/JAK/STAT3, TNF/NF-kB, and WNT signaling pathways were significantly enriched in the resistant state (Fig. 2c and Supplementary Fig. 5d). In contrast, signatures for EGFR signaling and epithelial gene expression were selectively enriched in the parental state (Fig. 2c and Supplementary Fig. 5e). Notably, gene sets pertaining to reprogramming of chromatin and histone landscapes were also significantly altered in the resistant state (Fig. 2c and Supplementary Fig. 5f), suggesting extensive gene regulatory changes during the acquisition of osimertinib resistance.

To interrogate the role of chromatin and transcriptional plasticity in shaping the resistant transcriptome, we performed ATAC-seq. The analysis of genome-wide chromatin accessibility alterations revealed distinct chromatin landscapes between parental and resistant cells (Fig. 2d), underscoring the role of transcriptional reprogramming in driving resistance. Notably, regions that lost accessibility in HCC827-OsiR cells exhibited slightly larger effect sizes, indicative of pronounced chromatin silencing (Fig. 2e). Differential peak analysis uncovered 42.4% of ATAC signals corresponding to regions that either gained or lost chromatin accessibility in the resistant state (Fig. 2f, g). Interestingly, gained ATAC-seq signals were predominantly located in intronic or intergenic regions, where enhancers or other *cis*-regulatory elements are typically located, as opposed to proximal promoters which showed reduced peak intensity (Fig. 2h). Furthermore, we observed increased enrichment of ENCODE SCREEN annotated distal enhancers within regions of gained or lost chromatin accessibility compared to all peaks (Fig. 2i), suggesting a critical role for these elements in modulating gene expression in resistant cells. Interestingly, GO enrichment analysis of genes with promoters overlapping gained accessibility regions revealed pathways involved in intracellular signaling and EMT-related processes, while genes associated with lost accessibility regions were mainly enriched for processes related to cellular architecture and protein tyrosine kinase activity (Supplementary Fig. 5g).

To further dissect this phenomenon, we performed an integrative analysis of ATAC-seq and RNA-seq data. As expected, ATAC signals enriched at transcription start sites (TSS) strongly correlated with gene expression levels in HCC827-OsiR cells (Fig. 2j). Remarkably, over 40% differentially expressed genes concordantly overlapped with the genes linked with significant ATAC-seq peaks located at promoter regions or gene regulatory elements, involved in the regulation of the respective genes (Fig. 2k, l, Supplementary Fig. 6a). Analysis of the gene expression-regulation axis identified key molecules associated with hallmark traits of resistant cells, including upregulation of mesenchymal signature genes, functional enzymes, and RTK signaling pathways (Fig. 2m, n, Supplementary Fig. 6b and 6c), along with downregulation of epithelial signature genes (Supplementary Fig. 6d). Visualization of ATAC-seq tracks also identified resistant state-specific differentially expressed long non-coding RNAs (e.g., SFTA1P, MIR200CHG, and DOCK8-AS1) and protein coding transcript variants (e.g., VAV3.1) (Fig. 2o, Supplementary Fig. 6e, f). Most critically, chromatin-mediated positive regulation of gene expression was strongly associated with resistance activity, which could be validated by CRISPR depletion of select genes (Fig. 2p and r, and Supplementary Fig. 6c, g and h). Together, these findings suggest that dynamic rewiring of chromatin-mediated transcriptional programs between drug-sensitive and resistant states modulates the enhancer/promoter-gene regulation axis that characterizes osimertinib resistance.

### Focused CRISPR screen uncovers mediators of osimertinib resistance

**Fig. 3:**
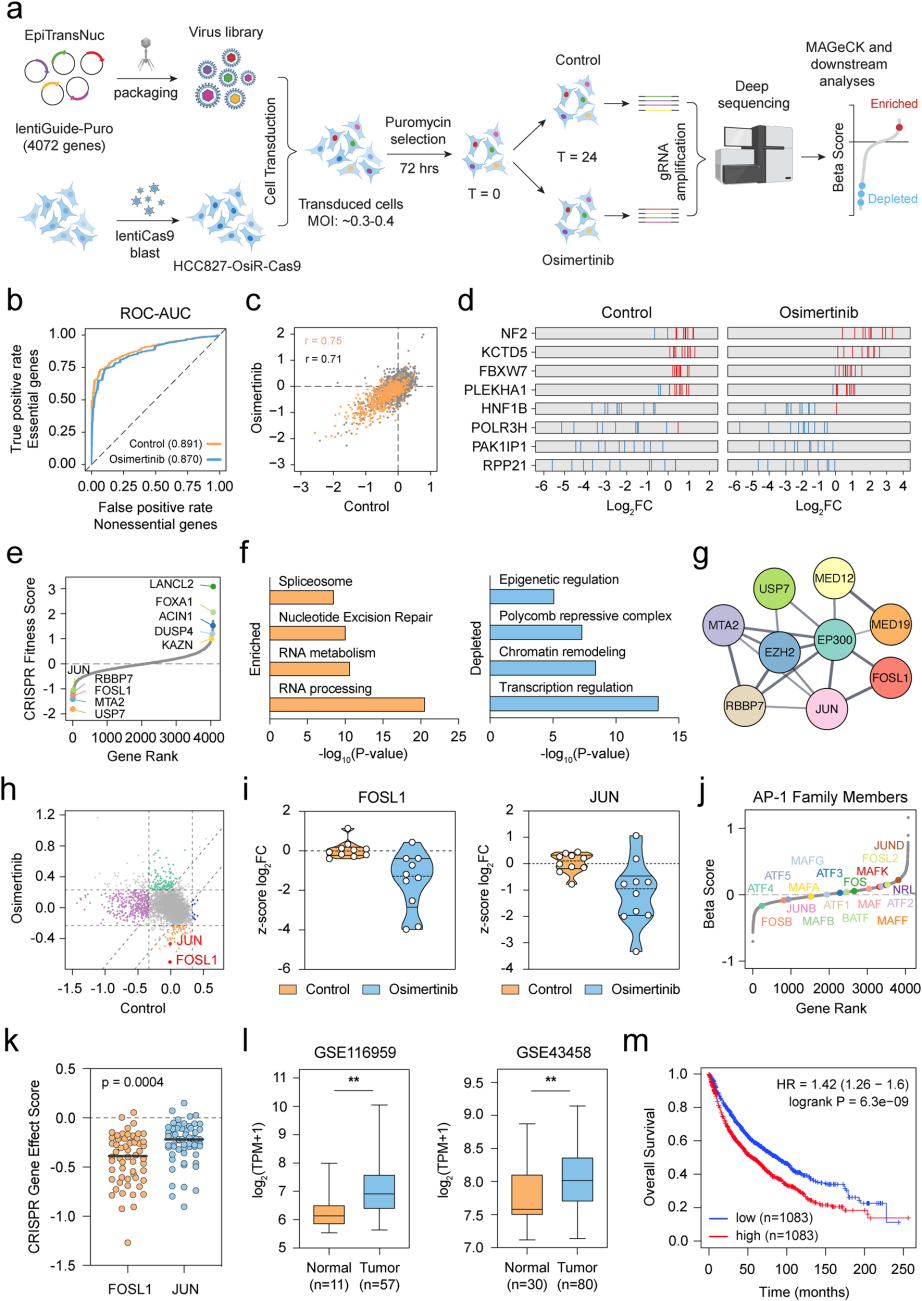
Focused CRISPR screen identifies mediators of osimertinib resistance. **a** Experimental workflow of CRISPR knockout screen performed in Cas9 expressing HCC827-OsiR cells. **b** ROC-AUC analysis of CRISPR screening performance. False positive rates are determined by nonessential genes and plotted against true positive rates, determined by 1580 core essential genes. **c** Correlation between control and osimertinib treatment conditions. Genes from the EpiTransNuc library are shown, with log2 fold change (log_2_FC) values of 1580 core essential genes shown in orange and all other genes in gray [31]. r indicates Pearson’s correlation. **d** Log_2_FC distribution of unique gRNAs for the top and bottom 4 ranked genes under both control and treatment conditions. gRNAs with negative log_2_FC values are represented by blue lines, while those with positive log_2_FC changes are shown in red. **e** Plot showing the distribution of CRISPR fitness scores (normalized *z-scores* for genes in each group, treatment vs control). Examples of enriched (positively scoring) and depleted (negatively scoring) genes are shown. **f** Bar plot showing pathway and GO enrichment analysis in significantly enriched and depleted gene sets. Log-transformed *p*-values are shown by bar plots. **g** StringDB network visualization with Cytoscape depicts molecular interactions of negatively selected hits from CRISPR screen. **h** 9-square plot identifies *FOSL1* and JUN (red dots) as treatment-associated genes. Genes in the purple group are strongly negatively selected in the control group but show only weak changes in selection with treatment. Genes in the green group are weakly selected in the control but strongly positively selected in the treatment group. Genes in the blue group are strongly positively selected in the control and weakly selected in the treatment group. Genes in the orange group are weakly selected in the control and strongly negatively selected in the treatment. These genes may indicate potential synthetic lethal interactions with the drug treatment. The x-axis displays beta-scores in the control condition, while the y-axis shows beta-scores in the treatment condition. **i** Violin plots showing the *z-score* distribution of *FOSL1* and *JUN* gRNAs in control and osimertinib-treated groups. **j** Rank plot illustrating the beta score distribution of AP-1 family members. **k** Plot depicting the CRISPR gene effect score distribution of *FOSL1* and *JUN* genes in lung cancer cell lines (Data is obtained from DepMap, 24Q2 release). **l** GEO datasets showing increased *FOSL1* expression in lung tumor samples compared to normal tissues. ^**^p < 0.01. **m** Kaplan-Meier survival plot (http://kmplot.com/ analysis/) displaying the overall survival analysis for lung cancer patients expressing high levels of *FOSL1* (number of patients, *n* = 1083 in each group). HR, hazard ratio.

To systematically decipher epigenetic and transcriptional mediators of resistance, we conducted a focused CRISPR knockout screen using EpiTransNuc gRNA library, which targets 4072 genes enriched for epigenetic regulators, transcription factors, and nuclear proteins (Supplementary Fig. 7a, b). Maintaining the polyclonal nature of the resistant population, we first engineered HCC827-OsiR cells to stably express Cas9, which was functionally validated by targeting the pan-cancer essential gene *RPA3* (Supplementary Fig. 7c, d). For the CRISPR screening, HCC827-OsiR-Cas9 cells were transduced with the gRNA library at a low multiplicity of infection (MOI ~ 0.3–0.4) and selected with puromycin. Surviving cells were split into two experimental groups—control and osimertinib-treated (20% inhibitory concentration; IC_20_)—and cultured over 24 days (Fig. 3a). Each condition was assayed in triplicate with sufficient gRNA representation (~ 600 × gRNA coverage per replicate).

Deep sequencing of gRNA-specific amplicons, followed by data analysis with established bioinformatics tools [20], revealed robust and comparable screen performance in both conditions, as measured by receiver operating characteristic (ROC)—area under the curve (AUC) and Pearson correlation coefficient metrics against a predefined set of core essential and nonessential genes [31] (Fig. 3b, c). We further confirmed the accuracy of our screens by visualizing the enrichment or depletion patterns of gRNAs targeting top-scoring genes shared across both conditions (Fig. 3d and Supplementary Table 4). Leveraging CRISPR fitness score, we identified several genes that either confer increased resistance or vulnerability when challenged with osimertinib. Positively selected genes included known regulators of EGFR signaling or drug resistance pathways [32–34], whereas negatively selected genes were enriched for chromatin remodeling factors and transcriptional regulators (Fig. 3e). Pathway and gene ontology analysis of significantly depleted genes returned terms related to chromatin remodeling and transcription regulation. In contrast, enriched genes were associated with RNA metabolism and DNA repair, consistent with the nuclear protein-targeting focus of our gRNA pool (Fig. 3f). Furthermore, protein-protein interaction mapping of negatively selected hits provided insights into a functionally enriched gene network that potentially mediates resistance (Fig. 3g). Intriguingly, this network included transcription factors, members of the nucleosome remodeling and deacetylase (NuRD) complex, a component of the polycomb repressive complex 2 (PRC2), mediator complex subunits, and a chromatin modifying deubiquitinating enzyme. Although it was beyond the scope of the present study to dissect the molecular significance of each hit in the network, we did show by competitive cell proliferation assay that genetic ablation of NuRD chromatin remodelers *MTA2* and *RBBP7* resulted in synthetic lethality when combined with EGFR inhibition (Supplementary Fig. 7e, f). This network also identified an actionable target, EZH2 methyltransferase—the catalytic subunit of PRC2 [35], whose pharmacological inhibition resensitized HCC827-OsiR cells to osimertinib (Supplementary Fig. 7g). These data indicate the role of multiple epigenetic and transcriptional regulators in modulating the resistance phenotype.

We next sought to identify high-confidence hits whose knockout most profoundly impacts cell fitness under osimertinib pressure. To achieve this, we applied the “beta score”, a metric analogous to log fold change in differential gene expression analysis [20]. A positive beta score indicates resistance enhancers, while a negative beta score identifies synthetic lethal interactors, providing novel targets for combinatorial treatment. This approach pinpointed two members of the AP-1 transcription factor family: *FOSL1*, the top-scoring hit and *JUN*, ranking among the top 10 hits (Fig. 3h and Supplementary Fig. 7h). The cumulative score distribution of gRNAs targeting *FOSL1* or *JUN* confirmed their specific depletion in the osimertinib-treated group (Fig. 3i and Supplementary Fig. 7i). Importantly, the high-confidence scoring of these factors was independent of their expression levels, which remained largely unchanged between the two states (Supplementary Fig. 7j, k). Of particular relevance here is the fact that other AP-1 family subunits did not exhibit a significant lethal impact on cell fitness, suggesting a unique, context-dependent role for FOSL1 and JUN in mediating osimertinib resistance (Fig. 3j and Supplementary Fig. 7l).

Given the clinical relevance of AP-1 and its druggability by small molecules [36–39], we prioritized FOSL1 and JUN for further studies. To probe the implications of AP-1 in lung cancer, we first examined gene dependency data from CRISPR knockout datasets in the DepMap portal [40]. Lung cancer cell lines displayed heightened sensitivity to *FOSL1* depletion compared to *JUN* knockout (Fig. 3k). We then assessed whether *FOSL1* expression is differentially regulated in lung tumors and holds prognostic value for patient survival. Analysis of Gene Expression Omnibus (GEO) datasets indicated a significant upregulation of *FOSL1* in lung cancer tissues relative to normal samples (Fig. 3l). Most notably, Kaplan-Meier survival analysis of lung cancer cohorts from the Cancer Genome Atlas (TCGA) and GEO [41] demonstrated that elevated *FOSL1* expression correlates with significantly worse overall survival (Fig. 3m). These results suggest that FOSL1 is a key regulator of lung cancer progression and therapy response. This also supports the idea that selectively targeting specific AP-1 family members may be effective in mitigating the oncogenic effects of the complex [38].

### Genetic loss of *FOSL1* or *JUN* restores sensitivity to osimertinib

**Fig. 4:**
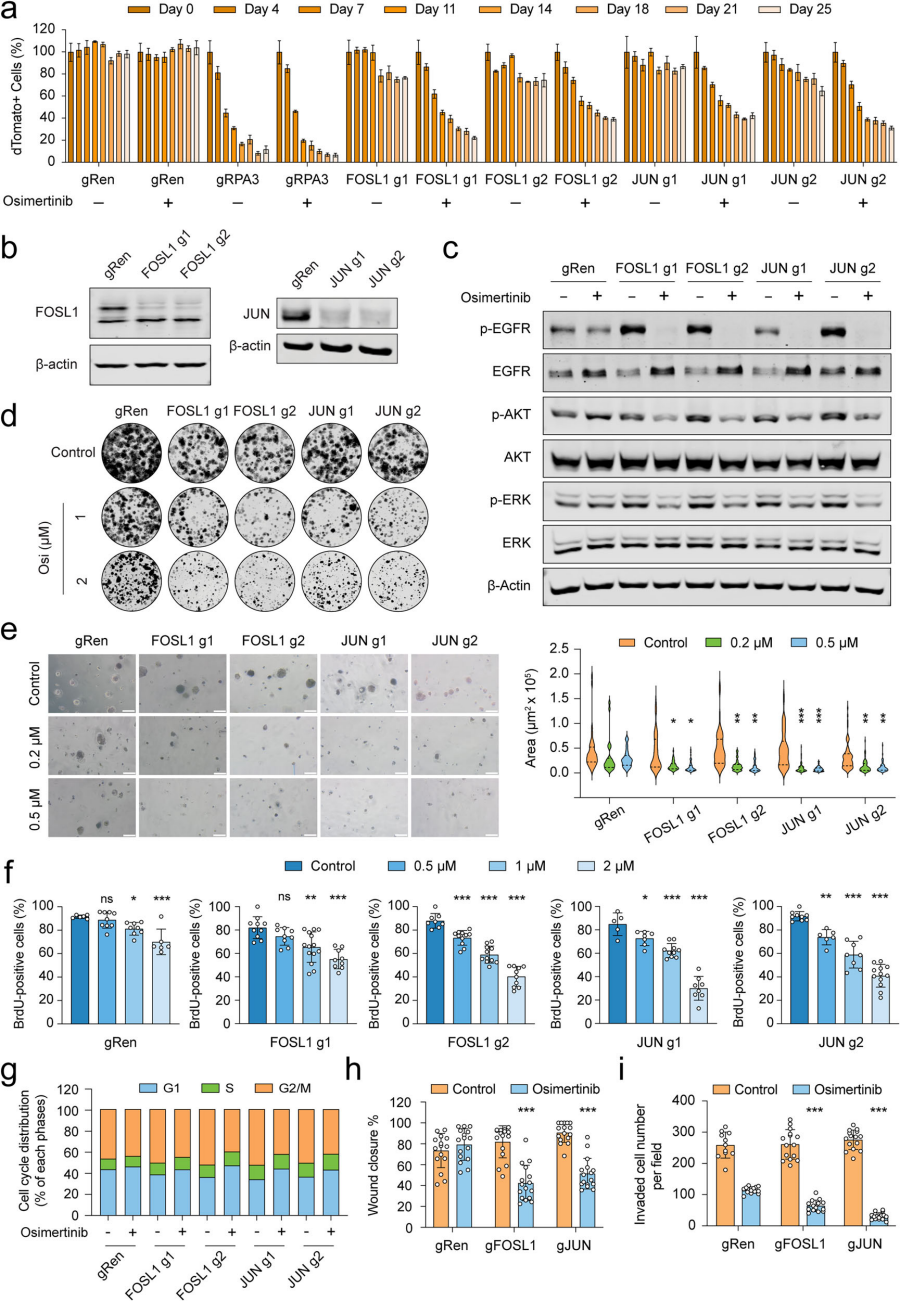
Genetic perturbation of AP-1 reinstates sensitivity to osimertinib. **a** Competitive cell proliferation assay results for HCC827-OsiR-Cas9 cells in the absence (-) or presence (+) of osimertinib (1 µM), transduced with gRNAs targeting Renilla luciferase (gRen, negative control), *RPA3* (positive control), *FOSL1*, and *JUN*. The percentage of dTomato (+) cells at day 0 was set to 100%, and subsequent measurements were normalized accordingly. Data are presented as mean ± SD (*n* = 3). **b** Western blot of *FOSL1* or *JUN* knockout levels in HCC827-OsiR cells. **c** Western blot of EGFR and alternative signaling pathways after 72 h of osimertinib treatment (1 µM) in *FOSL1* or *JUN* depleted HCC827-OsiR cells. β-actin was used as a loading control in western blots. **d** Representative images of crystal violet colony formation assays in *FOSL1* or *JUN* knockout cells treated with osimertinib. **e** Representative images (left) of osimertinib (0.2 μM and 0.5 μM) treatment in *FOSL1* or *JUN* knockout cells in a 3D anchorage-independent soft agar assay (magnification 10x, scale bar: 100 μm) and analysis (right panel) performed on spheroid areas (data range, *n* = 50–82). Significance was calculated using a two-way ANOVA (*n* = 50–82), by comparing each condition to the corresponding condition within the gRen group. *p < 0.05, **p < 0.01, ***p < 0.001. **f** Osimertinib dose-response of BrdU incorporation in *FOSL1* or *JUN* knockout cells. Representative images are provided in Supplementary Fig. 9b. Significance was calculated using a one-way ANOVA and the mean ± SD is shown (*n* = 6–12). *p < 0.05, **p < 0.01, ***p < 0.001. ns, not significant. **g** Osimertinib dose-response analysis (1 µM) of cell cycle phase distribution in *FOSL1* or *JUN* targeted clones. Histograms are represented in Supplementary Fig. 9c. **h** Wound closure analysis in *FOSL1* or *JUN* silenced cells compared to gRen cells, following osimertinib treatment (1 µM), *n* = 16. Representative images are provided in Fig. S9e. **i** Number of invaded cells per field for *FOSL1* or *JUN* knockout clones in the absence or presence of osimertinib (1 µM), *n* = 7–16. Representative images are provided in Supplementary Fig. 9f. Significance for (**h**) and (**i**) was calculated using two-way ANOVA, by comparing each condition to the corresponding condition within the gRen group. Data is shown as the mean ± SD, ***p < 0.001.

To substantiate the role of AP-1 in conferring osimertinib resistance, we conducted an array of cellular and molecular studies. Competitive cell proliferation assays (Supplementary Fig. 7e) confirmed that genetic ablation of AP-1 subunits resensitized HCC827-OsiR cells to osimertinib (Fig. 4a). As expected, knockout of the common essential gene *RPA3* dramatically suppressed cell proliferation in both conditions. Moreover, consistent with the DepMap data (Fig. 3k), loss of *FOSL1* or *JUN* also led to a modest but measurable reduction in proliferative capacity in the absence of drug pressure. We next created knockout clones using gRNAs targeting *FOSL1* or *JUN* (Fig. 4b). In these clones, osimertinib fully abrogated EGFR phosphorylation while significantly blunting activity in bypass signaling pathways (Fig. 4c). Furthermore, long-term 2D and 3D colony formation assays as well as growth on ultra-low adhesion surfaces and hanging drop cell culture revealed that cells lacking *FOSL1* or *JUN* showed heightened sensitivity to osimertinib, especially at higher drug concentrations (Fig. 4d, e, Supplementary Fig. 8a–d). This observation was complemented by an orthogonal approach employing shRNAs to mute the expression of *FOSL1* or *JUN* (Supplementary Fig. 9a).

Further mechanistic insight into the impact of AP-1 depletion on cell proliferation was provided by BrdU incorporation assays, which revealed a marked suppression of DNA synthesis in the knockout clones subjected to osimertinib (Fig. 4f and Supplementary Fig. 9b), accompanied by a cell cycle arrest at G1 phase (Fig. 4g and Supplementary Fig. 9c). Additionally, a slight apoptotic response was observed in AP-1-targeted clones following osimertinib treatment (Supplementary Fig. 9d). Further analyses of phenotypic hallmarks through wound healing and matrigel invasion assays indicated that AP-1 could also modulate functional traits of resistant cells in response to osimertinib (Fig. 4h, i and Supplementary Fig. 9e, f). These results collectively inform that the AP-1 complex is a key regulator of the resistant state, with its genetic depletion inducing conditional lethality and reactivating the anti-tumor effects of EGFR inhibition.

### AP-1 mediates transcriptional control of resistance

**Fig. 5:**
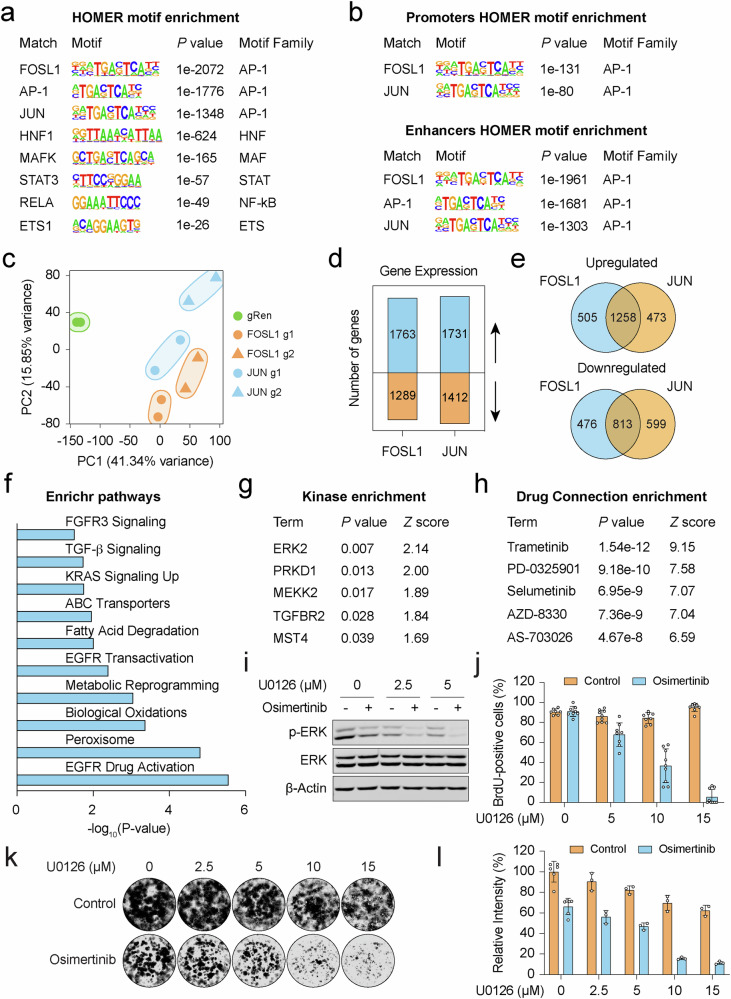
AP-1 modulates resistance-associated gene expression programs. **a** Top enriched motifs from HOMER known motif enrichment analysis of ATAC-seq peaks in the resistant cell state. **b** Enriched motifs identified in promoters and enhancers. **c** PCA plot elucidating variance (%) in transcriptome data of gRen, *FOSL1* or *JUN* targeted clones. **d** Summary of overlapping transcriptome data in *FOSL1* and *JUN* depleted cells. Blue color denotes upregulated genes (up arrow), while orange color represents downregulated genes (down arrow). **e** Venn diagrams showing the intersection of upregulated and downregulated genes in *FOSL1* or *JUN* knockout cells. **f** Bar plot shows pathway enrichment of FOSL1 and JUN target genes, analyzed using Enrichr resource (https://maayanlab.cloud/Enrichr/). **g**, **h** Enrichr-KG enrichment analysis depicting enriched kinome substrates, analyzed using the Kinase Library (**g**) and drug-gene interactions, analyzed using SigCom LINCS Library (LINCS L1000 Chem Pert Consensus Sigs) (**h**) on the same gene set from (**f**). **i** Western blot depicting the ERK-mediated response of HCC827-OsiR cells to combined treatment with osimertinib (1 µM) and U0126. β-Actin was used as a loading control. **j** Proliferation index showing the response of HCC827-OsiR cells to combined treatment with osimertinib (1 µM) and U0126. Representative BrdU incorporation images are provided in Fig. S11c. Data are presented as mean ± SD (*n* = 7–9). **k** Representative images of colony formation assays evaluating the response of HCC827-OsiR cells to combinatorial treatment with osimertinib (1 μM) and U0126. **l** Quantification of 2D colony formation assay. Data are presented as mean ± SD (*n* = 3–6).

Recognizing that AP-1 loss restored sensitivity to osimertinib, we postulated that AP-1 acts as a regulator of transcriptional programs of resistance. To test this tantalizing hypothesis, we turned to the EnrichR tool, which allowed us to infer key mediators of transcriptomic changes in resistant cells. We identified AP-1 (FOSL1 and JUN) as one of the top-ranked transcription factors driving resistance-associated gene expression (Supplementary Fig. 10a). While not all predicted regulators were captured in our CRISPR screens, they possibly influence transcriptional outputs related to the phenotypic and functional hallmarks of HCC827-OsiR cells. To explore chromatin-mediated transcriptional traits, we probed the frequencies of transcription factor binding motifs within differentially accessible ATAC-seq peaks using the Hypergeometric Optimization of Motif Enrichment (HOMER) algorithm. Excitingly, motifs for AP-1 family members were prevalent in resistance-induced chromatin landscape (Fig. 5a and Supplementary Fig. 10b), particularly within promoter and enhancer elements (Fig. 5b). To further dissect the molecular basis of AP-1 activity, we performed bulk RNA-seq analysis in the resistant cell line. Loss of *FOSL1* or *JUN* (two different gRNAs per gene; g1 and g2) led to global changes in gene expression patterns (Fig. 5c, d, Supplementary Fig. 10c), with comparable transcriptional signatures between the two gRNA clones (Supplementary Fig. 10d). Remarkably, the overlap in transcriptomic profiles between *FOSL1* and *JUN* knockouts strongly supported their mutual activity as a dimeric AP-1 complex (Fig. 5e).

To better understand the role of AP-1 activity in controlling transcriptional programs, we sought to integrate transcriptome data of *FOSL1* and *JUN* knockout cells with differential gene expression analysis of resistant versus sensitive states. Notably, AP-1 depletion neutralized differential expression of a set of genes implicated in the regulation of EGFR and FGFR signaling pathways, oxidative stress response, and metabolic reprogramming (Fig. 5f). Consistent with our hypothesis, AP-1 was the most significantly enriched factor in ATAC-seq peaks associated with this gene set (Supplementary Fig. 10e). Enrichment analysis by EnrichR-KG [42] revealed that these genes were clustered within a network of human serine/threonine kinome substrates (Fig. 5g and Supplementary Fig. 11a). Most critically, chemical perturbation signatures identified potential drug-gene interactions, with inhibitors targeting MEK and ERK signaling emerging as potent modulators of this gene network (Fig. 5h and Supplementary Fig. 11b). These results are consistent with our earlier findings that ERK signaling functions as an alternative survival pathway in osimertinib resistant cells. Indeed, pharmacological inhibition of the MEK/ERK pathway reinstated sensitivity to osimertinib in resistant cells, demonstrated by a significant reduction in colony formation and cell proliferation, though the apoptotic response remained somewhat constant (Fig. 5i–l, Supplementary Fig. 11c, d). These data suggest that AP-1 mediates osimertinib resistance primarily by orchestrating transcriptional programs associated with alternative survival pathways, particularly the MEK/ERK signaling axis.

### Pharmacological inhibition of AP-1 restores osimertinib sensitivity

**Fig. 6:**
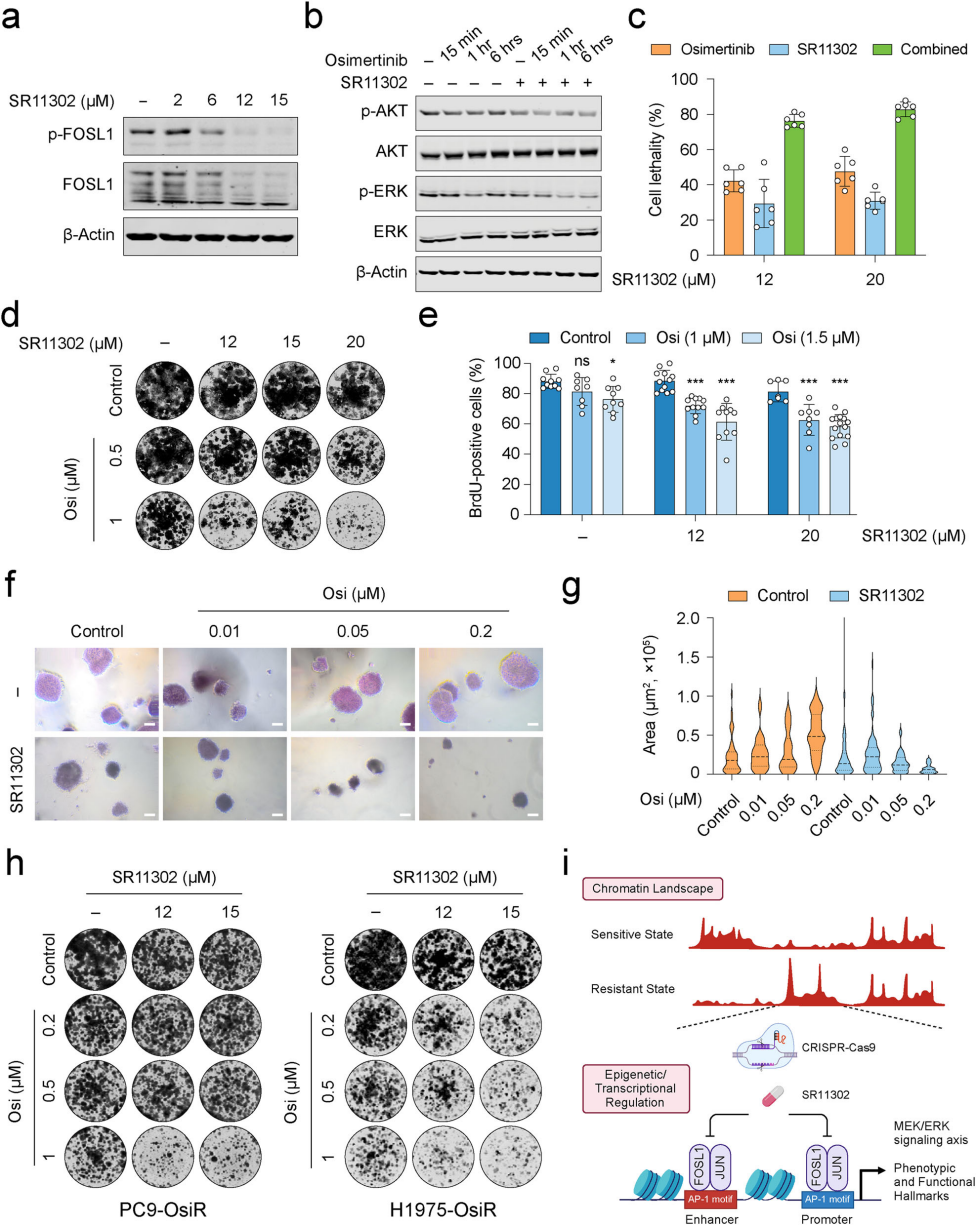
Pharmacological inhibition of AP-1 restores osimertinib responses. **a** Western blot showing dose-dependent destabilization of FOSL1 by SR11302 treatment in HCC827-OsiR cells. **b** Western blot analysis of AKT and ERK signaling pathways illustrating the effects of combinatorial treatment with SR11302 (12 µM) and osimertinib (1.5 µM) in a time-dependent manner. β-actin was used as a loading control in western blots. **c** Cell lethality plot showing the effects of monotherapy or combination of SR11302 and osimertinib (1.5 µM). The data are presented as the mean ± SD (*n* = 6). **d** Representative images of colony formation assays evaluating the response of HCC827-OsiR cells to combinatorial treatment with SR11302 and osimertinib. **e** Proliferation index upon cotreatment of SR11302 and osimertinib. Representative images of DNA labeling with BrdU are provided in Fig. S12d. Significance was calculated using a one-way ANOVA and the mean ± SD is shown (*n* = 6–15). *p < 0.05, ***p < 0.001. ns, not significant. **f** Representative images of 3D anchorage-independent soft agar combined treatment with SR11302 (12 µM) and osimertinib (magnification 10×, scale bar: 100 μm). **g** Quantification of 3D anchorage-independent soft agar data (colony area) for cotreatment with SR11302 (12 µM) and osimertinib (data range, *n* = 31–72). **h** Representative images of colony formation assays evaluating the response of PC9-OsiR and H1975-OsiR cells to combinatorial treatment with varying doses of both SR11302 and osimertinib. **i** Schematic proposal for mechanistic insights into how AP-1 (FOSL1 and JUN) mediates osimertinib resistance. AP-1 governs a gene expression program that regulates MEK/ERK signaling axis and resistance-associated hallmarks. Genetic and pharmacological inhibition of AP-1 activity reinstates drug sensitivity. The image is created with Biorender.com.

To investigate therapeutic implications of conditional lethality involving dual EGFR and AP-1 targeting, we explored the effects of AP-1 pharmacological inhibition. We targeted AP-1 using SR11302, a small molecule retinoid with selective affinity for FOSL1 [39, 43]. Treatment with SR11302 effectively reduced FOSL1 phosphorylation, a critical event in FOSL1 activation [44], and led to a dose-dependent destabilization of FOSL1 in HCC827-OsiR cells (Fig. 6a). Notably, osimertinib treatment alone did not significantly affect FOSL1 phosphorylation (Supplementary Fig. 12a). The suppression of cell growth by osimertinib and SR11302 combination correlated with dose-dependent suppression of AP-1 activity (Supplementary Fig. 12b). Mechanistically, SR11302 enhanced osimertinib-mediated suppression of ERK signaling, with moderate effects on AKT signaling (Fig. 6b). Proliferation and colony formation assays confirmed a synergistic interaction between EGFR and AP-1 inhibition (Fig. 6c, d). It is noteworthy that combining osimertinib with T5224, a c-FOS-selective AP-1 inhibitor [45], did not affect the growth of resistant cells, confirming the specific lethality associated with FOSL1 inhibition (Supplementary Fig. 12c). BrdU incorporation assays further showed that dual inhibition significantly impaired DNA synthesis compared to single-agent treatments (Fig. 6e and Supplementary Fig. 12d). Of additional intrigue, the growth suppressive effects of high-dose AP-1 inhibition were attributed to reduced proliferation and increased apoptosis (Supplementary Fig. 12e). In testing the therapeutic potential of AP-1 inhibition in a tumor-relevant anchorage-independent growth assay, we found that concurrent treatment significantly reduced spheroid formation in resistant cells (Fig. 6f, g). Lastly, to broaden the relevance of our findings, we assessed two additional *EGFR-*mutant NSCLC cell lines with acquired osimertinib resistance, PC9-OsiR and H1975-OsiR (Supplementary Fig. 12f, g). Both genetic silencing and pharmacological inhibition of AP-1 resensitized these cells to osimertinib, with a particularly pronounced effect in H1975-OsiR cells (Fig. 6h, Supplementary Fig. 12h, i). Taken together, these results strengthen the therapeutic potential of targeting the AP-1 transcription factor as a strategy to restore EGFR inhibitor efficacy.

## Discussion

Despite initial benefits in clinical settings, the majority of patients treated with osimertinib eventually develop resistance. Mechanisms of resistance are multifaceted and heterogeneous. While genetic alterations have been extensively cataloged [46], growing evidence highlights the critical role of genome-wide epigenetic reprogramming of regulatory elements in mediating drug resistance [47, 48]. Central to this regulatory epigenome is chromatin accessibility, which rewires transcriptional landscapes by modulating transcription factor occupancy at *cis*-regulatory elements [49, 50].

In this study, we systematically dissect the nexus between chromatin dynamics and transcriptional reprogramming using an osimertinib-resistant NSCLC model replicating key cellular and molecular features of EGFR-TKI-resistant tumors [51–53]. Through multi-omic integration of ATAC-seq and RNA-seq datasets, we define the epigenetic regulome and transcriptomic signatures that characterize the resistant state. Our data reveal robust concordance between altered chromatin accessibility and gene expression reprogramming. For example, chromatin-mediated upregulation of novel or signature vulnerabilities, such as FGF5, FGFR1, and AKT3 supports combinatorial targeting of the FGFR/AKT axis to overcome EGFR-TKI resistance [54]. Furthermore, chromatin remodeling at loci encoding EMT markers correlates with resistance phenotypes, consistent with EMT’s established role in EGFR-TKI tolerance [51]. Our integrative strategy also uncovers resistance-associated long non-coding RNAs and transcript variants, including the truncated isoform VAV3.1. Unlike full-length VAV3 oncogene, which contains N-terminal autoinhibitory domains, VAV3.1 retains only the C-terminal SH3–SH2–SH3 modules, potentially acting as a constitutively active scaffold to amplify oncogenic signaling. As a guanine nucleotide exchange factor, VAV3 activates Rho GTPases and MAPK cascades to drive cytoskeletal remodeling and RTK-mediated bypass signaling [55]. In essence, VAV3.1 exemplifies how chromatin accessibility primes de novo transcriptional programs and implicates isoform switching as a bona fide resistance mechanism in EGFR-TKI-resistant NSCLC or multi-drug resistance [56].

Beyond mapping concordant gene regulatory changes, we exploit a focused CRISPR screen to uncover chromatin remodelers and transcription factors that potentially drive osimertinib resistance. Recent advancements in CRISPR-based screening, enabled by tailored libraries, have accelerated the discovery of epigenetic and transcription factor–dependent mechanisms across diverse contexts [57–60]. Building on these efforts, our library expands the scope of interrogated nuclear regulators, encompassing chromatin remodelers, transcription factors, and other nuclear-associated genes, to enable systematic discovery of functional dependencies across biological and disease models. Our screen identifies unique targets with significant protein-protein interactions, clustering within a specific regulatory network. Notably, MTA2 and RBBP7, which are typically found within the NuRD complex [61] and PRC2 complex [62], play essential roles in chromatin remodeling and gene expression regulation. Of particular interest, EZH2, a histone modifier and core member of PRC2, also emerges as a significantly depleted druggable target in the osimertinib-treated group. Given its role in promoting aggressive tumor fates or therapy resistance [63, 64], our findings suggest that targeting EZH2 may be a viable strategy to combat EGFR-TKI resistance. Supporting this hypothesis, EP300, a general transcriptional coactivator that correlates with EZH2 dependency in solid tumors [65], also appears as a significant hit. Perhaps most strikingly, the screening results highlight components of the AP-1 transcription factor family, specifically FOSL1 and JUN, but none of the other members, as the most prominent hits in the resistance-associated regulatory network. This is particularly noteworthy, as AP-1 has long been recognized for its central role in regulating tumor-associated hallmarks, including the process of EMT [66–68]. Our validation experiments confirm that loss of AP-1 activity resensitizes cells to osimertinib, significantly reducing cell growth and proliferation or impairing resistance phenotypes. In line with our findings, FOSL1 activation has been identified as a prognostic marker in lung adenocarcinomas [69], and biomedical knowledge-based recommendations predict FOSL1 as a potential marker of osimertinib resistance [70].

Our findings indicate that differentially accessible chromatin sites in the drug-resistant state are enriched for AP-1 transcription factor motifs. Intriguingly, recent studies have implicated mSWI/SNF-mediated chromatin remodeling in osimertinib resistance, with the AP-1 family of transcription factors (FOS and JUNB) proposed as regulators of resistance-associated gene expression changes [10]. Notably, mSWI/SNF subunits are not identified as significant hits in our CRISPR screens (data not shown). Instead, our data suggest NuRD and PRC2 complexes as potential modifiers of chromatin structure at these genomic sites, fine-tuning accessibility and binding of AP-1 to its cognate DNA sequence. Gene expression analysis of resistant clones deficient in *FOSL1* or *JUN* highlights extensive transcriptomic changes, with significant overlap in downstream target genes, emphasizing the dimeric activity of these two factors. Notably, genes regulated by AP-1 are primarily linked to RTK signaling and oxidative stress responses, potentially enabling cells to evade osimertinib-induced biological oxidations [10]. Further analysis suggests that overrepresented genes are potential substrates for MEK and ERK signaling kinases, consistent with broader transcriptional responses associated with MEK inhibition. The cooperative interaction between AP-1 and nuclear effectors of the Hippo pathway (YAP/TAZ/TEAD) has been implicated in tumorigenesis and responses to MEK and ERK inhibition in solid cancers [9, 71–74]. This agrees with our findings that the interplay between AP-1 and effectors of bypass signaling cascades may modulate therapeutic sensitivity to combined EGFR/MEK inhibition (Fig. 6i).

Therapeutic targeting of AP-1 transcription factor is actively being explored [75]. SR11302, a synthetic retinoid, is known to block AP-1-dependent gene expression and MEK/ERK signaling [76]. It has shown promise in preclinical models by reducing tumor growth, inhibiting migration, and modulating inflammation [37, 43]. In osimertinib resistant cells, SR11302 effectively blocks FOSL1 phosphorylation and diminishes its stability and expression. The combination of SR11302 with osimertinib leads to significant reductions in colony formation, DNA synthesis, and cell viability. Although T5224 has been reported to bind to the FOSL1/JUN dimer [77], its potency is significantly lower compared to c-FOS [78], which may account for its limited ability to suppress AP-1 activity in our cells. In comparing HCC827-OsiR cells with PC9-OsiR and H1975-OsiR cells, our data reveal that while disruption of AP-1 activity impacts osimertinib responses, the effect is somewhat less pronounced in the latter models. This finding suggests that AP-1 is a common factor of resistance, however, it may not be the primary determinant supporting the resistant state across models.

In conclusion, integrating multi-omics data with high-throughput functional CRISPR genomics of epigenetic regulators and transcription factors accentuates the critical role of epigenetic and transcriptional reprogramming in EGFR-TKI resistance while facilitating the discovery of novel druggable targets, such as the AP-1 transcription factor. A secondary CRISPR screen focused on genes directly regulated by the epigenetic-transcription axis or on downstream targets of AP-1 may help establish a comprehensive “from chromatin to protein” framework of resistance. Ultimately, translating these cues into in vivo models and clinical samples of osimertinib resistant *EGFR*-mutant NSCLC patients will be instrumental in validating novel targets that can potentially have a strong therapeutic index in clinical settings.

## Supplementary information

Supplemental Materials

Uncropped Western Blots

## Data Availability

The authors state that the data underlying the findings of this study are provided in the article and the Supplementary Information. RNA-Seq and ATAC-Seq data for HCC827 vs. HCC827-OsiR, as well as RNA-Seq data for FOSL1 or JUN knockout experiments, have been deposited in the Gene Expression Omnibus (GEO) repository under accession numbers GSE278222, GSE278221, and GSE278409, respectively. Please note that each of these SubSeries is part of the GEO SuperSeries with accession number GSE295823. Additional data are available from the corresponding author upon reasonable request.
