# Correction: Integrative multi-omics identifies AP-1 transcription factor as a targetable mediator of acquired osimertinib resistance in non-small cell lung cancer

**DOI:** 10.1038/s41419-026-08740-y

**Published:** 2026-04-24

**Authors:** Bengisu Dayanc, Sude Eris, Nazife Ege Gulfirat, Gulden Ozden-Yilmaz, Ece Cakiroglu, Ozlem Silan Coskun Deniz, Gökhan Karakülah, Serap Erkek-Ozhan, Serif Senturk

**Affiliations:** 1 Izmir Biomedicine and Genome Center, Izmir, Türkiye; 2 Izmir International Biomedicine and Genome Institute, Dokuz Eylul University, Izmir, Türkiye

**Keywords:** Cancer genomics, Gene silencing, Non-small-cell lung cancer

Correction to: *Cell Death & Disease* 10.1038/s41419-025-07711-z, published online 25 May 2025

After publication, we noted an unintentional error in the final assembly of Figure 4e (left panel). The representative image for the gRen sample treated with osimertinib (0.5 µM) was inadvertently duplicated from the gRen untreated control condition (Control). This correction pertains only to the representative image and does not alter the quantitative analysis in Figure 4e (right panel) or affect the interpretations or conclusions of the study. We apologize for this oversight.

**Original Figure 4** 
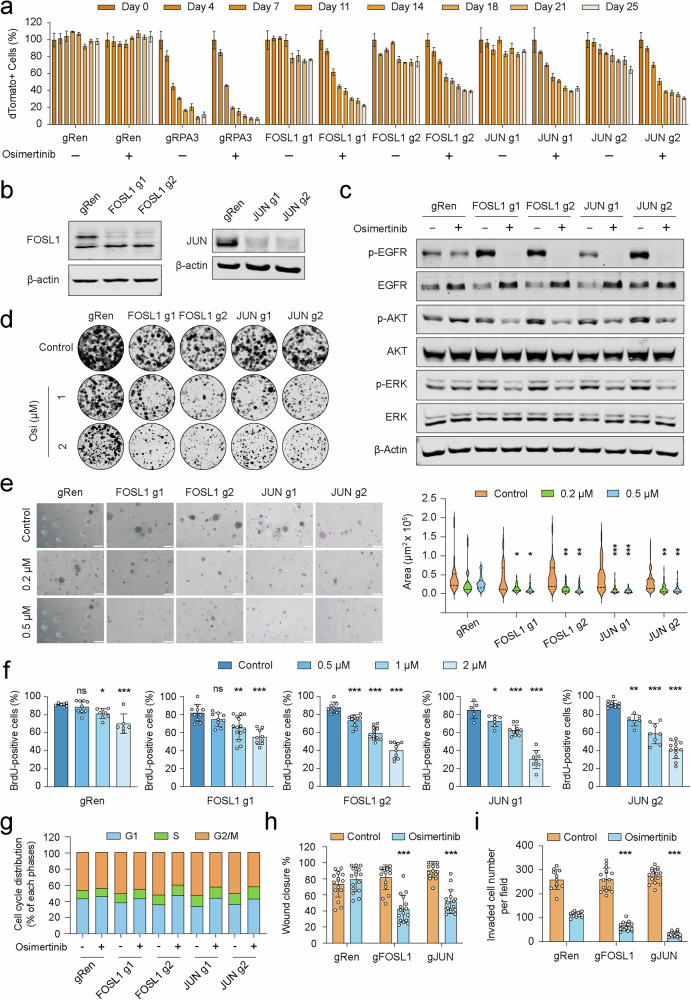


**Amended Figure 4** 
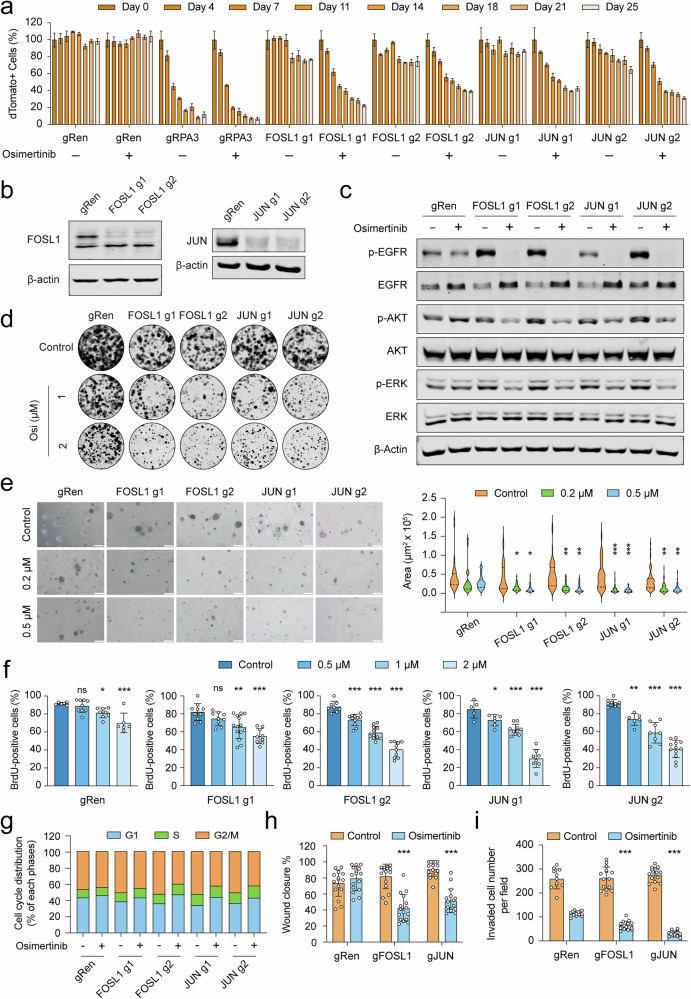


The original article has been corrected.

