# Supplementary material for: Integrative multi-omics identifies AP-1 transcription factor as a targetable mediator of acquired osimertinib resistance in non-small cell lung cancer
Source: Cell Death Dis. 2025 May 25;16(1):414. doi: 10.1038/s41419-025-07711-z (PMC12104440; doi:10.1038/s41419-025-07711-z)
Supplement: Supplementary file 1 — Supplemental Materials [file 41419_2025_7711_MOESM1_ESM.pdf]

Integrative multi-omics identifies AP-1 transcription factor as a targetable mediator of acquired osimertinib resistance in non-small cell lung cancer

Bengisu Dayanc [1,2,*], Sude Eris [1,2,*], Nazife Ege Gulfirat [1,2], Gulden Ozden-Yilmaz [1,2], Ece Cakiroglu [1,2], Ozlem Silan Coskun Deniz [1,2], Gökhan Karakülah [1,2], Serap Erkek-Ozhan [1,2], Serif Senturk [1,2,#]

[1] Izmir Biomedicine and Genome Center, Izmir, Türkiye

[2] Izmir International Biomedicine and Genome Institute, Dokuz Eylul University, Izmir, Türkiye

* These authors contributed equally.

# Correspondence:

Prof. Dr. Serif Senturk

Research Group Leader

Functional Cancer Genomics Group

Izmir Biomedicine and Genome Center

Dokuz Eylul University Health Campus

Mithatpasa St. No: 58/5

35340 Balcova, Izmir / Turkey

Phone 1: 90 (232) 412 65 14

Phone 2: 90 (232) 299 41 61

E-mail: serif.senturk@ibg.edu.tr

http://www.ibg.edu.tr

https://www.senturklab.com/

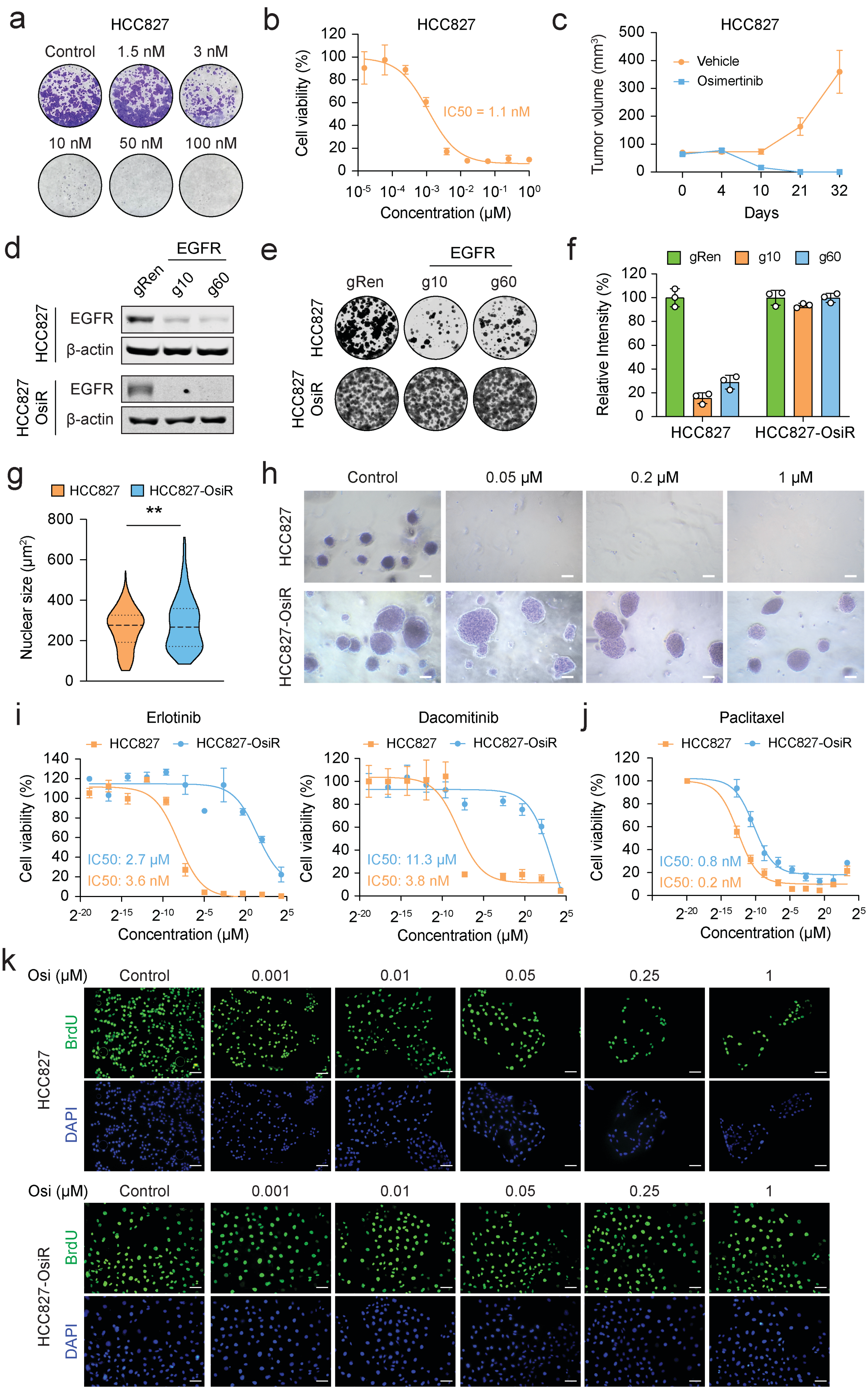

a
HCC827
Control
1.5 nM
3 nM
10 nM
50 nM
100 nM
b
HCC827
Cell viability (%)
IC50 = 1.1 nM
Concentration (µM)
c
HCC827
Tumor volume (mm³)
Vehicle
Osimertinib
Days
d
EGFR
gRen
g10
g60
HCC827
HCC827 OsiR
EGFR
β-actin
e
EGFR
gRen
g10
g60
HCC827
HCC827 OsiR
f
gRen
g10
g60
Relative Intensity (%)
HCC827
HCC827-OsiR
g
HCC827
HCC827-OsiR
**
Nuclear size (µm²)
h
Control
0.05 µM
0.2 µM
1 µM
HCC827
HCC827-OsiR
i
Erlotinib
HCC827
HCC827-OsiR
Cell viability (%)
IC50: 2.7 µM
IC50: 3.6 nM
Concentration (µM)
Dacomitinib
HCC827
HCC827-OsiR
Cell viability (%)
IC50: 11.3 µM
IC50: 3.8 nM
Concentration (µM)
j
Paclitaxel
HCC827
HCC827-OsiR
Cell viability (%)
IC50: 0.8 nM
IC50: 0.2 nM
Concentration (µM)
k
Osi (µM)
Control
0.001
0.01
0.05
0.25
1
HCC827
BrdU
DAPI
Osi (µM)
Control
0.001
0.01
0.05
0.25
1
HCC827-OsiR
BrdU
DAPI

Supplementary Figure 1. Characteristics of osimertinib sensitive and resistant cells. a Representative images of crystal violet colony formation assays conducted in 6-well culture plates (n = 3). **b** Osimertinib dose-response curve for HCC827 cells (n=6). **c** Tumor volume (mm^3) graph of HCC827 xenografts (n=8), untreated or treated with osimertinib (25 mg/kg) via oral gavage daily. **d.** Western blots of EGFR levels in knockout clones (g10 and g60), compared to gRen clone. β-actin served as a loading control. **e** Representative images of crystal violet colony formation assays for EGFR knockout clones, conducted in 6-well culture plates. **f** Quantification of 2D colony formation data of gRen and EGFR (g10 and g60) targeted clones from independent replicates (n=3). **g** Nuclear size (μm^2) quantification based on Lamin A/C staining for HCC827 (n=269) and HCC827-OsiR cells (n=377). Significance was calculated using an unpaired Student's t-test, $^{**}p < 0.01$. **h** Representative images of 3D anchorage-independent soft agar for HCC827 and HCC827-OsiR cells with or without osimertinib treatment (magnification 10×, scale bar: 100 µm). **i** Erlotinib (n=3) and dacomitinib (n=6) dose-response curves comparing IC50 values for HCC827 and HCC827-OsiR cells. **j** Paclitaxel dose-response curves comparing IC50 values for HCC827 and HCC827-OsiR cells (n=6). **k** Representative images of the BrdU incorporation assay for osimertinib dose-response in HCC827 and HCC827-OsiR cells. DAPI is used as a nuclear counterstain (magnification 20×, scale bar: 50 µm)

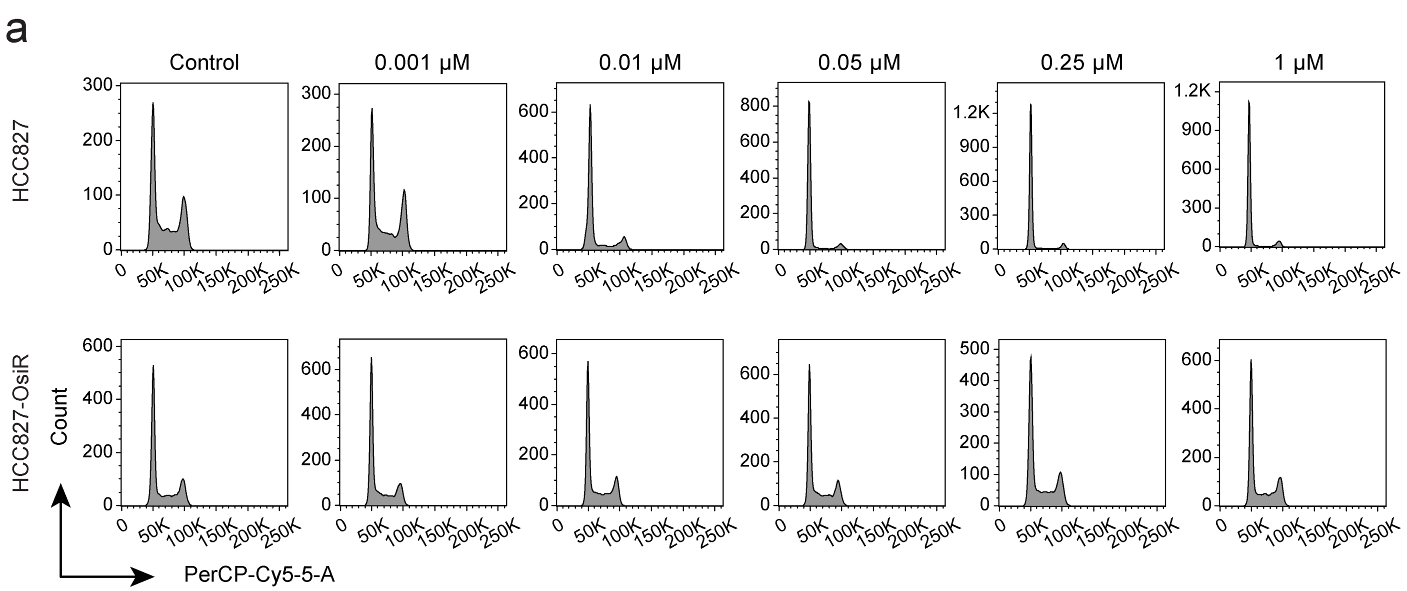

b

HCC827

	Control	0.001 μM	0.01 μM	0.05 μM	0.25 μM	1 μM
G1	40.0±12.7	50.5±9.5	78.1±6.0	87.2±3.5	86.8±5.6	88.7±4.3
S	22.6±4.1	17.3±3.6	8.0±2.0	4.2±1.1	4.6±2.0	3.5±1.5
G2/M	37.2±9.8	32.1±10.1	13.9±4.9	8.6±2.4	8.7±3.6	8.1±3.3

HCC827-OsiR

	Control	0.001 μM	0.01 μM	0.05 μM	0.25 μM	1 μM
G1	49.3±9.9	50.7±9.0	49.1±7.4	50.1±9.3	49.2±9.7	49.8±7.4
S	14.7±7.7	14.9±8.1	15.2±8.1	15.6±8.0	15.3±8.1	14.5±7.8
G2/M	30.6±8.5	29.3±9.1	30.9±8.9	29.7±9.9	30.2±10.4	30.7±7.6

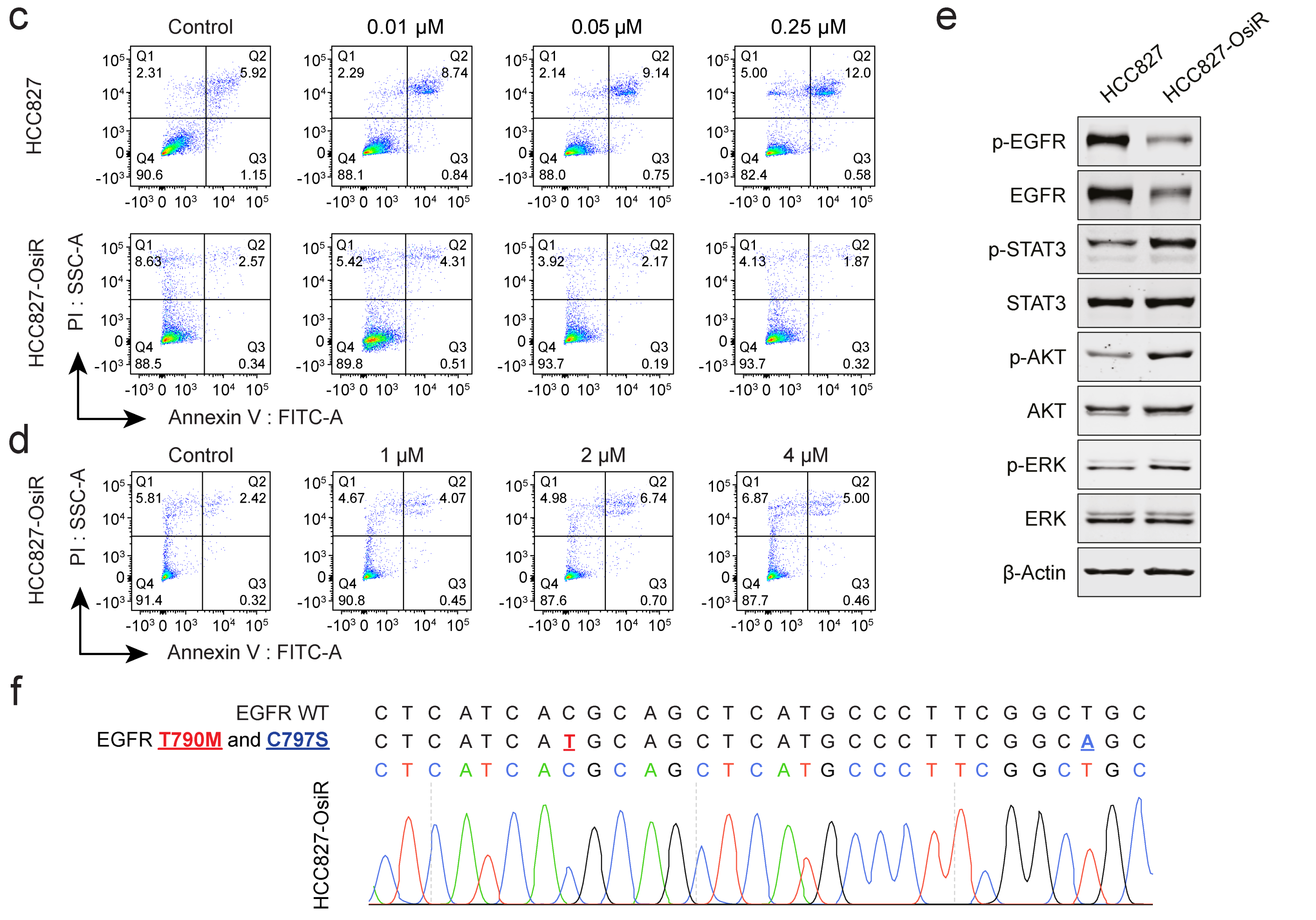

Supplementary Figure 2. Cellular and molecular features of parental and osimertinib resistant cells. **a** Representative histograms for cell cycle distribution in HCC827 and HCC827-OsiR cells, upon exposure to different doses of osimertinib. **b** Tables showing dose-dependent alterations in cell cycle phases in HCC827 and HCC827-OsiR cells. The mean ± SD is shown (n=3). **c** Flow cytometry analysis using an Annexin V/PI apoptosis assay in HCC827 and HCC827-OsiR cells following osimertinib treatment. The right quadrants (Q2 and Q3) show apoptotic cells. **d** Annexin V/PI plots in HCC827-OsiR cells following treatment with higher doses of osimertinib. **e** Western blots of EGFR and alternative signaling pathways in HCC827 and HCC827-OsiR. β-actin was used as a loading control. **f** Sanger sequencing chromatogram showing the absence of detectable T790M or C797S mutations in HCC827-OsiR cells.

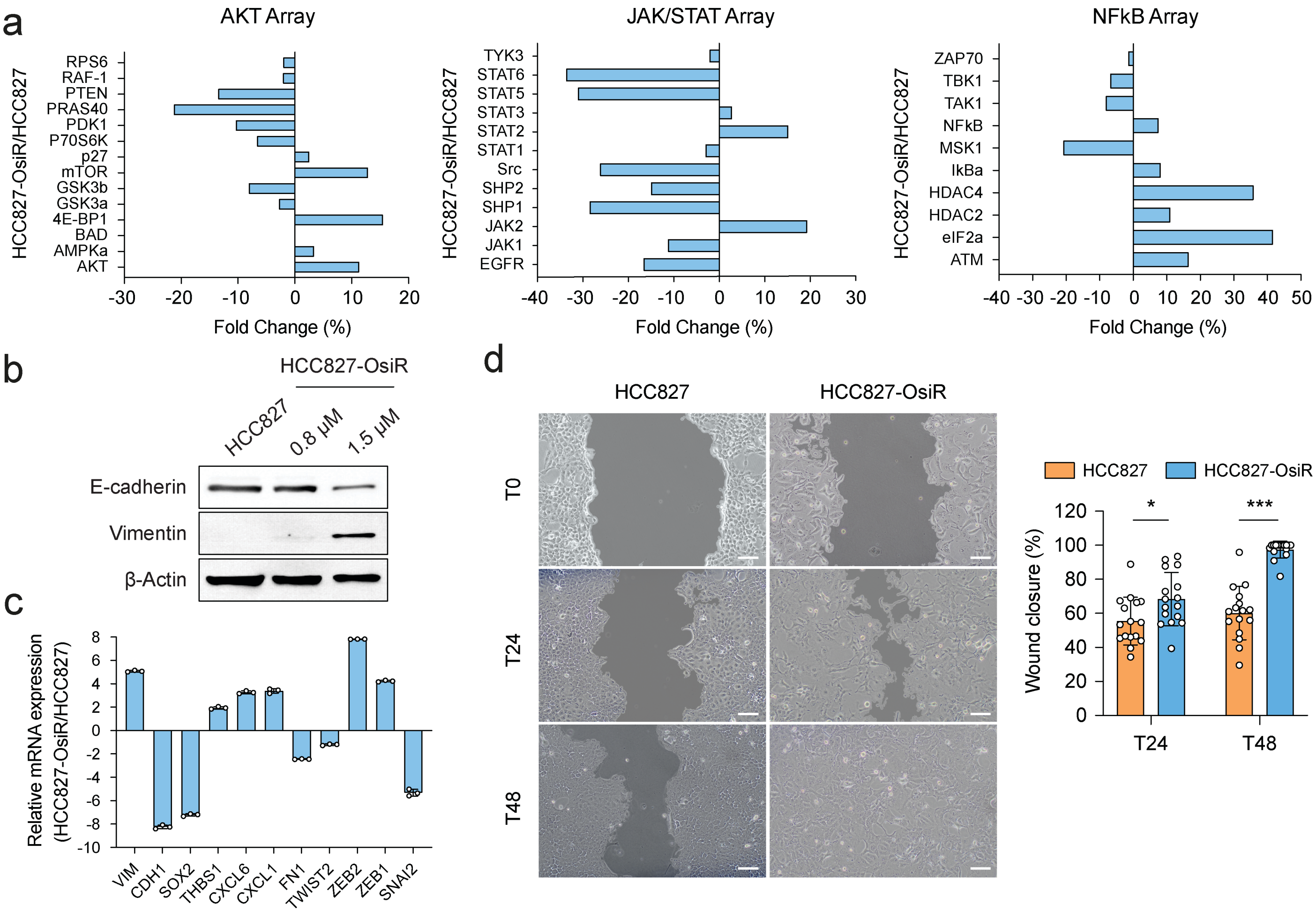
a
AKT Array
HCC827-OsiR/HCC827
RPS6
RAF-1
PTEN
PRAS40
PDK1
P70S6K
p27
mTOR
GSK3b
GSK3a
4E-BP1
BAD
AMPKa
AKT
-30 -20 -10 0 10 20
Fold Change (%)
JAK/STAT Array
TYK3
STAT6
STAT5
STAT3
STAT2
STAT1
Src
SHP2
SHP1
JAK2
JAK1
EGFR
-40 -30 -20 -10 0 10 20 30
Fold Change (%)
NFkB Array
ZAP70
TBK1
TAK1
NFkB
MSK1
IkBa
HDAC4
HDAC2
eIF2a
ATM
-40 -30 -20 -10 0 10 20 30 40 50
Fold Change (%)
b
HCC827-OsiR
HCC827
0.8 μM
1.5 μM
E-cadherin
Vimentin
β-Actin
c
Relative mRNA expression
(HCC827-OsiR/HCC827)
VIM
CDH1
SOX2
THBS1
CXCL6
CXCL1
FN1
TWIST2
ZEB2
ZEB1
SNAI2
d
HCC827
HCC827-OsiR
T0
T24
T48
HCC827
HCC827-OsiR
Wound closure (%)
*
***
T24
T48

Supplementary Figure 3. Alternative signaling pathways and phenotypic hallmarks in parental and osimertinib resistant cells. a Human Phosphorylation Pathway Profiling Array C55 illustrating alterations in intracellular signaling pathways. Fold change difference was calculated based on HCC827-OsiR over HCC827 in AKT, JAK-STAT and NF-kB arrays. **b** Western blot analysis of E-cadherin and Vimentin expression during drug escalation process in HCC827-OsiR cells. β-actin served as a loading control. **c** Quantification of EMT signature genes expression via qRT-PCR in HCC827-OsiR vs HCC827 cells. **d** Representative images for wound healing assay (left) in HCC827 and HCC827-OsiR cells, followed over 48 hours (magnification 10×, scale bar: 100 μm). The right panel shows the percentage of wound closure in both cell lines. Statistical significance was determined using one-way ANOVA, with data presented as the mean ± SD (n=16). *$p<0.05$, ***$p<0.001$.

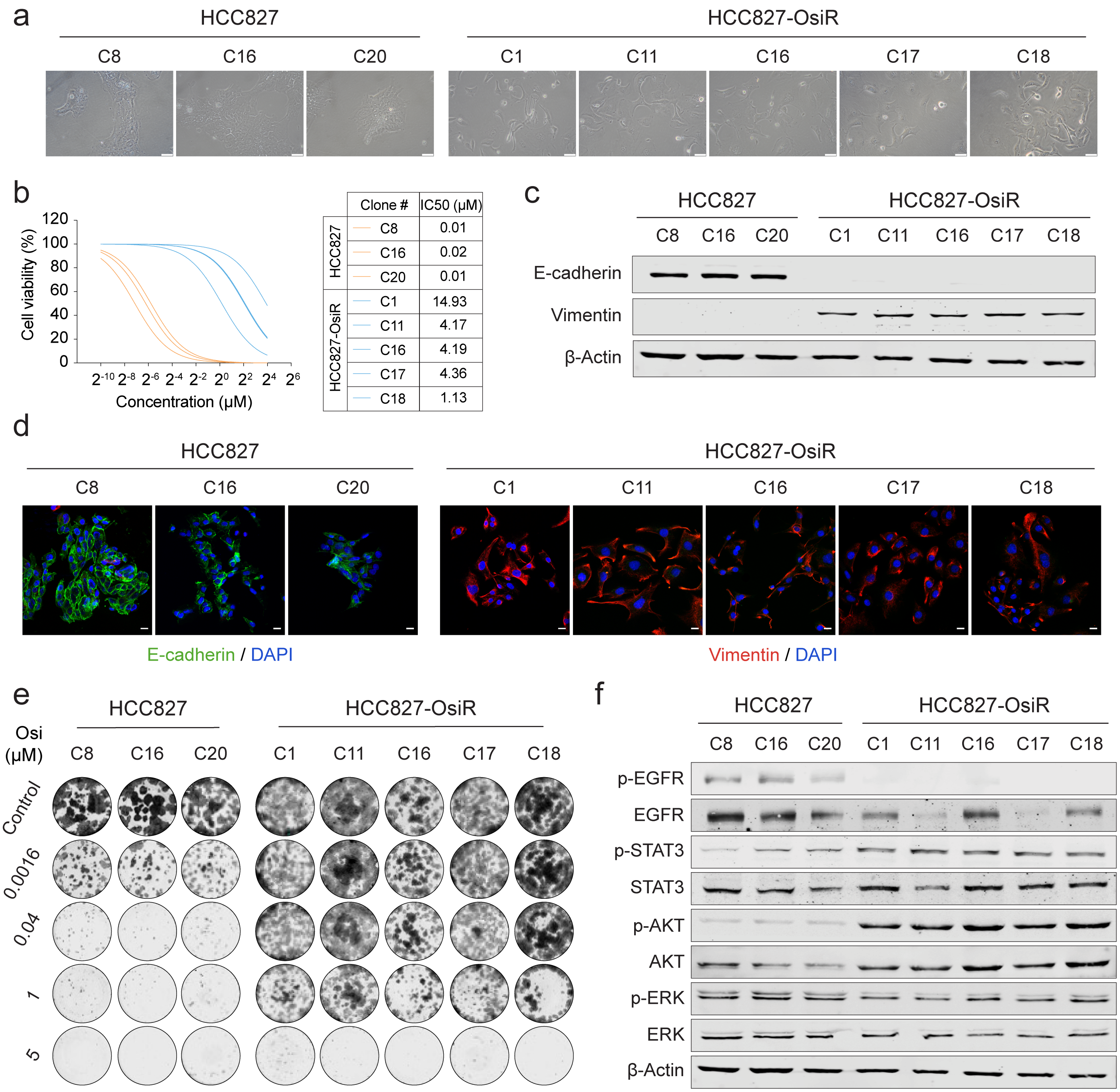
a
HCC827
HCC827-OsiR
C8
C16
C20
C1
C11
C16
C17
C18
b
Cell viability (%)
120
100
80
60
40
20
0
2^{-10} 2^{-8} 2^{-6} 2^{-4} 2^{-2} 2^{0} 2^{2} 2^{4} 2^{6}
Concentration (μM)
Clone # | IC50 (μM)
HCC827
C8 0.01
C16 0.02
C20 0.01
HCC827-OsiR
C1 14.93
C11 4.17
C16 4.19
C17 4.36
C18 1.13
c
HCC827
HCC827-OsiR
C8 C16 C20 C1 C11 C16 C17 C18
E-cadherin
Vimentin
β-Actin
d
HCC827
HCC827-OsiR
C8 C16 C20 C1 C11 C16 C17 C18
E-cadherin / DAPI
Vimentin / DAPI
e
HCC827
HCC827-OsiR
Osi (μM)
C8 C16 C20 C1 C11 C16 C17 C18
Control
0.0016
0.04
1
5
f
HCC827
HCC827-OsiR
C8 C16 C20 C1 C11 C16 C17 C18
p-EGFR
EGFR
p-STAT3
STAT3
p-AKT
AKT
p-ERK
ERK
β-Actin

Supplementary Figure 4. Clonal heterogeneity in parental and osimertinib resistant cells. a Representative images illustrating morphological differences between single cell clones from HCC827 and HCC827-OsiR cells (magnification 20×, scale bar: 50 μm). **b** Osimertinib dose-response curves showing IC50 values for single cell clones derived from HCC827 and HCC827-OsiR cells (n=6). **c** Western blot analysis of E-cadherin and Vimentin expression in HCC827 and HCC827-OsiR single cell clones, with β-actin as a loading control. **d** Immunofluorescence (IF) images depicting the expression of E-cadherin and Vimentin in single cell clones. DAPI serves as a nuclear counterstain (magnification 25×, scale bar: 20 μm). **e** Representative crystal violet colony formation assays comparing osimertinib response among single cell clones (n = 3). **f** Western blot analysis of EGFR and alternative signaling pathways in single cell clones, with β-actin serving as a loading control.

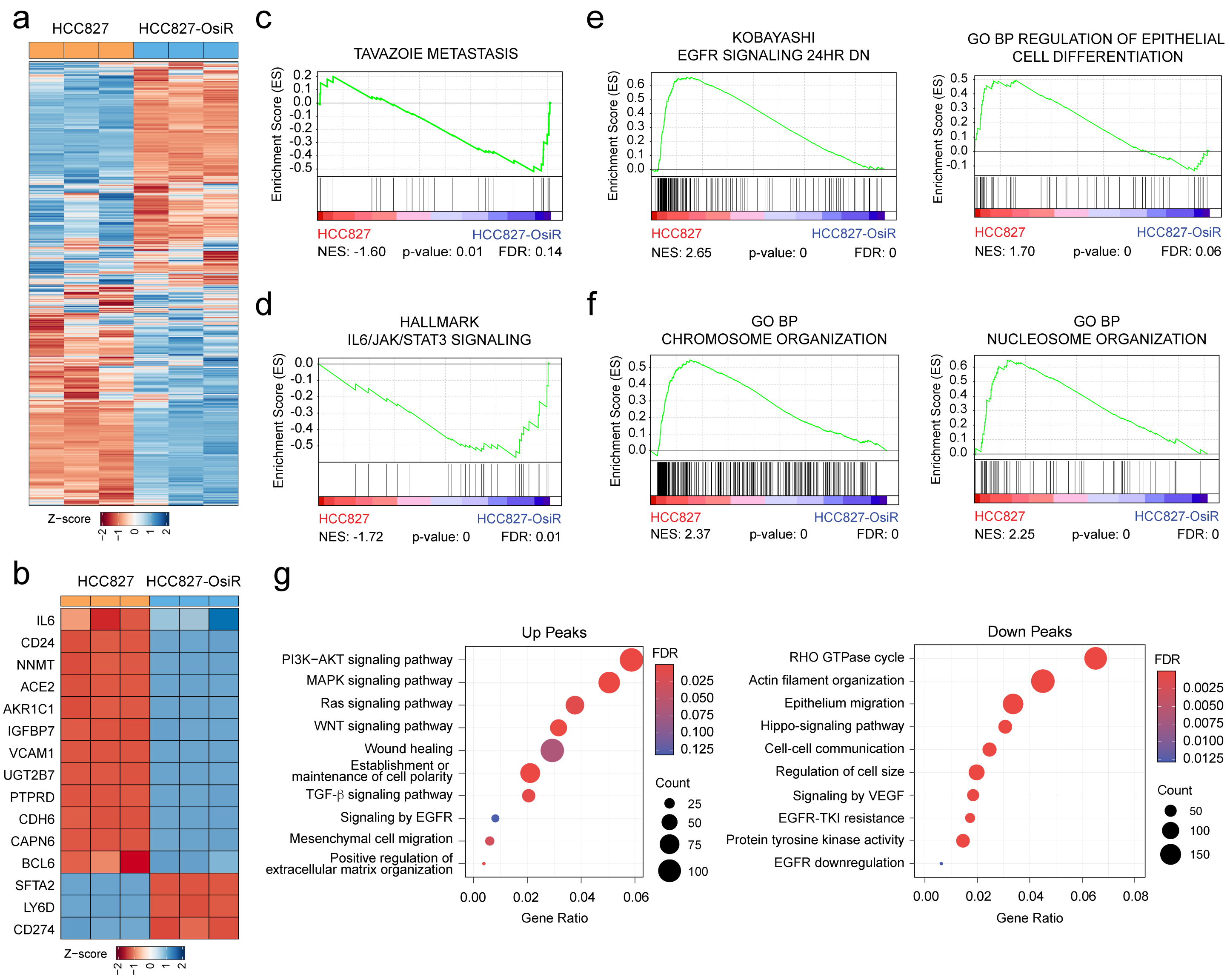
a
HCC827
HCC827-OsiR
Z-score
-2 -1 0 1 2
b
HCC827 HCC827-OsiR
IL6
CD24
NNMT
ACE2
AKR1C1
IGFBP7
VCAM1
UGT2B7
PTPRD
CDH6
CAPN6
BCL6
SFTA2
LY6D
CD274
Z-score
-2 -1 0 1 2
c
TAVAZOIE METASTASIS
Enrichment Score (ES)
HCC827
HCC827-OsiR
NES: -1.60 p-value: 0.01 FDR: 0.14
d
HALLMARK IL6/JAK/STAT3 SIGNALING
Enrichment Score (ES)
HCC827
HCC827-OsiR
NES: -1.72 p-value: 0 FDR: 0.01
e
KOBAYASHI EGFR SIGNALING 24HR DN
Enrichment Score (ES)
HCC827
HCC827-OsiR
NES: 2.65 p-value: 0 FDR: 0
GO BP REGULATION OF EPITHELIAL CELL DIFFERENTIATION
Enrichment Score (ES)
HCC827
HCC827-OsiR
NES: 1.70 p-value: 0 FDR: 0.06
f
GO BP CHROMOSOME ORGANIZATION
Enrichment Score (ES)
HCC827
HCC827-OsiR
NES: 2.37 p-value: 0 FDR: 0
GO BP NUCLEOSOME ORGANIZATION
Enrichment Score (ES)
HCC827
HCC827-OsiR
NES: 2.25 p-value: 0 FDR: 0
g
Up Peaks
PI3K−AKT signaling pathway
MAPK signaling pathway
Ras signaling pathway
WNT signaling pathway
Wound healing
Establishment or maintenance of cell polarity
TGF-β signaling pathway
Signaling by EGFR
Mesenchymal cell migration
Positive regulation of extracellular matrix organization
Gene Ratio
FDR
0.025
0.050
0.075
0.100
0.125
Count
25
50
75
100
Down Peaks
RHO GTPase cycle
Actin filament organization
Epithelium migration
Hippo-signaling pathway
Cell-cell communication
Regulation of cell size
Signaling by VEGF
EGFR-TKI resistance
Protein tyrosine kinase activity
EGFR downregulation
Gene Ratio
FDR
0.0025
0.0050
0.0075
0.0100
0.0125
Count
50
100
150

Supplementary Figure 5. Transcriptomic landscape of parental and osimertinib resistant cells. **a** Whole-genome heatmap of gene expression profiles in HCC827 and HCC827-OsiR samples. **b** Heatmap highlighting differentially expressed genes associated with EGFR-TKI resistance. **c-f** Gene Set Enrichment Analysis (GSEA) mountain plots depicting pathways associated with osimertinib resistance. NES, normalized enrichment score. FDR, false discovery rate. **g** Dot plot showing GO and pathway enrichment analysis of genes with gained or lost chromatin accessibility peaks from ATAC-seq data. FDR, false discovery rate.

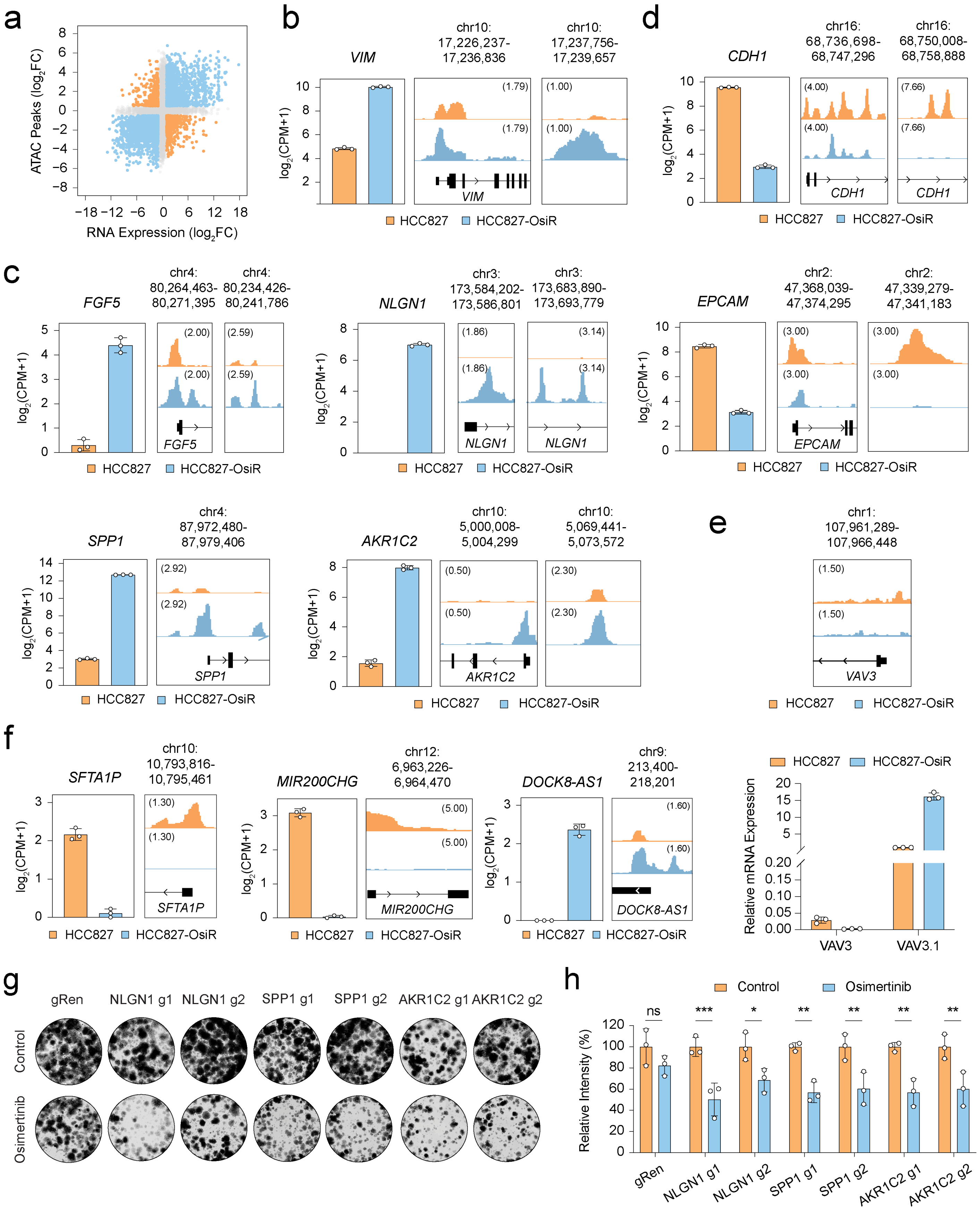

a
ATAC Peaks (log2FC)
RNA Expression (log2FC)
b
VIM
chr10: 17,226,237-17,236,836
chr10: 17,237,756-17,239,657
log2(CPM+1)
HCC827
HCC827-OsiR
d
CDH1
chr16: 68,736,698-68,747,296
chr16: 68,750,008-68,758,888
c
FGF5
chr4: 80,264,463-80,271,395
chr4: 80,234,426-80,241,786
NLGN1
chr3: 173,584,202-173,586,801
chr3: 173,683,890-173,693,779
EPCAM
chr2: 47,368,039-47,374,295
chr2: 47,339,279-47,341,183
SPP1
chr4: 87,972,480-87,979,406
AKR1C2
chr10: 5,000,008-5,004,299
chr10: 5,069,441-5,073,572
e
VAV3
chr1: 107,961,289-107,966,448
f
SFTA1P
chr10: 10,793,816-10,795,461
MIR200CHG
chr12: 6,963,226-6,964,470
DOCK8-AS1
chr9: 213,400-218,201
Relative mRNA Expression
VAV3
VAV3.1
g
gRen
NLGN1 g1
NLGN1 g2
SPP1 g1
SPP1 g2
AKR1C2 g1
AKR1C2 g2
Control
Osimertinib
h
Control
Osimertinib
Relative Intensity (%)
ns
***
*
**

Supplementary Figure 6. Chromatin accessibility profiles and gene regulatory changes associated with osimertinib resistance. a Volcano plots showing the correlation between ATAC-seq peak-associated genes and differentially expressed genes in HCC827-OsiR vs HCC827 cells. **b-d** ATAC-seq tracks over *VIM* (**b**), *FGF5, NLGN1, SPP1*, and *AKR1C2* (**c**), *CDH1* and *EPCAM* (**d**) loci in the HCC827 and HCC827-OsiR cell line pairs. Gene expression $\log_2$(CPM+1) values are displayed in bar graphs. Error bars represent the mean ± SD of the expression level for each cell line. **e** ATAC-seq tracks over *VAV3.1* loci in HCC827 and HCC827-OsiR cells (upper panel), with qRT-PCR quantification of *VAV3* and *VAV3.1* expression levels in both cell lines (lower panel). **f** ATAC-seq tracks over *SFTA1P, MIR200CHG* and *DOCK8-AS1* loci in HCC827 and HCC827-OsiR cell line pairs. Gene expression $\log_2$(CPM+1) values are displayed in bar graphs. Error bars represent the mean ± SD of the expression level for each cell line. **g** Representative images of crystal violet colony formation assays for *NLGN1*, *SPP1* and *AKR1C2* knockout clones, treated with or without osimertinib (1 μM). **h** Quantification of 2D colony formation data of gRen, *NLGN1*, *SPP1* and *AKR1C2* targeted clones. Each control is normalized to 100. Significance was calculated using one-way ANOVA and the mean ± SD is shown (n=3). *$p<0.05$, **$p<0.01$, ***$p<0.001$. ns, not significant.

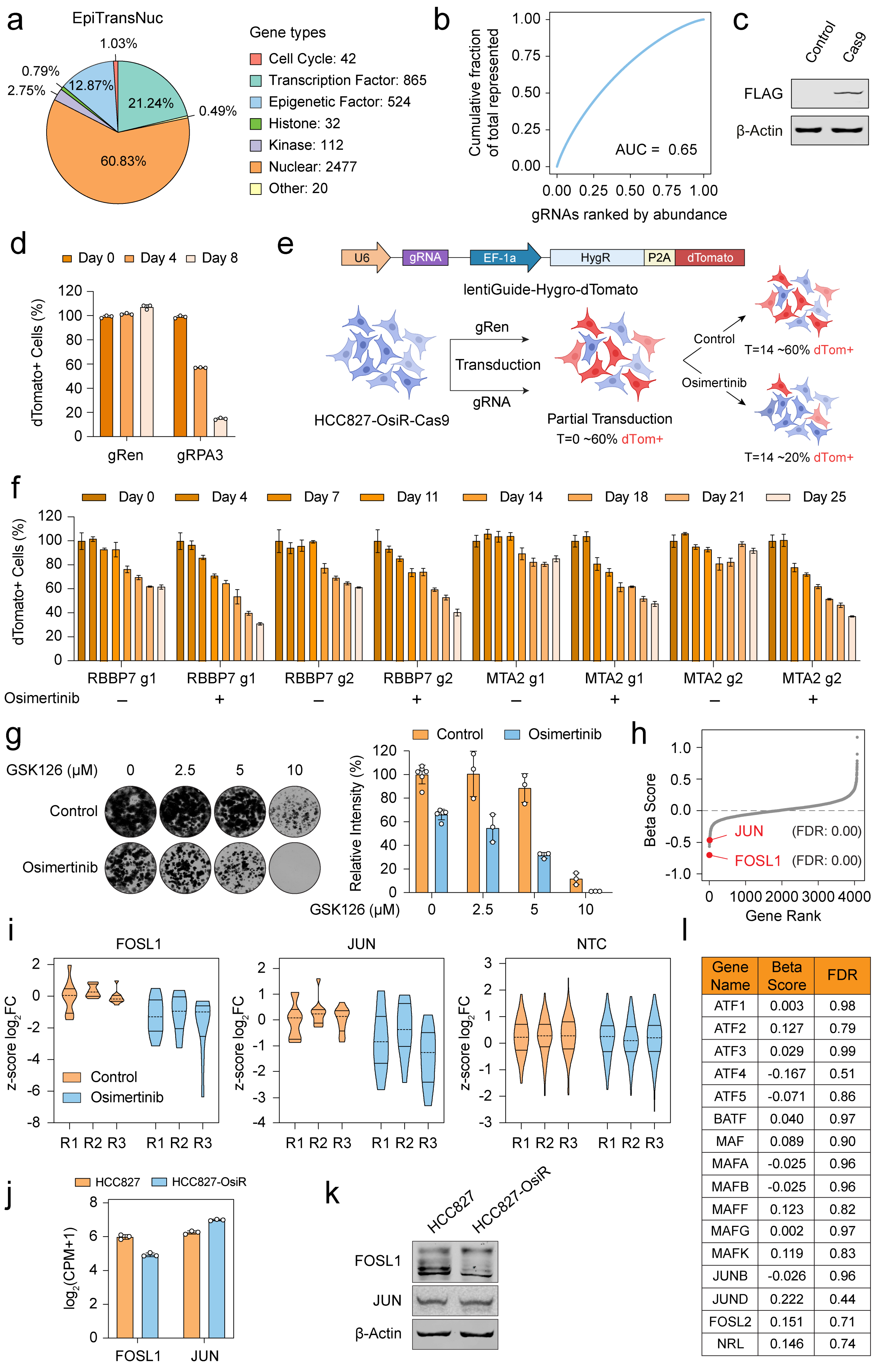

Gene Name	Beta Score	FDR
ATF1	0.003	0.98
ATF2	0.127	0.79
ATF3	0.029	0.99
ATF4	-0.167	0.51
ATF5	-0.071	0.86
BATF	0.040	0.97
MAF	0.089	0.90
MAFA	-0.025	0.96
MAFB	-0.025	0.96
MAFF	0.123	0.82
MAFG	0.002	0.97
MAFK	0.119	0.83
JUNB	-0.026	0.96
JUND	0.222	0.44
FOSL2	0.151	0.71
NRL	0.146	0.74

Supplementary Figure 7. Identification of osimertinib resistance genes by focused CRISPR screen. **a** Composition of the EpiTransNuc library. Percent distribution and number of genes targeted by the library. **b** Lorenz curve illustrating the gRNA representation in the EpiTransNuc library. AUC, area under the curve. **c** Western blot analysis of Cas9 protein expression in HCC827-OsiR-Cas9 cells compared to HCC827-OsiR (control) cells. Flag-M2 antibody was used to detect Cas9. β-actin was used as a loading control. **d** Competitive cell proliferation assay validating functional activity of Cas9. gRPA3 (Replication Protein A3) was used as a positive control. The percentage of tomato-positive cells at Day 0 was normalized to 100%, and the following measurements were calculated accordingly. Bar graphs are presented as the mean ± SD (n = 3). **e** Schematic representation of lentiGuide-Hygro-dTomato backbone and a representative workflow of competitive cell proliferation assay. Red cells represent dTomato+ infected population. **f**. Competitive cell proliferation assay results in the absence (-) and presence (+) of osimertinib (1 μM). Cells were transduced with gRNAs targeting *RBBP7* and *MTA2* genes. Data is presented as mean ± SD (n = 3). **g** Representative images (left) of crystal violet colony formation assay showing the response of HCC827-OsiR cells to increasing doses of GSK126 in combination with osimertinib (1 μM) and quantification (right panel) of 2D colony formation assay (n = 3-6). **h** Rank plot showing beta-score distribution of *FOSL1* and *JUN*. **i** Violin plot depicting the distribution of *z-score* transformed log_2FC for *FOSL1* and *JUN* gRNAs, as well as non-targeting control gRNAs, in both control and osimertinib-treated replicates (R). **j** Transcript levels of *FOSL1* and *JUN* genes, shown as log2(CPM + 1) values in the bar graph. **k** Western blot showing basal protein levels of FOSL1 and JUN in HCC827 and HCC827-OsiR cells. **l** Beta-scores and FDR values of AP-1 family members. *p<0.05, **p<0.01, ***p<0.001. ns, not significant.

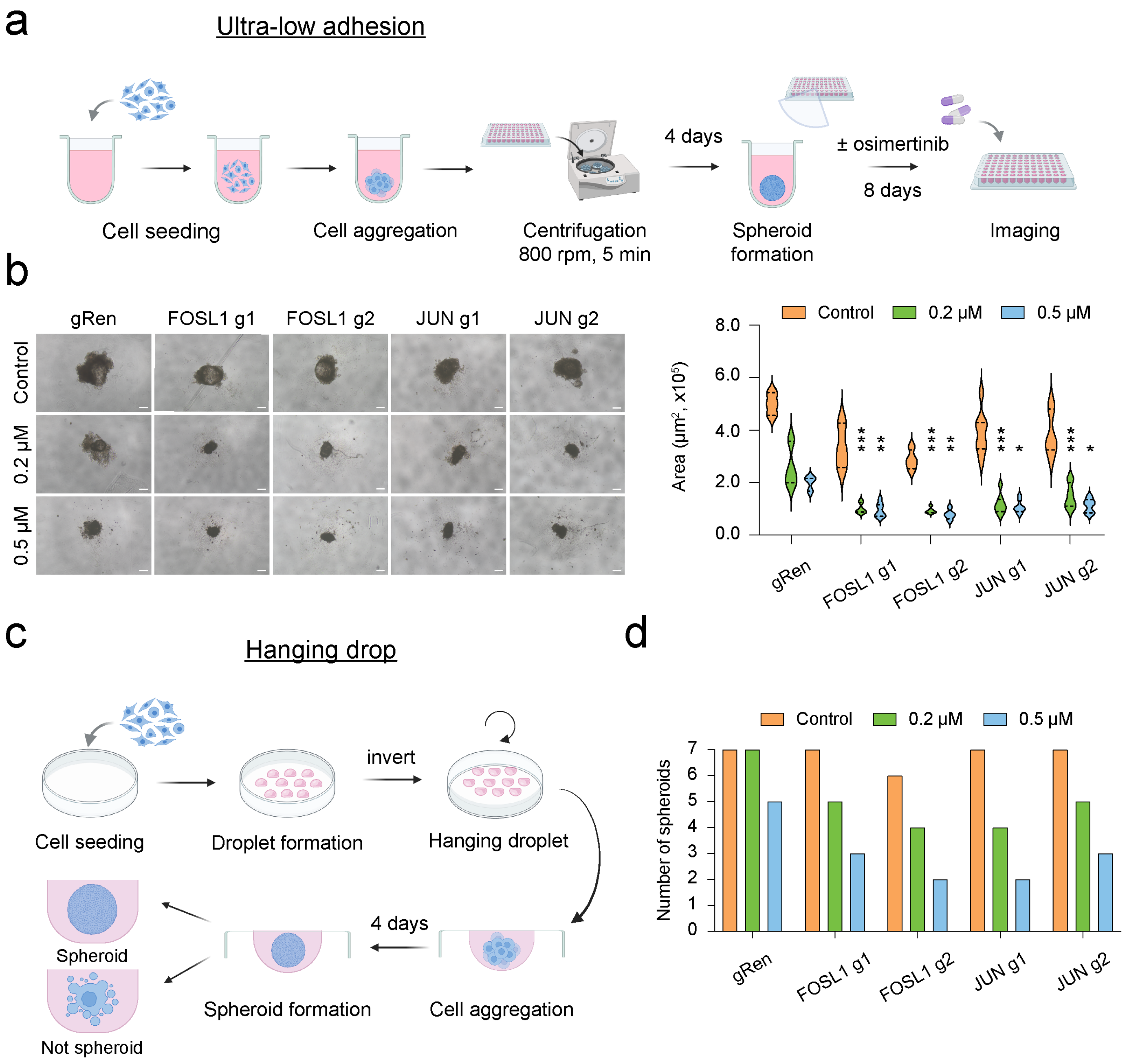

a
Ultra-low adhesion
Cell seeding
Cell aggregation
Centrifugation
800 rpm, 5 min
4 days
Spheroid
formation
± osimertinib
8 days
Imaging
b
gRen
FOSL1 g1
FOSL1 g2
JUN g1
JUN g2
Control
0.2 µM
0.5 µM
Area (µm², x10⁵)
8.0
6.0
4.0
2.0
0.0
c
Hanging drop
Cell seeding
Droplet formation
invert
Hanging droplet
Spheroid
Not spheroid
4 days
Spheroid formation
Cell aggregation
d
Number of spheroids

Supplementary Figure 8. Depletion of *FOSL1* or *JUN* disrupts 3D spheroid formation and growth. **a** Schematic representation of the ultra-low adhesion spheroid assay. **b** Representative images for ultra-low adhesion spheroid assay (left) *FOSL1* or *JUN* knockout cells following osimertinib (0.2 µM and 0.5 µM) treatment (magnification 4×, scale bar: 200 µm). The right panel shows the spheroid size (area, μm^2) in *FOSL1* or *JUN* knockout cells. Statistical significance was determined using two-way ANOVA, comparing each group to the corresponding condition within the gRen group (n=3-8). *$p<0.05$, **$p<0.01$, ***$p<0.001$. **c** Schematic representation of the hanging drop assay. **d** Analysis performed on hanging drop assay based on spheroid counts following osimertinib (0.2 µM and 0.5 µM) treatment in *FOSL1* or *JUN* knockout cells (n=7).

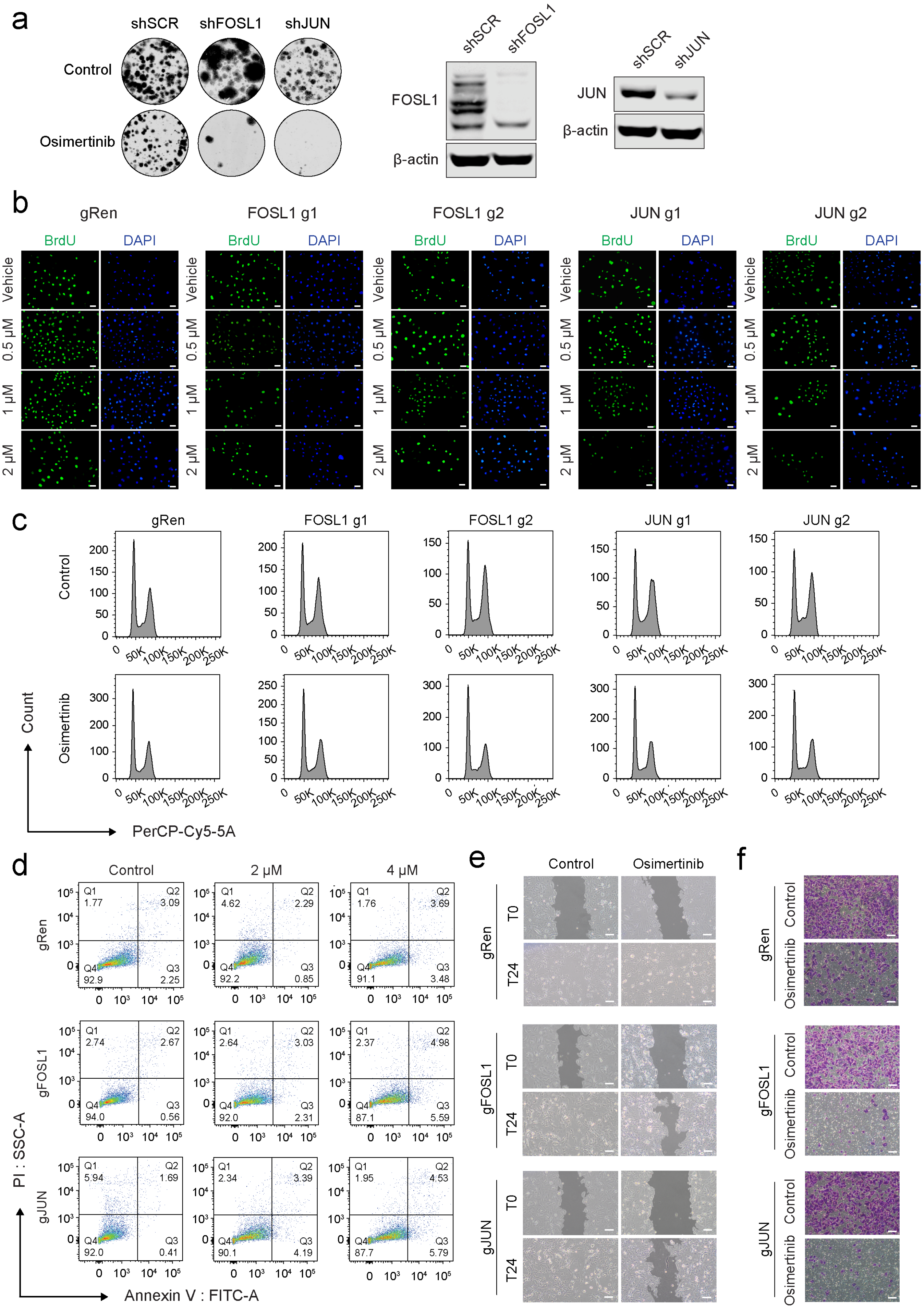
a
shSCR
shFOSL1
shJUN
Control
Osimertinib
shSCR
shFOSL1
FOSL1
β-actin
shSCR
shJUN
JUN
β-actin
b
gRen
FOSL1 g1
FOSL1 g2
JUN g1
JUN g2
BrdU
DAPI
Vehicle
0.5 μM
1 μM
2 μM
c
gRen
FOSL1 g1
FOSL1 g2
JUN g1
JUN g2
Control
Osimertinib
Count
PerCP-Cy5-5A
0
50K
100K
150K
200K
250K
d
Control
2 μM
4 μM
gRen
gFOSL1
gJUN
Q1 1.77
Q2 3.09
Q4 92.9
Q3 2.25
Q1 4.62
Q2 2.29
Q4 92.2
Q3 0.85
Q1 1.76
Q2 3.69
Q4 91.1
Q3 3.48
Q1 2.74
Q2 2.67
Q4 94.0
Q3 0.56
Q1 2.64
Q2 3.03
Q4 92.0
Q3 2.31
Q1 2.37
Q2 4.98
Q4 87.1
Q3 5.59
Q1 5.94
Q2 1.69
Q4 92.0
Q3 0.41
Q1 2.34
Q2 3.39
Q4 90.1
Q3 4.19
Q1 1.95
Q2 4.53
Q4 87.7
Q3 5.79
PI : SSC-A
Annexin V : FITC-A
e
Control
Osimertinib
gRen
gFOSL1
gJUN
T0
T24
f
gRen
gFOSL1
gJUN
Control
Osimertinib

Supplementary Figure 9. Depletion of *FOSL1* or *JUN* restores sensitivity to osimertinib in HCC827-OsiR cells. a Representative images from the crystal violet colony formation assay (left) following osimertinib treatment (1 μM) in *FOSL1* or *JUN* knockdown in HCC827-OsiR cells (n=3). Western blot analysis of FOSL1 and JUN expression in respective knockdown clones (right panel). **b** Representative images of BrdU incorporation for osimertinib dose-response. DAPI is used as a nuclear counterstain (magnification 20×, scale bar: 50 μm). **c** Cell cycle histograms of *FOSL1* or *JUN* knockout clones, osimertinib: 1 μM. **d** Annexin V/PI flow cytometry plots following high-dose osimertinib treatment. Apoptotic cells are shown in the right quadrants (Q2 and Q3). **e** Representative images of wound healing assay in *FOSL1* or *JUN* knockout cells, compared to the gRen control, osimertinib: 1 μM. (magnification: 10×, scale bar: 100 μm). **f** Representative images from the invasion assay, osimertinib: 1 μM (magnification: 10×, scale bar: 100 μm).

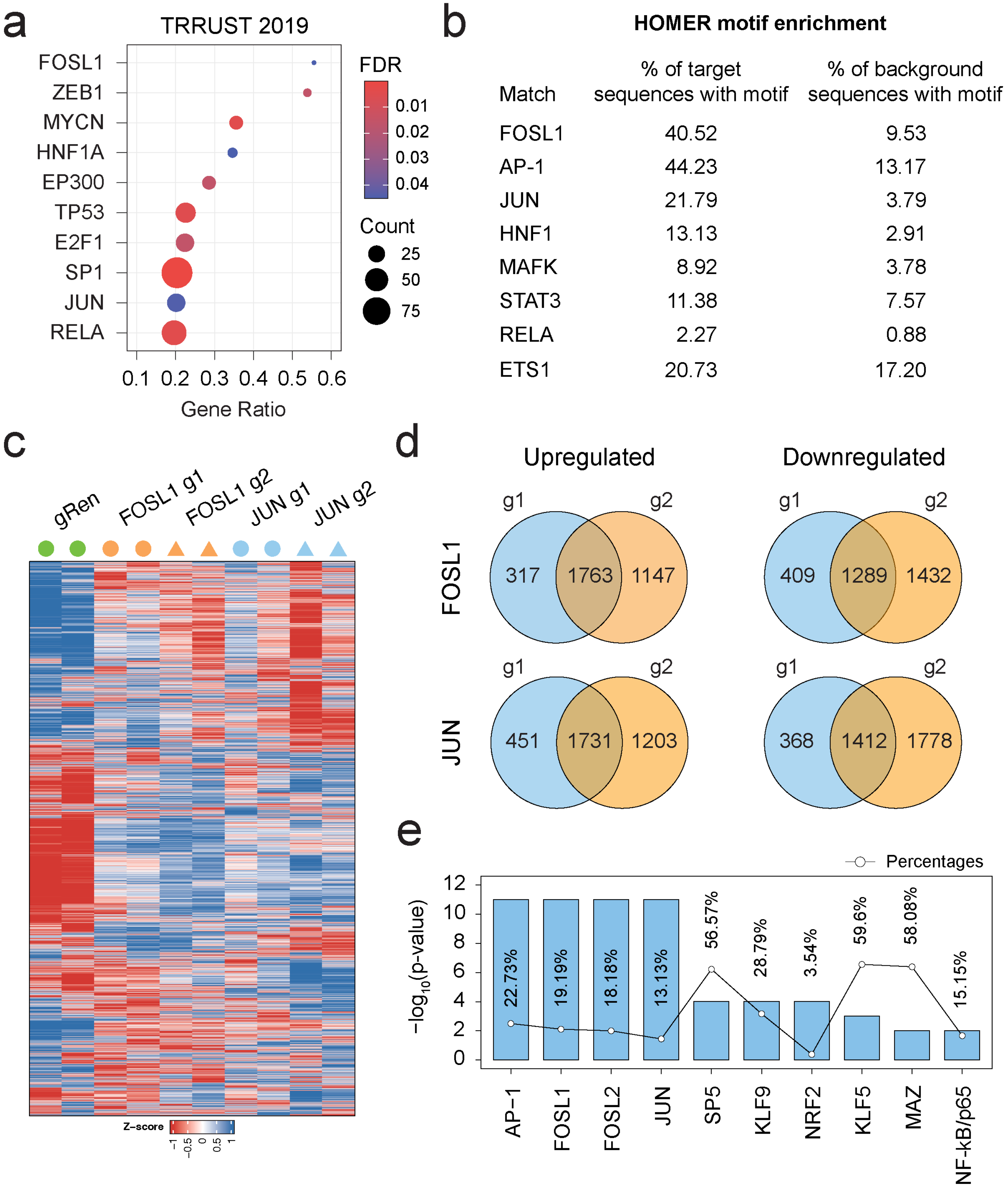
a
TRRUST 2019
FOSL1
ZEB1
MYCN
HNF1A
EP300
TP53
E2F1
SP1
JUN
RELA
0.1 0.2 0.3 0.4 0.5 0.6
Gene Ratio
FDR
0.01
0.02
0.03
0.04
Count
25
50
75
b
HOMER motif enrichment
Match
% of target sequences with motif
% of background sequences with motif
FOSL1 40.52 9.53
AP-1 44.23 13.17
JUN 21.79 3.79
HNF1 13.13 2.91
MAFK 8.92 3.78
STAT3 11.38 7.57
RELA 2.27 0.88
ETS1 20.73 17.20
c
gRen
FOSL1 g1
FOSL1 g2
JUN g1
JUN g2
Z-score
−1 −0.5 0 0.5 1
d
Upregulated
Downregulated
FOSL1
JUN
g1
g2
317 1763 1147
409 1289 1432
451 1731 1203
368 1412 1778
e
Percentages
−log10(p-value)
0 2 4 6 8 10 12
22.73%
19.19%
18.18%
13.13%
56.57%
28.79%
3.54%
59.6%
58.08%
15.15%
AP−1
FOSL1
FOSL2
JUN
SP5
KLF9
NRF2
KLF5
MAZ
NF-kB/p65

Supplementary Figure 10. AP-1 governs resistance-associated gene expression programs. a Transcription factor enrichment analysis of genes with an absolute log2FC > 1.5 and a p-value < 0.05 in HCC827-OsiR vs HCC827 RNA-seq, performed using TRRUST v2 (2019) (https://www.grnpedia.org/trrust/). **b** Top enriched motifs identified through HOMER motif analysis of gained chromatin accessibility peaks in the resistant state, with motif frequencies (%) shown for target and background sequences. **c** Whole-genome heatmap of gene expression profiles across gRen, *FOSL1* or *JUN* deleted samples (duplicate). **d** Venn diagram showing the overlap of upregulated and downregulated genes between two unique gRNAs targeting *FOSL1* or *JUN* genes. **e** Plot illustrating the enrichment of HOMER motifs (bars) and their percentages (line) in genes upregulated in the resistant state and differentially expressed in *FOSL1* and *JUN* knockout (overlap) RNA-seq data.

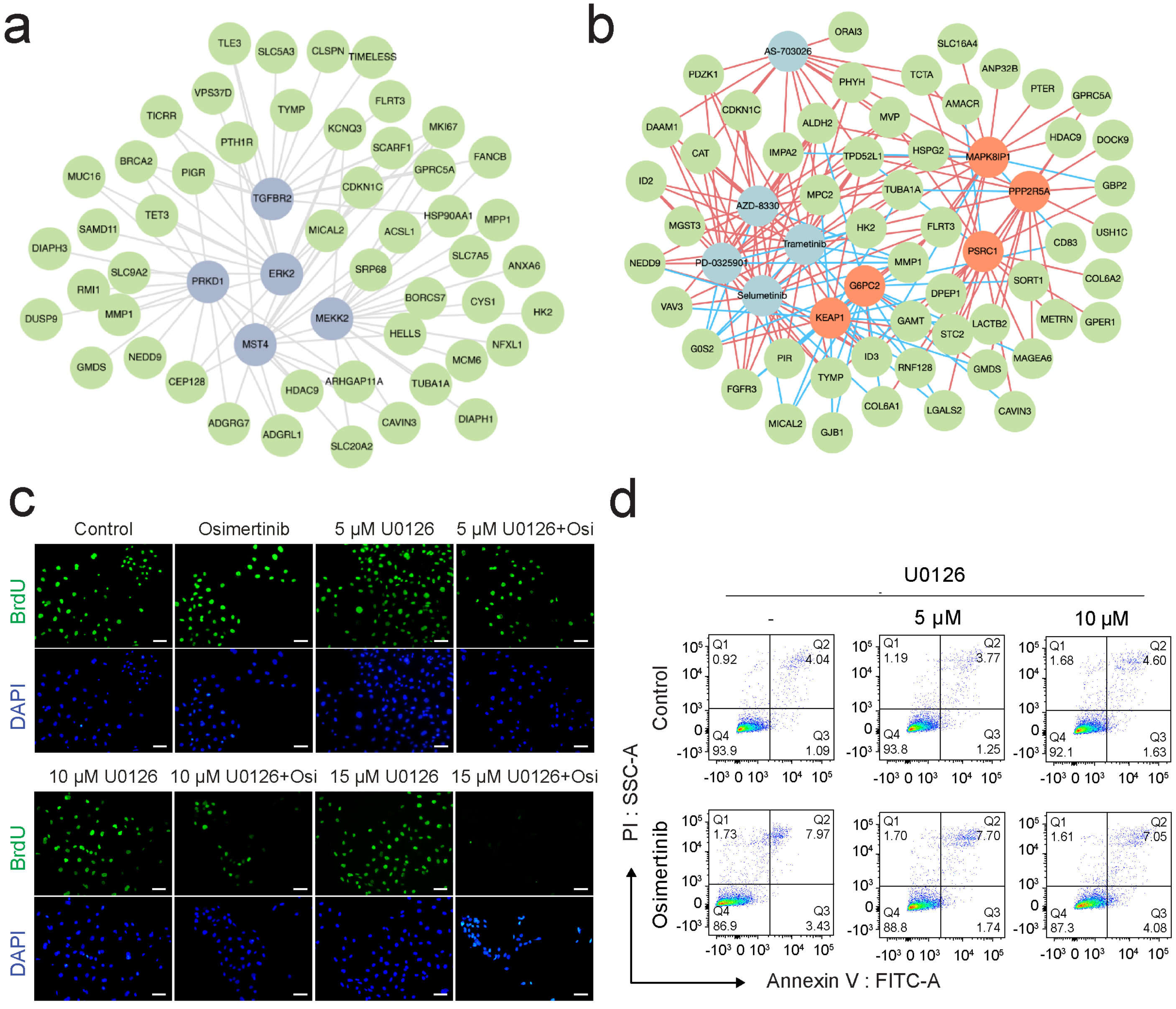
a
b
c
Control
Osimertinib
5 μM U0126
5 μM U0126+Osi
BrdU
DAPI
10 μM U0126
10 μM U0126+Osi
15 μM U0126
15 μM U0126+Osi
d
U0126
-
5 μM
10 μM
Control
Osimertinib
PI : SSC-A
Annexin V : FITC-A

Supplementary Figure 11. The gene expression program is associated with MEK/ERK signaling axis. a,b Network diagrams from EnrichR-KG analysis (https://maayanlab.cloud/enrichr-kg), illustrating enriched kinome substrates (**a**) and drug-gene interactions (**b**). **c** Representative images from BrdU incorporation assay demonstrating the response of HCC827-OsiR cells to combined treatment with U0126 (MEK inhibitor) and osimertinib (1 μM). DAPI was used as a nuclear counterstain (magnification: 20×, scale bar: 50 μm). **d** Flow cytometry plots from Annexin V/PI apoptosis assay in HCC827-OsiR cells following combinatorial drug treatment.

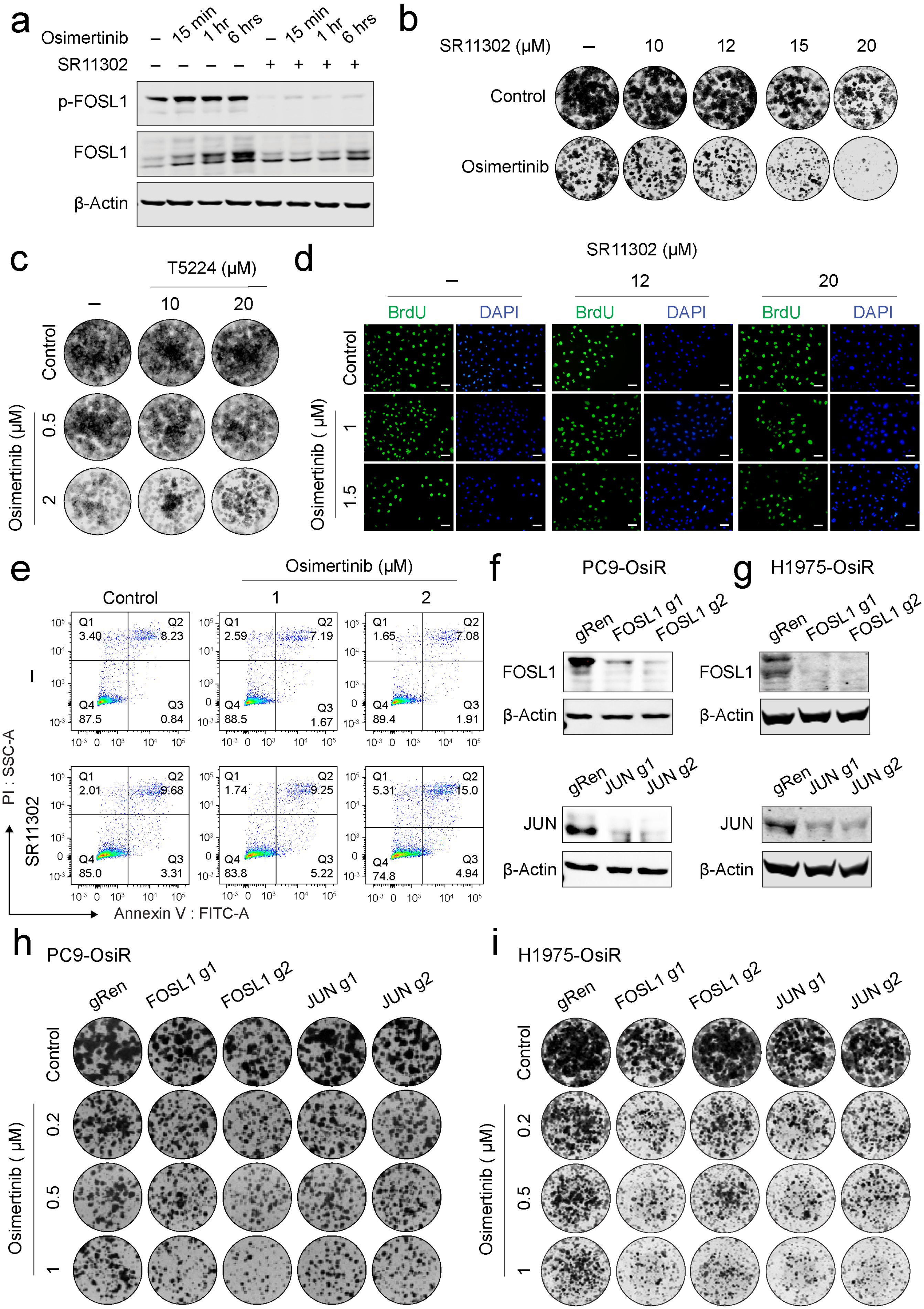

a
Osimertinib – 15 min 1 hr 6 hrs – 15 min 1 hr 6 hrs
SR11302 – – – – + + + +
p-FOSL1
FOSL1
β-Actin
b
SR11302 (μM) – 10 12 15 20
Control
Osimertinib
c
T5224 (μM)
– 10 20
Control
Osimertinib (μM)
0.5
2
d
SR11302 (μM)
– 12 20
BrdU DAPI BrdU DAPI BrdU DAPI
Control
Osimertinib (μM)
1
1.5
e
Osimertinib (μM)
Control 1 2
–
Q1 3.40 Q2 8.23 Q4 87.5 Q3 0.84
Q1 2.59 Q2 7.19 Q4 88.5 Q3 1.67
Q1 1.65 Q2 7.08 Q4 89.4 Q3 1.91
SR11302
Q1 2.01 Q2 9.68 Q4 85.0 Q3 3.31
Q1 1.74 Q2 9.25 Q4 83.8 Q3 5.22
Q1 5.31 Q2 15.0 Q4 74.8 Q3 4.94
PI : SSC-A
Annexin V : FITC-A
f
PC9-OsiR
gRen FOSL1 g1 FOSL1 g2
FOSL1
β-Actin
gRen JUN g1 JUN g2
JUN
β-Actin
g
H1975-OsiR
gRen FOSL1 g1 FOSL1 g2
FOSL1
β-Actin
gRen JUN g1 JUN g2
JUN
β-Actin
h
PC9-OsiR
gRen FOSL1 g1 FOSL1 g2 JUN g1 JUN g2
Control
Osimertinib (μM)
0.2
0.5
1
i
H1975-OsiR
gRen FOSL1 g1 FOSL1 g2 JUN g1 JUN g2
Control
Osimertinib (μM)
0.2
0.5
1

Supplementary Figure 12. Genetic and pharmacological inhibition of AP-1 reinstates sensitivity to osimertinib. **a** Western blot analysis of p-FOSL1 and FOSL1 levels after combinatorial treatment with SR11302 (12 μM) and osimertinib (1 μM) on HCC827-OsiR cells. β-actin was used as a loading control. **b** Representative images from colony formation assay illustrating dose-dependent effects of SR11302 inhibitor (10 to 20 μM) on control or osimertinib osimertinib-treated (1 μM) HCC827-OsiR cells. **c** Dose-dependent effects of T5224 combination with osimertinib in colony formation (n = 3). **d** DNA labeling with BrdU showing the proliferation dynamics following co-treatment with SR11302 and osimertinib. DAPI is used as a nuclear counterstain (magnification 20×, scale bar: 50 μm). **e** Annexin V/PI apoptosis assay in HCC827-OsiR cells following SR11302 (15 μM) and osimertinib combination. Q2 and Q3 show apoptotic cells. **f, g** Western blot of *FOSL1* or *JUN* knockout levels in PC9-OsiR and H1975-OsiR cells. **h, i** Representative images of crystal violet colony formation assays conducted in 12-well culture plates of *FOSL1* or *JUN* knockout PC9-OsiR (**h**) and H1975-OsiR (**i**) cells, in the absence and presence of osimertinib.

Supplementary Tables

Supplementary Table 1. Primary and secondary antibodies used in this study.

Antibody	Vendor	Catalog number	Working dilution
p-EGFR (15A2)	SantaCruz	SC-81488	1:200
p-EGFR (Tyr 1068)	Cell signaling Technology	2234	1:1000
EGF Receptor (D38B1)	Cell signaling Technology	4267T	1:1000
Phospho-Stat3 (Tyr705) (D3A7)	Cell Signaling Technology	9145T	1:2000
Stat3 (79D7)	Cell signaling Technology	4904T	1:2000
p-AKT (S473)	Cell signaling Technology	4060T	1:2000
AKT (C67E7)	Cell signaling Technology	4691T	1:1000
p-p44/42 MAPK (Thr202/Tyr204)	Cell signaling Technology	4370T	1:2000
p44/42 MAPK (137F5)	Cell signaling Technology	4695T	1:1000
Beta Actin (8H10D10)	Cell signaling Technology	3700	1:5000
FRA1 (D80B4)	Cell signaling Technology	5281S	1:1000
Phospho FRA1 (Ser265) (D22B1)	Cell signaling technology	5841	1:1000
JUN	Proteintech	24909-1-AP	1:2000
PARP	Cell signaling Technology	9542	1:1000
Cleaved PARP (Asp214) (D64E10)	Cell signaling Technology	5625	1:1000
VAV3	Proteintech	30291-1-AP	1:8000
Anti-FlagM2 mAb	Sigma	F1804	1:200
Anti-Lamin A+C (4C4)	Abcam	ab190380	1:300 (IF)
α-Tubulin (DMIA) Mouse monoclonal IgG1	SantaCruz	SC-32293	1:200 (IF)
Anti-mouse IgG (H+L) Dylight 680	Cell signaling Technology	5470S	1:10000

Anti-Rabbit IgG (H+L) Dylight 800	Cell signaling Technology	5151P	1:20000
BrdU (Bu20a)	Cell Signalling Technology	5292	1:1000
Donkey anti-mouse Alexa Fluor 488	Abcam	ab150105	1:500
Donkey anti-rabbit Alexa Fluor 568	Abcam	ab175470	1:500
Vimentin (D21H3)	Cell signaling Technology	5741	1:1000<br>1:200 (IF)
E-Cadherin	BD Biosciences	610182	1:1000<br>1:200 (IF)

Supplementary Table 2. Oligonucleotides used in qRT-PCR and Sanger sequencing studies.

Gene name	Forward sequence	Reverse sequence
CDH1	CGAGAGCTACACGTTCACGG	GGCCTTTTGACTGTAATCACACC
VIM	CGTCACCTTCGTGAATACCA	CCAGAGGGAGTGAATCCAGA
SOX2	TTGCTGCCTCTTTAAGACTAGGA	TAAGCCTGGGGCTCAAACT
THBS1	GCTGGAAATGTGGTGCTTGTCC	CTCCATTGTGGTTGAAGCAGGC
CXCL6	GGGAAGCAAGTTTGTCTGGACC	AAACTGCTCCGCTGAAGACTGG
CXCL1	AGCTTGCCTCAATCCTGCATCC	TCCTTCAGGAACAGCCACCAGT
FN1	ACAACACCGAGGTGACTGAGAC	GGACACAACGATGCTTCCTGAG
TWIST2	CATGTCCGCCTCCCACTA	CAATGGCAGCATCATTCAGA

ZEB2	TCCTGTCTGTCTCGCAAAAA	GCCTTGAGTGCTCGATAAGG
ZEB1	GCCAACAGACCAGACAGTGTT	TTTCTTGCCCTTCCTTTCTG
SNAI2	GAAAGGCCTTCAACTGCAAA	TGACATCTGAGTGGGTCTGG
VAV3	GAGTGGAGTCAGCCATCTCTA G	CACGTTGCATAGGAACCACAA GC
VAV3.1	TTACATTTCTTTCAGAACAAG GGACA	TCCACCTGTTTAGGAGTTCTTC G
GAPDH	GGCTGAGAACGGGAAGCTTG TCAT	CAGCCTTCTCCATGGTGGTGAA GA
EGFR_Ex20	AGCCACACTGACGTGCCTCT	CCTTATCTCCCCTCCCCGTA

Supplementary Table 3. Oligonucleotides used for gRNA and shRNA cloning.

Oligo Name	Forward sequence (5' - 3')	Reverse sequence (5' - 3')
gRen	CACCGGGTATAATACACCGCGCTA C	AAACGTAGCGCGGTGTATTATAC CC
RPA3	CACCGGATGAATTGAGCTAGCATG C	AAACGCATGCTAGCTCAATTCAT CC
EGFR g10	CACCGGCTGCCCCGGCCGTCCCGG A	AAACTCCGGGACGGCCGGGGCAG CC
EGFR g60	CACCGTCCTCCAGAGCCCGACTCGC	AAACGCGAGTCGGGCTCTGGAGG AC
FOSL1 g1	CACCGTATTCCTTAGAAGTTCCACC	AAACGGTGGAACTTCTAAGGAAT AC
FOSL1 g2	CACCGTGGTGTTGATGCTTGGCACC	AAACGGTGCCAAGCATCAACACC AC
JUN g1	CACCGGTTGAGGGCATCGTCATAG A	AAACTCTATGACGATGCCCTCAA CC

JUN g2	CACCGCATAAGGTCCGCTCTCGGAC	AAACGTCCGAGAGCGGACCTTATGC
RBBP7 g1	CACCGAGTGTTTGAAGATACTGTGG	AAACCCACAGTATCTTCAAACACTC
RBBP7 g2	CACCGAAGCCACTGAACGGTAAGAC	AAACGTCTTACCGTTCAGTGGCTTC
MTA2 g1	CACCGACGGATTGAGGAGCTCAACA	AAACTGTTGAGCTCCTCAATCCGTC
MTA2 g2	CACCGGACTGCAAATGGAAATGTGG	AAACCCACATTTCCATTTGCAGTCC
FGFR1 g1	CACCGCTGGTCTTAGGCAAACCCCT	AAACAGGGGTTTGCCTAAGACCAGC
FGFR1 g2	CACCGGATCCGGTCAAATAATGCCT	AAACAGGCATTATTTGACCGGATCC
FGF5 g1	CACCGGCCACTGATAGGAACCCTAG	AAACCTAGGGTTCCTATCAGTGGCC
FGF5 g2	CACCGCTGCCGGTCCGGCGCCCCGA	AAACTCGGGGCGCCGGACCGGCAGC
AKT3 g1	CACCGTAAGGTAAATCCACATCTTG	AAACCAAGATGTGGATTTACCTTAC
AKT3 g2	CACCGACAAATTGATAATATAGGAG	AAACCTCCTATATTATCAATTTGTC
SPP1 g1	CACCGAATGGTGAGACTCATCAGAC	AAACGTCTGATGAGTCTCACCATTC
SPP1 g2	CACCGTGGCTTTCGTTGGACTTACT	AAACAGTAAGTCCAACGAAAGCCAC
NLGN1 g1	CACCGTAAAAGGCATAGAAGTACGT	AAACACGTACTTCTATGCCTTTTAC
NLGN1 g2	CACCGTGAAAACGACGTTCCCCTGT	AAACACAGGGGAACGTCGTTTTCAC

AKR1C2 g1	CACCGAGTGGATCTCTGTGCCACAT	AAACATGTGGCACAGAGATCCACTC
AKR1C2 g2	CACCGAGGAATTAGGAGTTATGTTT	AAACAAACATAACTCCTAATTCCTC
shFOSL1	CCGGCCTCAGCTCATCGCAAGAGTACTCGAGTACTCTTGCGATGAGCTGAGGTTTTTG	AATTCAAAAACCTCAGCTCATCGCAAGAGTACTCGAGTACTCTTGCGATGAGCTGAGG
shJUN	CCGGTAGTACTCCTTAAGAACACAACTCGAGTTGTGTTCTTAAGGAGTACTATTTTTG	AATTCAAAAATAGTACTCCTTAAGAACACAACTCGAGTTGTGTTCTTAAGGAGTACTA

Supplementary Table 4. Log2FC and FDR values of top-scoring genes from control vs osimertinib treated HCC827-OsiR CRISPR screen results.

Gene name	Control			Osimertinib		
	LFC	Negative FDR	Positive FDR	LFC	Negative FDR	Positive FDR
NF2	0.78	1	0.002	1.99	1	0.0006
KCTD5	0.74	1	0.002	1.88	1	0.0006
FBXW7	0.52	1	0.023	0.68	1	0.0006
PLEKHA1	0.47	1	0.28	0.68	1	0.04
HNF1B	-2.42	0.0001	1	-2.08	0.0002	1
POLR3H	-2.05	0.0001	1	-1.90	0.0002	1
PAK1IP1	-2.23	0.0001	1	-1.92	0.0002	1
RPP21	-2.57	0.0001	1	-1.78	0.0004	1
