# Supplementary material for: Integrative multi-omics identifies AP-1 transcription factor as a targetable mediator of acquired osimertinib resistance in non-small cell lung cancer
Source: Cell Death Dis. 2025 May 25;16(1):414. doi: 10.1038/s41419-025-07711-z (PMC12104440; doi:10.1038/s41419-025-07711-z)
Supplement: Supplementary file 2 — Uncropped Western Blots [file 41419_2025_7711_MOESM2_ESM.pdf]

Integrative multi-omics identifies AP-1 transcription factor as a targetable mediator of acquired osimertinib resistance in non-small cell lung cancer

Bengisu Dayanc [1,2,*], Sude Eris [1,2,*], Nazife Ege Gulfirat [1,2], Gulden Ozden-Yilmaz [1,2], Ece Cakiroglu [1,2], Ozlem Silan Coskun Deniz [1,2], Gökhan Karakülah [1,2], Serap Erkek-Ozhan [1], Serif Senturk [1,2,#]

Uncropped Western blots images

Figure 1I

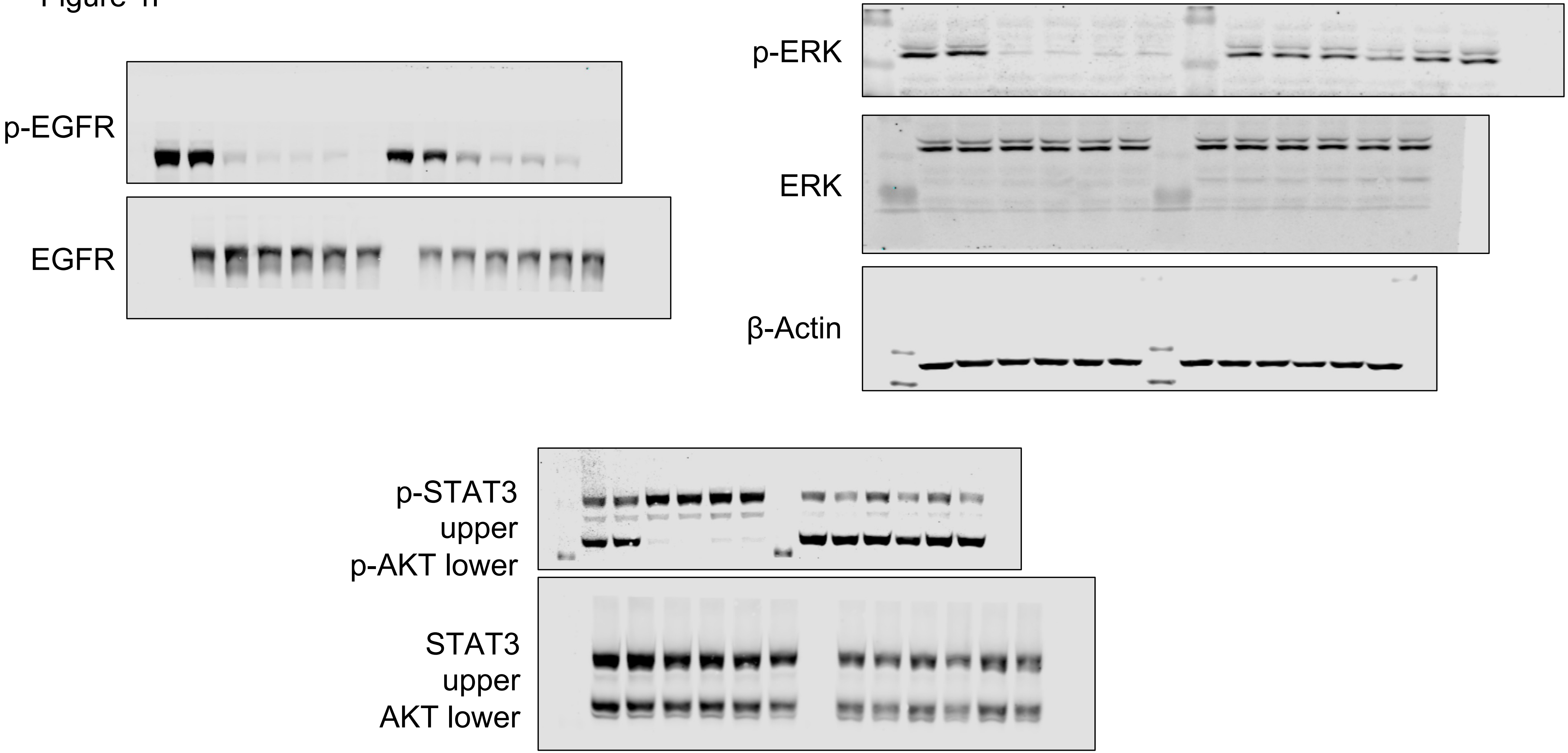
p-EGFR
EGFR
p-ERK
ERK
β-Actin
p-STAT3 upper
p-AKT lower
STAT3 upper
AKT lower

Figure 2o

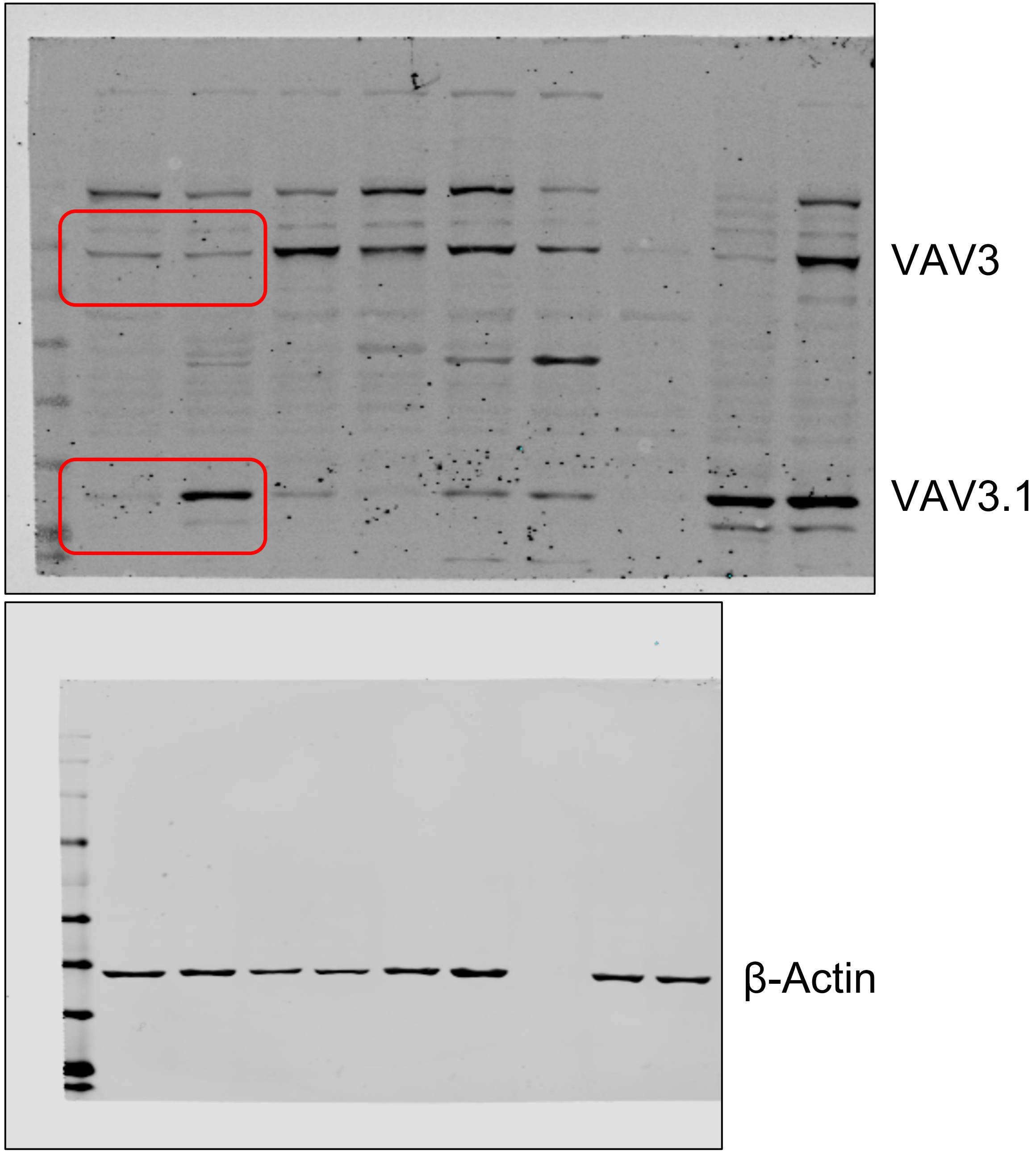

Figure 4b

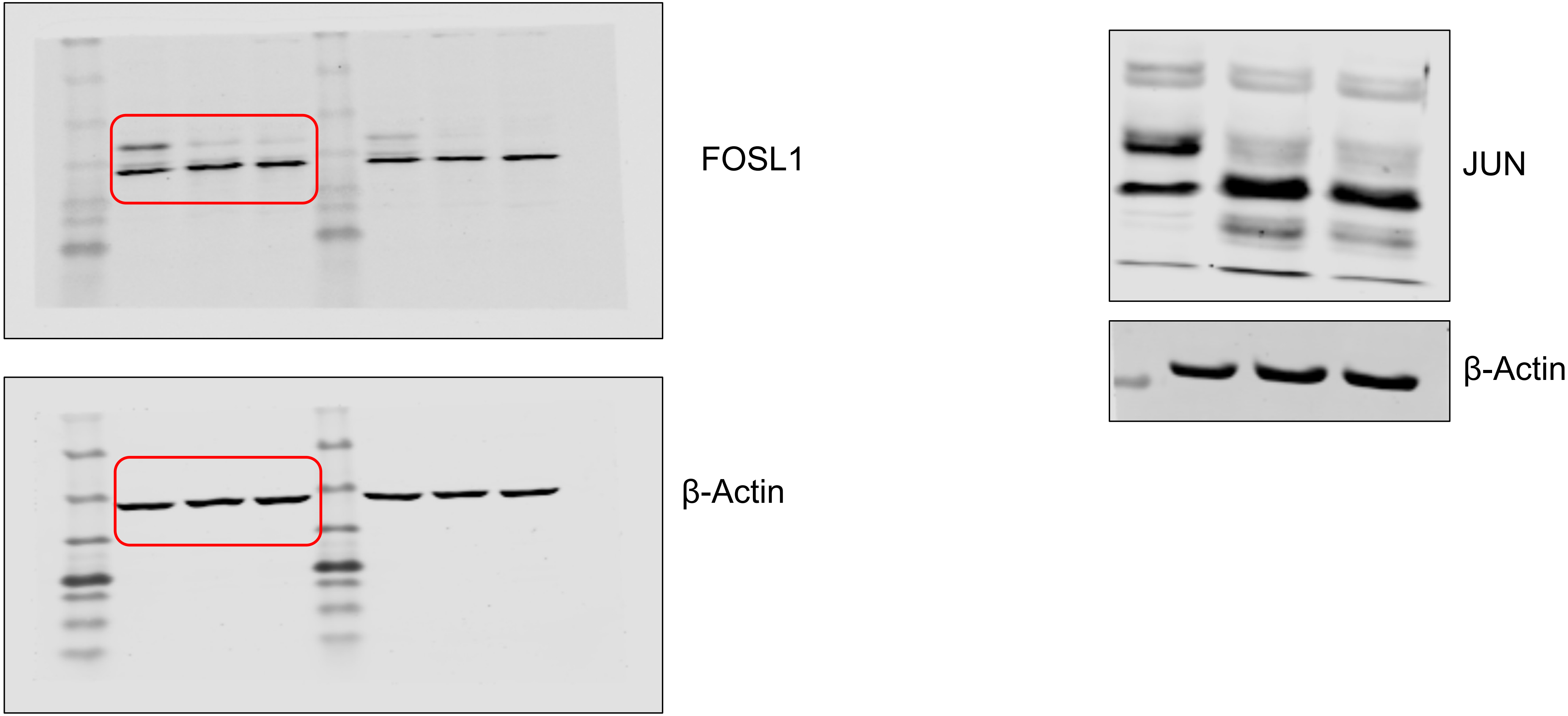

Figure 4c

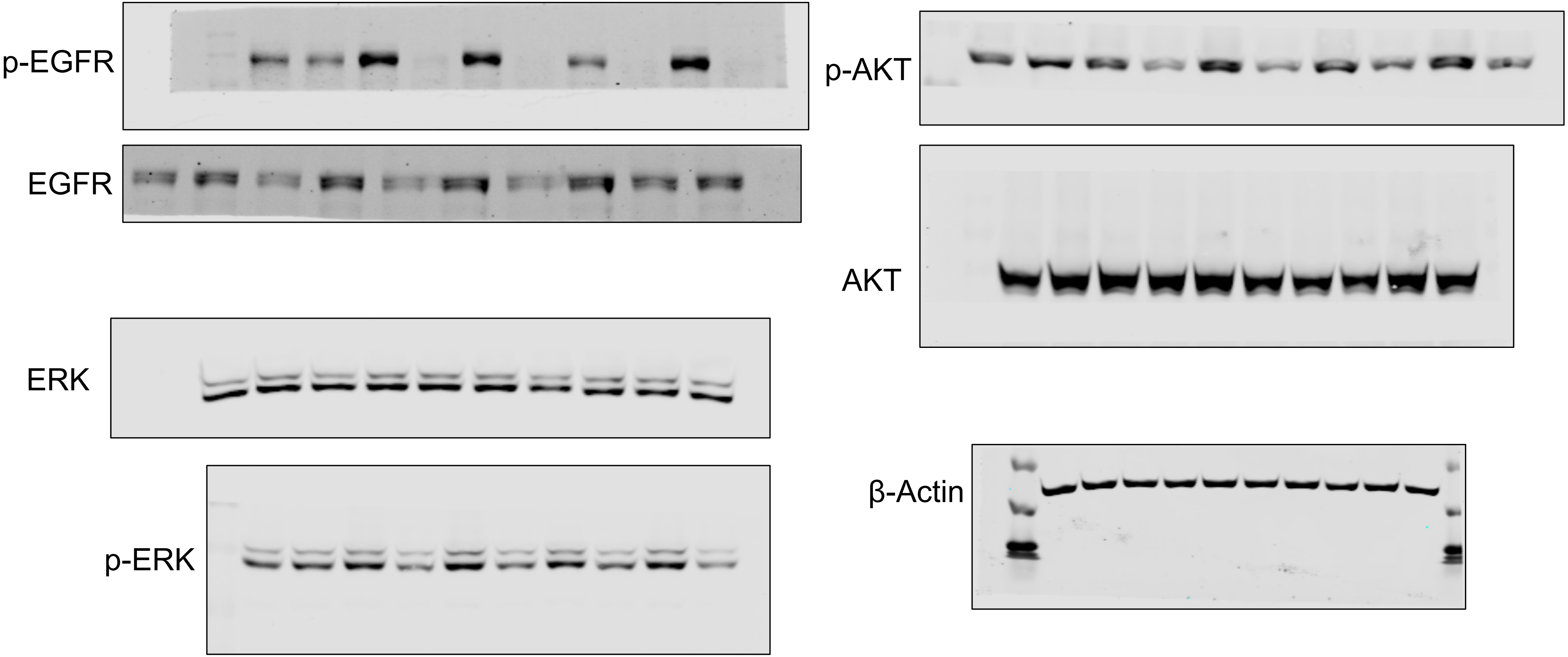

Figure 5

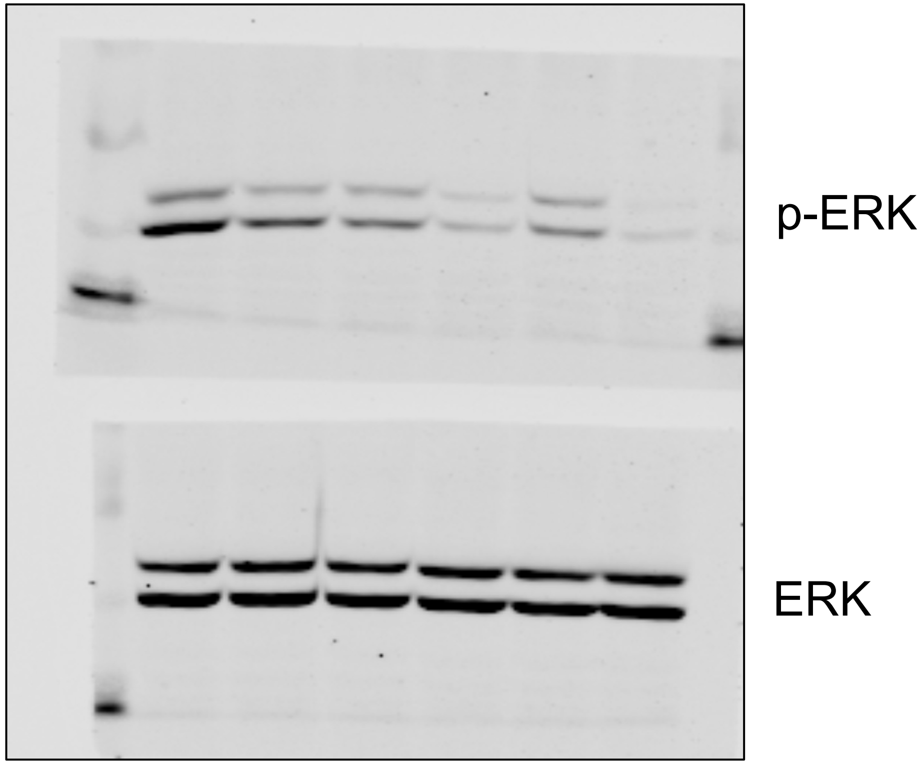

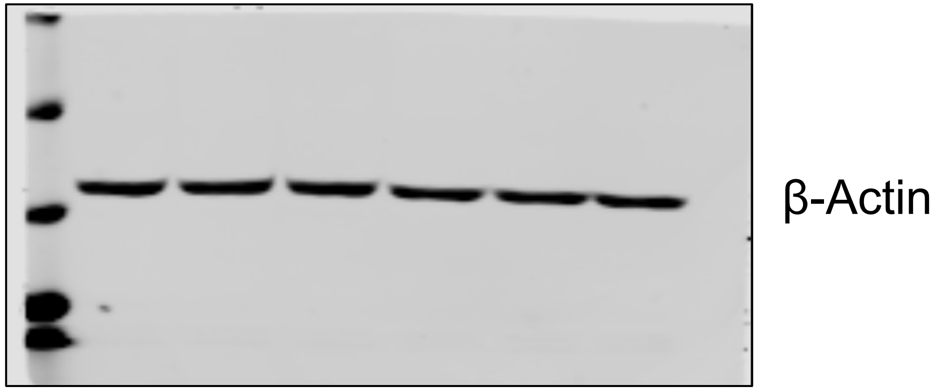

Figure 6a

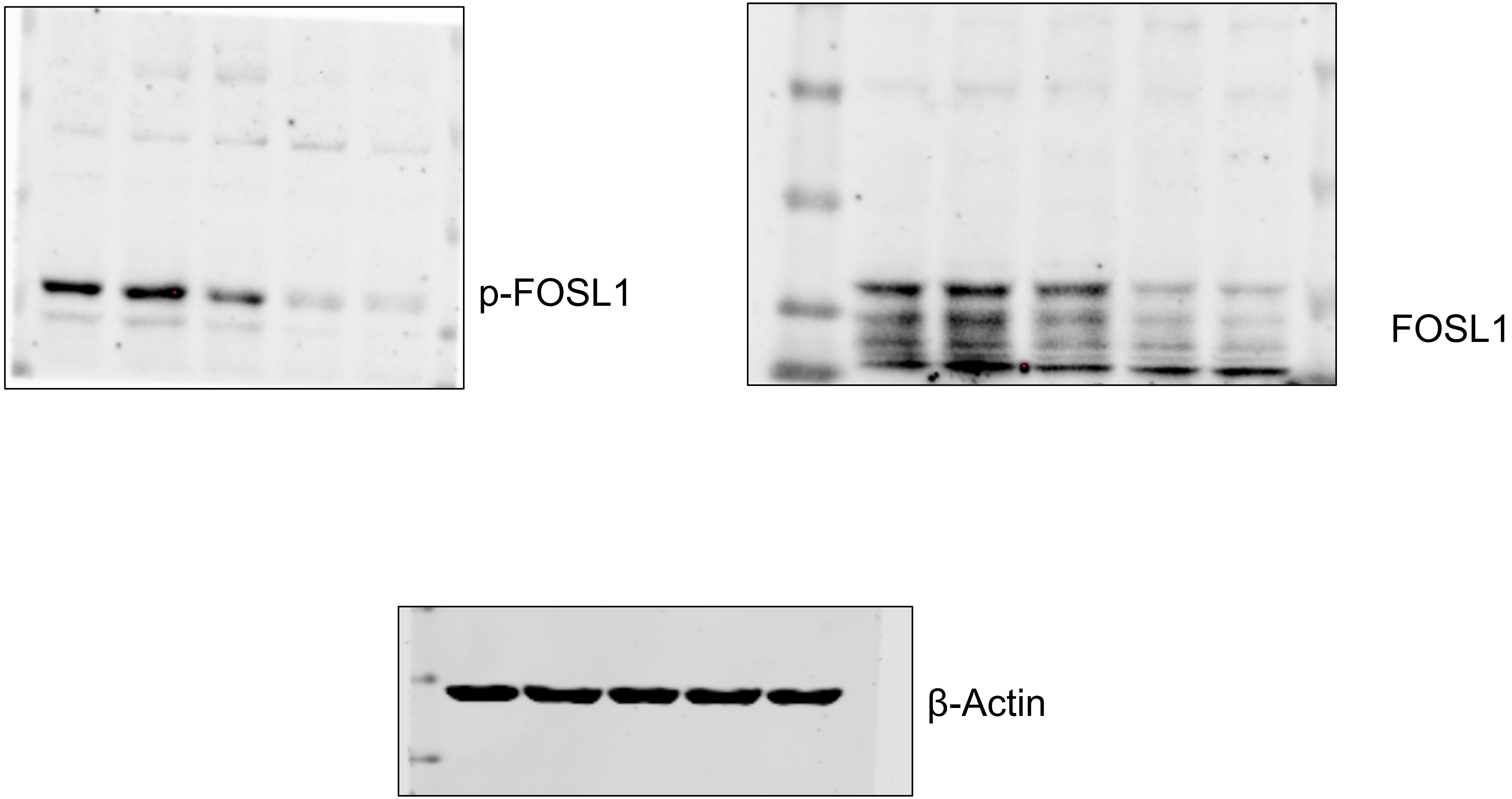

Figure 6b

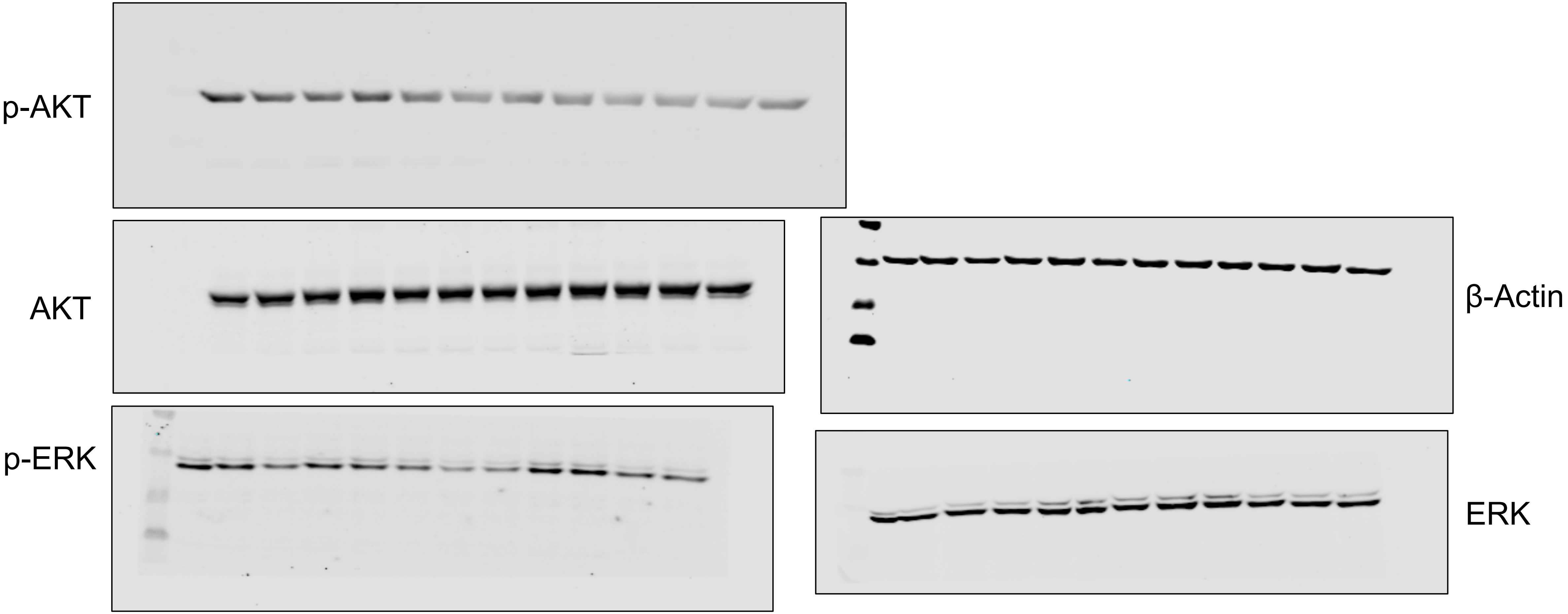

Supp Fig 1d

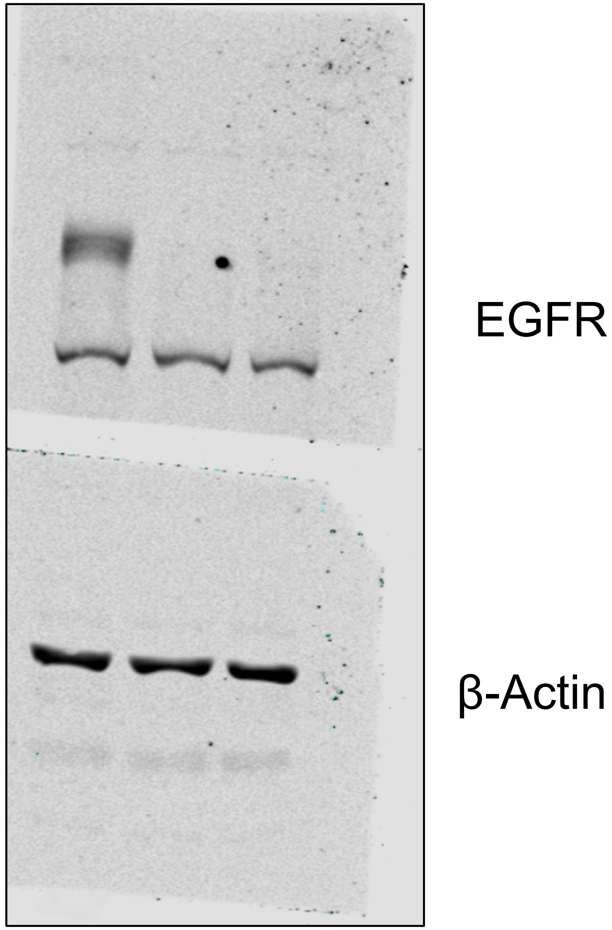

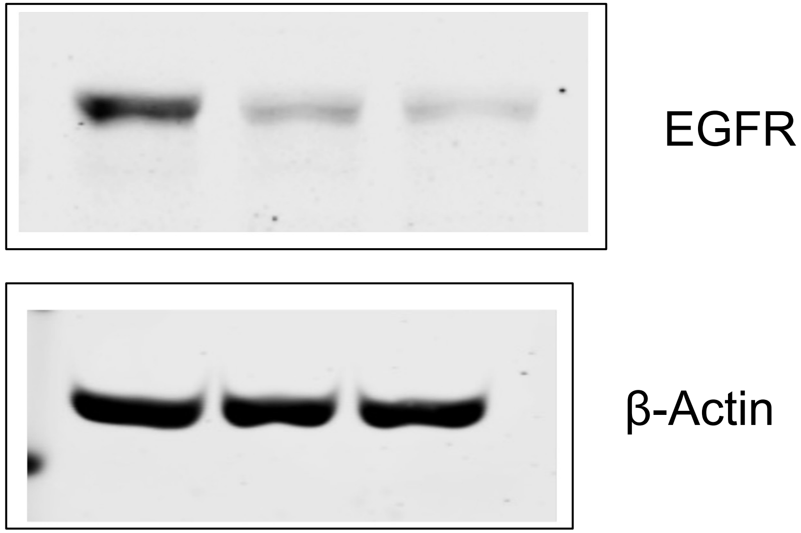

Supp Fig 2e

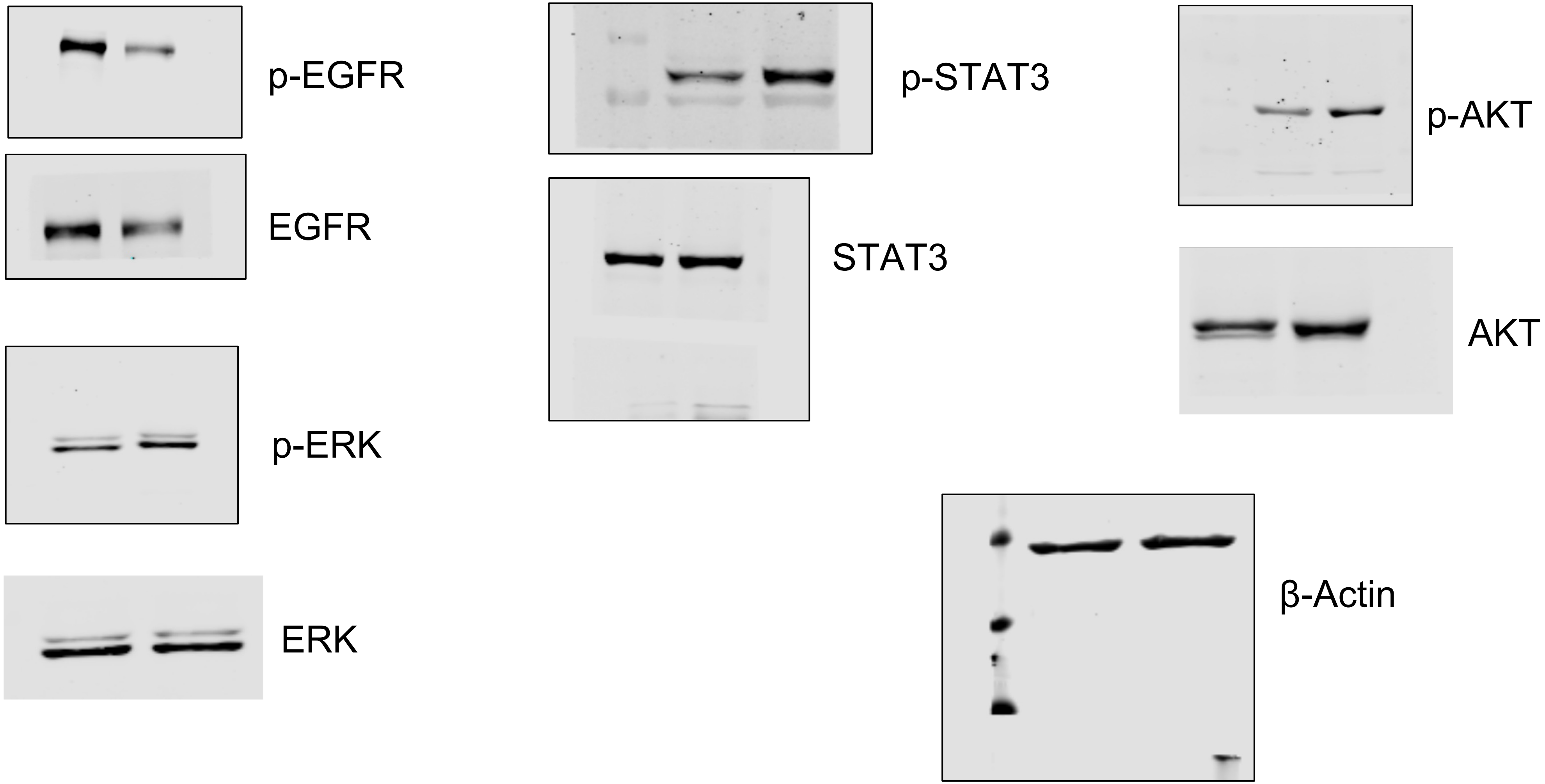

Supp Fig 4c

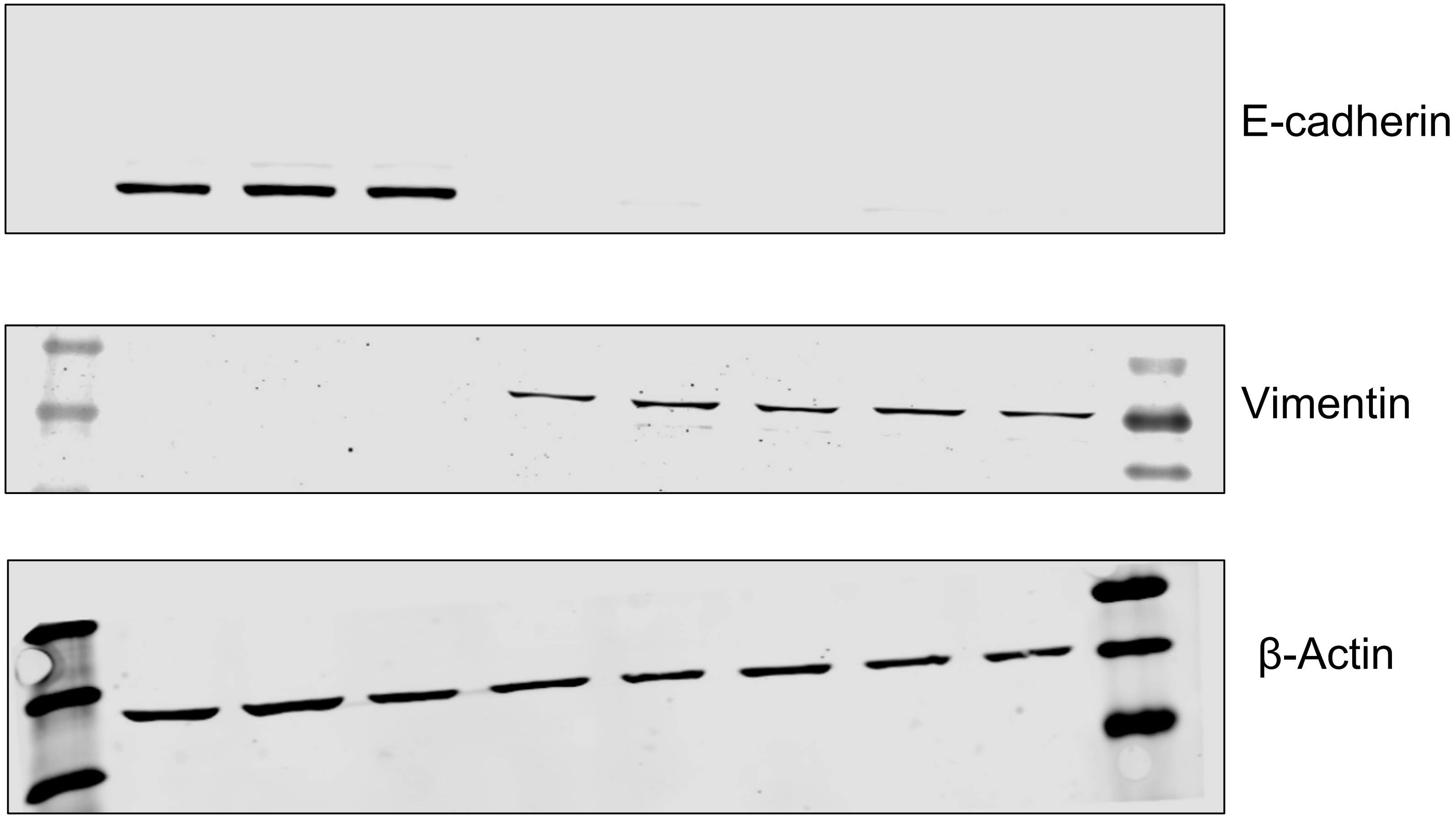

Supp Fig 4f.

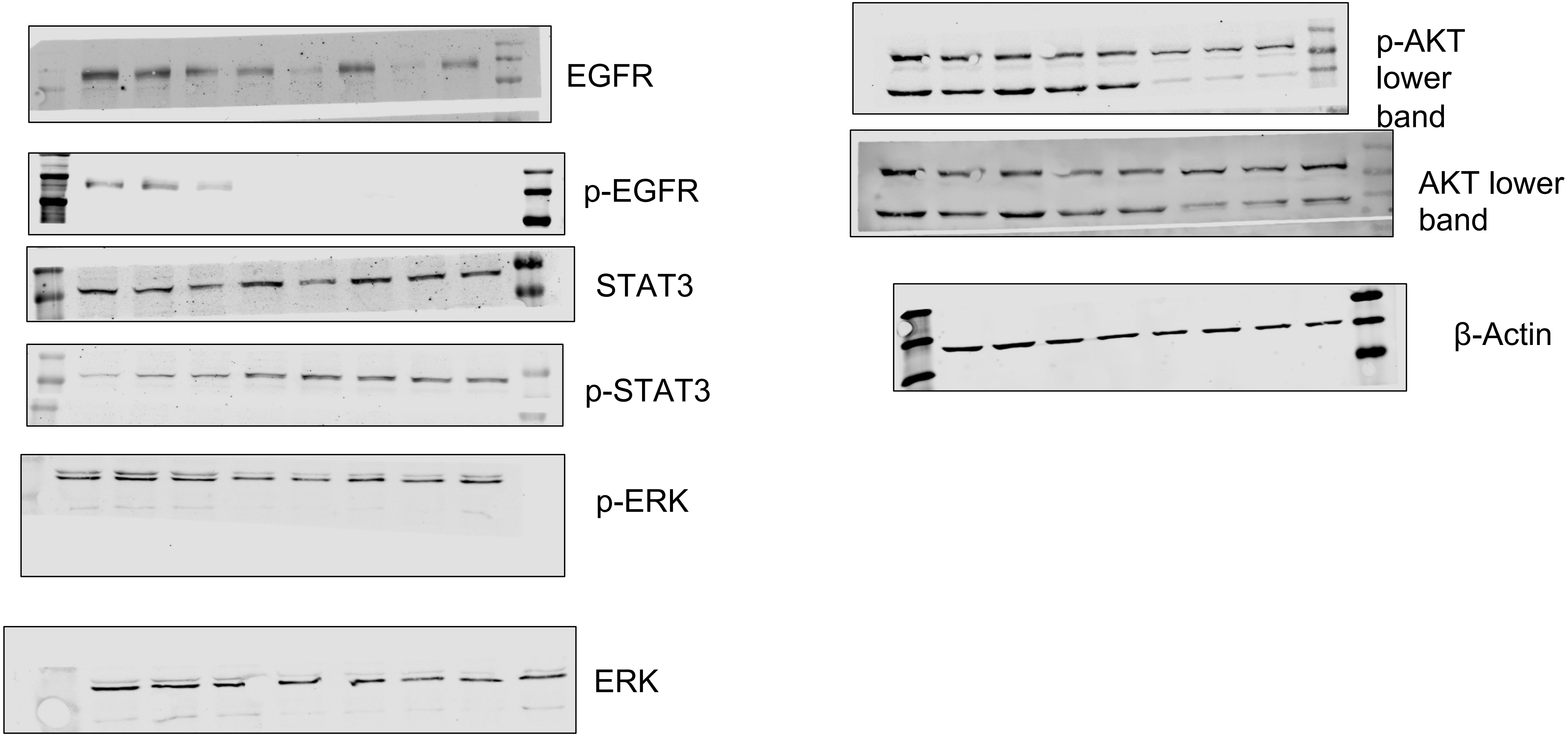
EGFR
p-EGFR
STAT3
p-STAT3
p-ERK
ERK
p-AKT lower band
AKT lower band
β-Actin

Supp Fig 7c

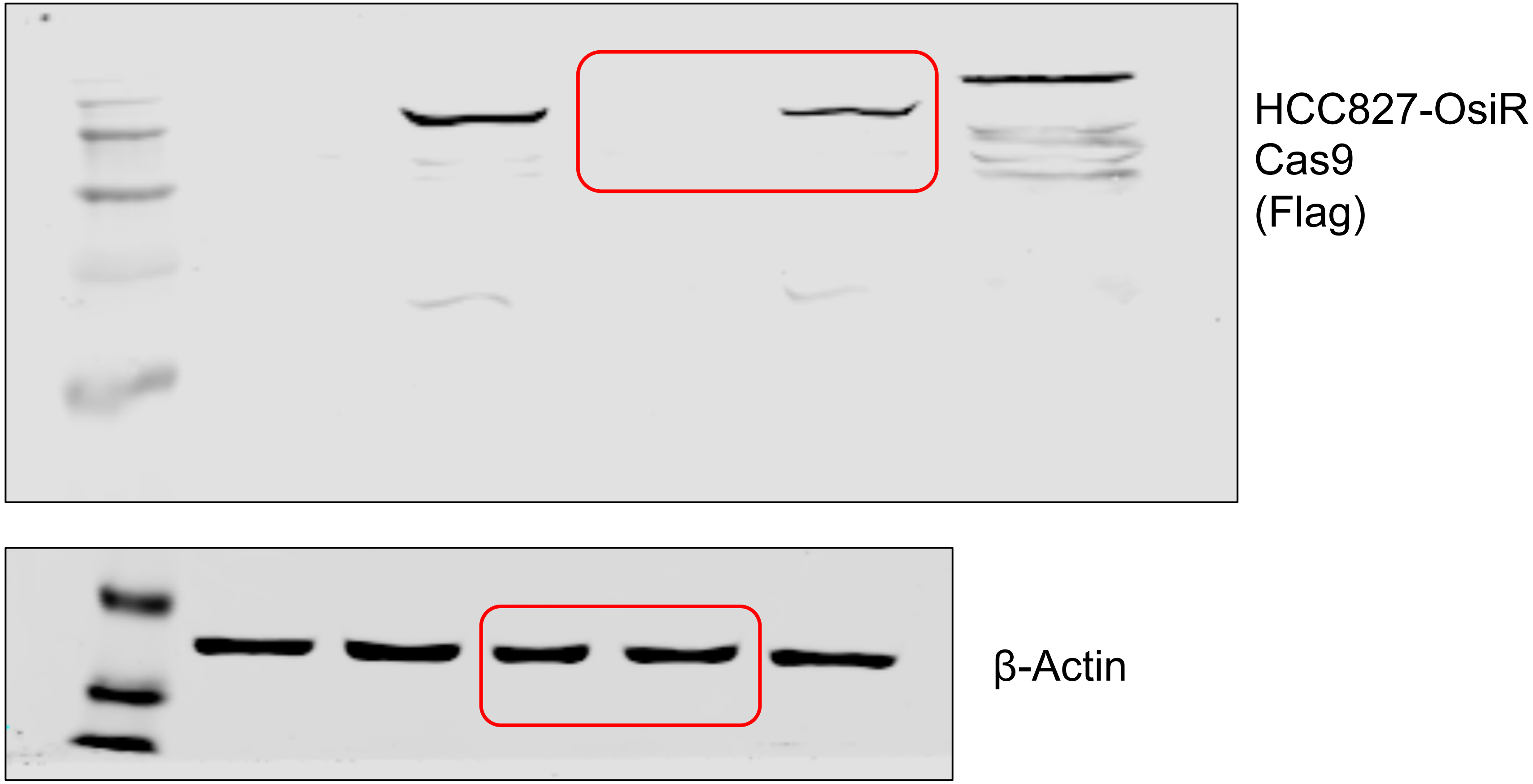

Supp Fig 7k

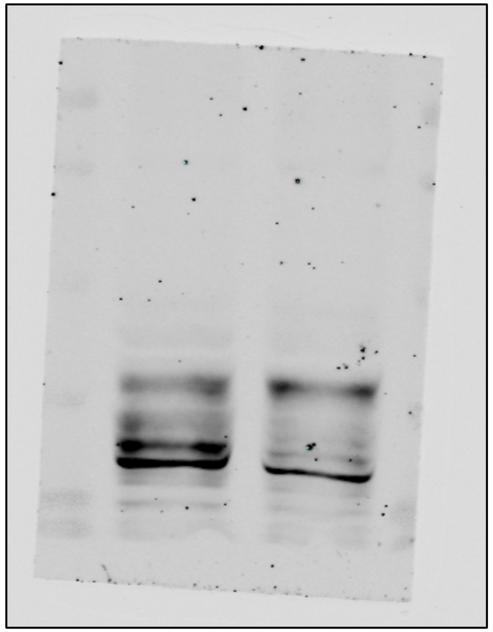

FOSL1

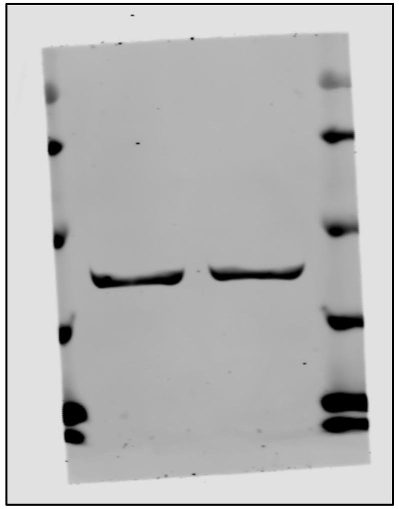

β-Actin

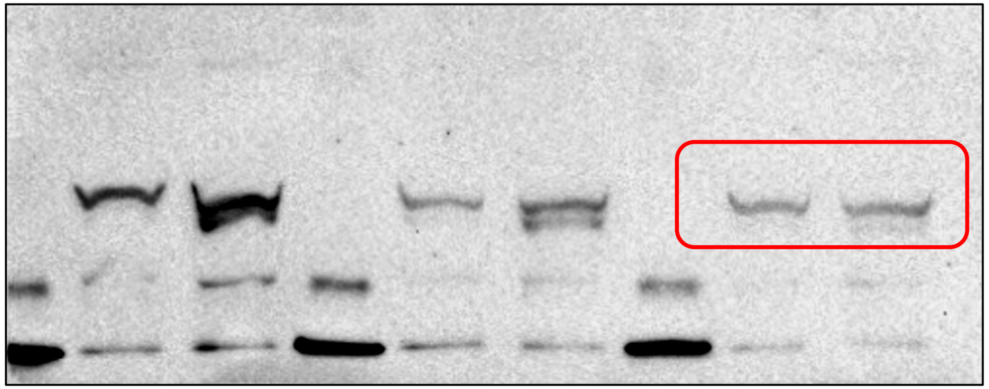

JUN

Supp Fig 9a

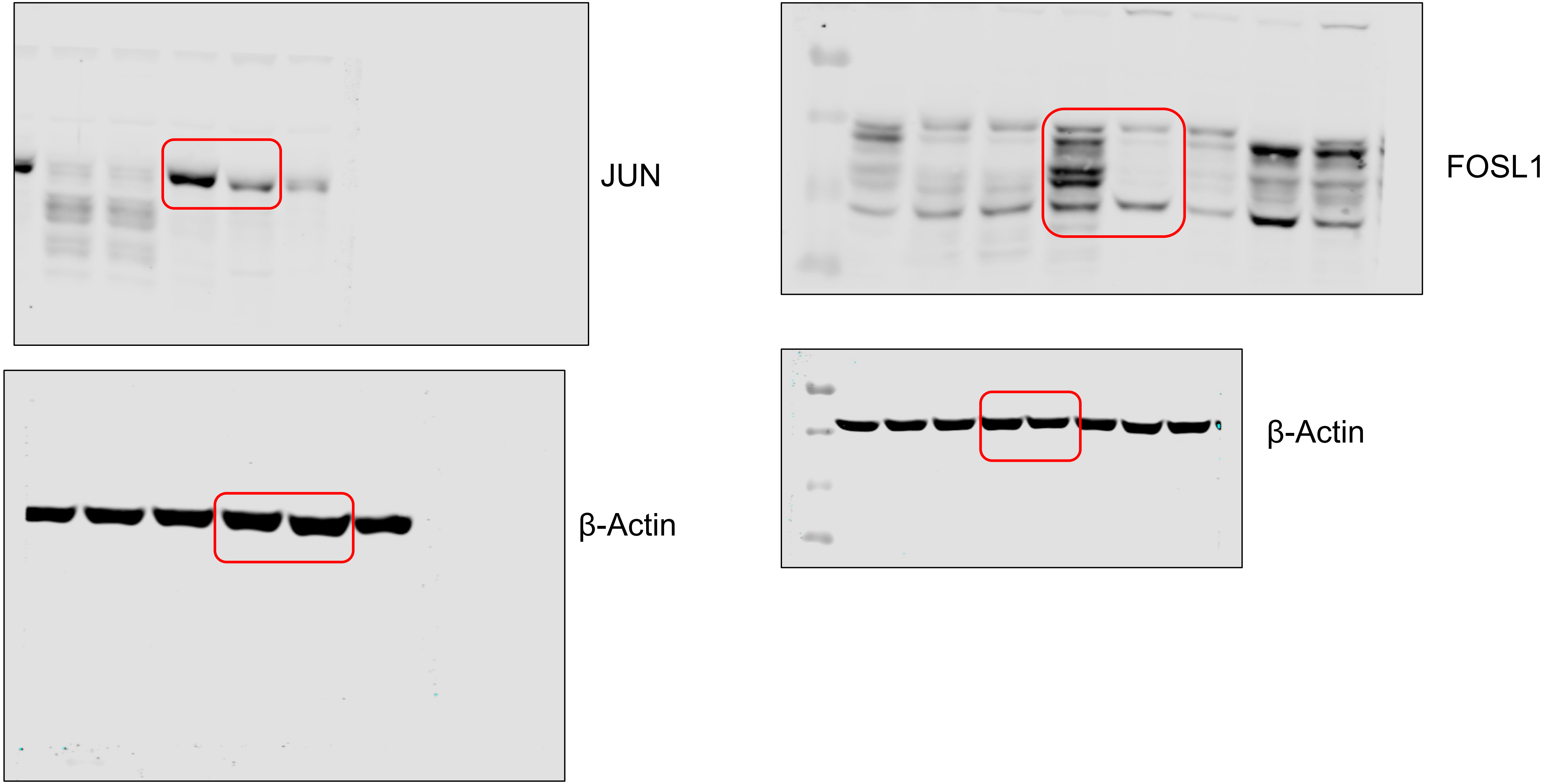

Supp Fig 12a

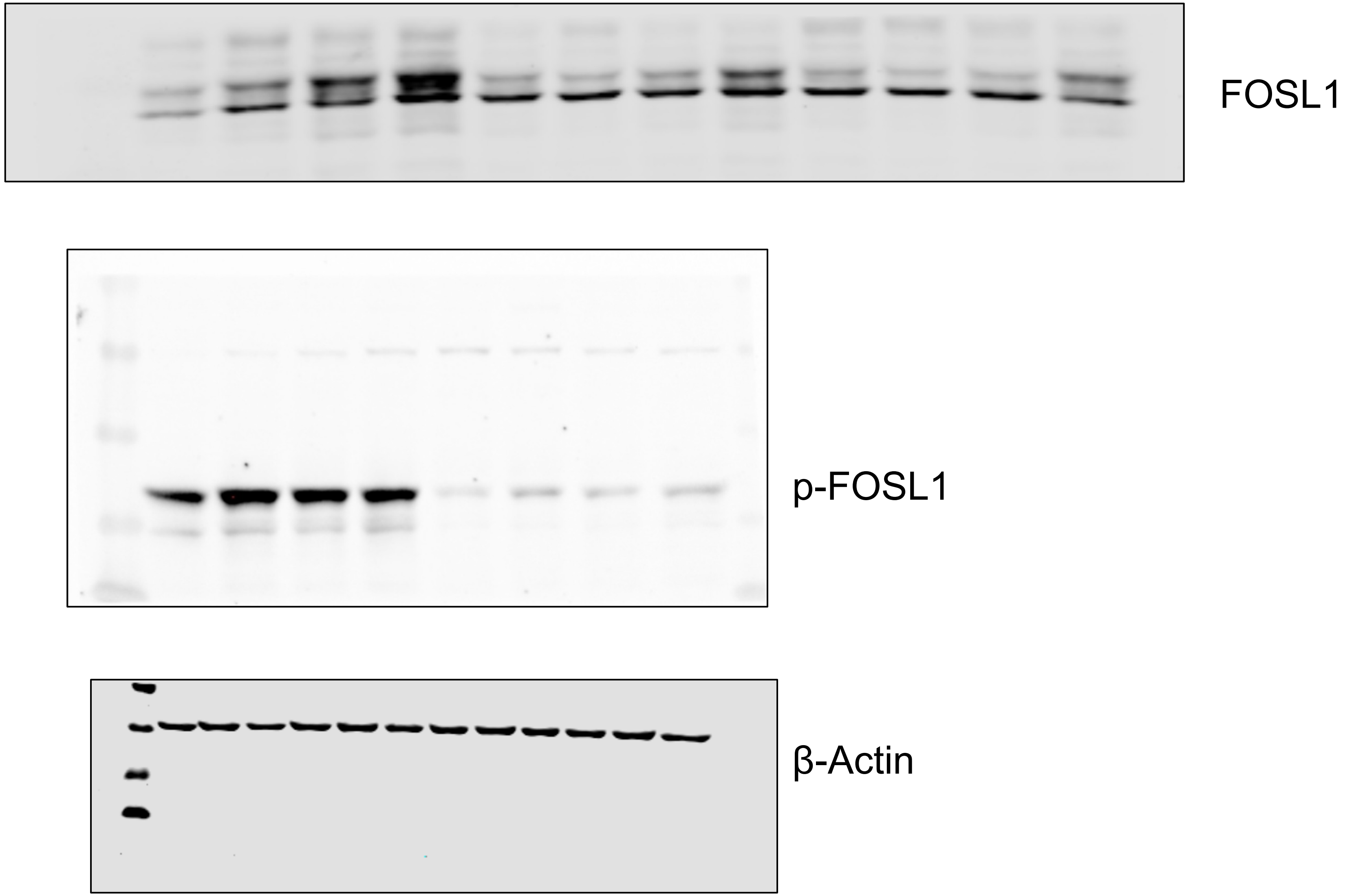

Supp Fig 12f

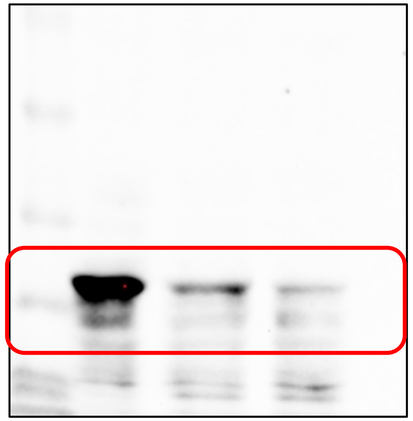

FOSL1

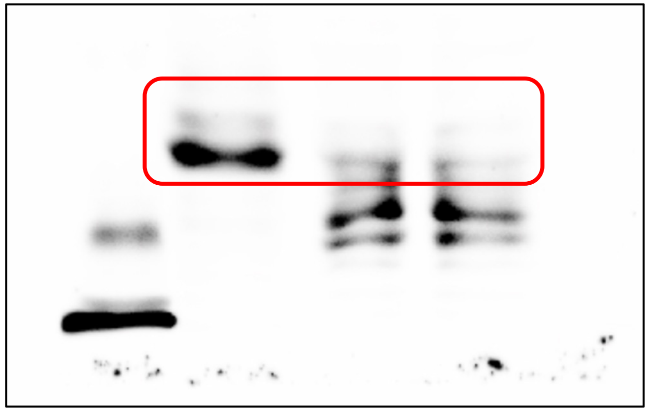

JUN

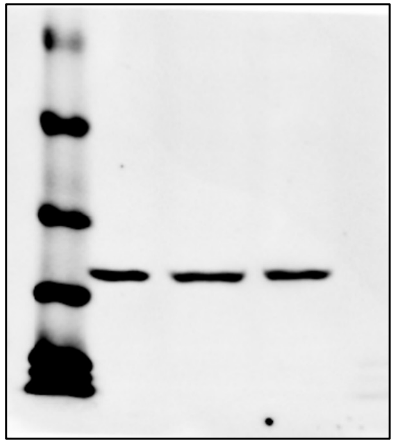

β-Actin

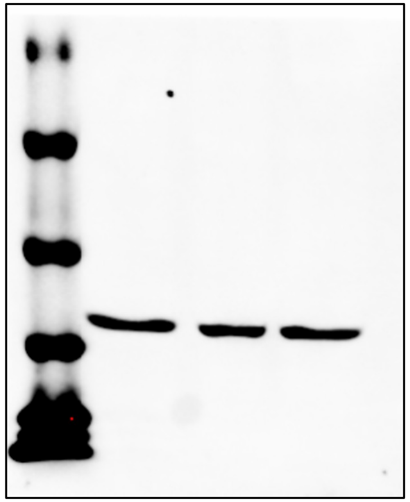

β-Actin

Supp Fig 12g

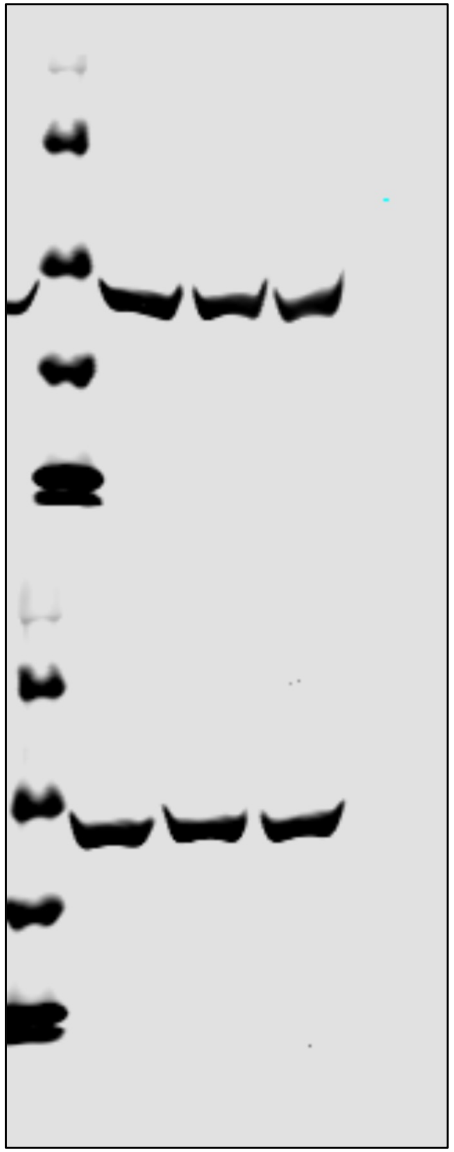

JUN blot's β-Actin

FOSL1 blot's β-Actin

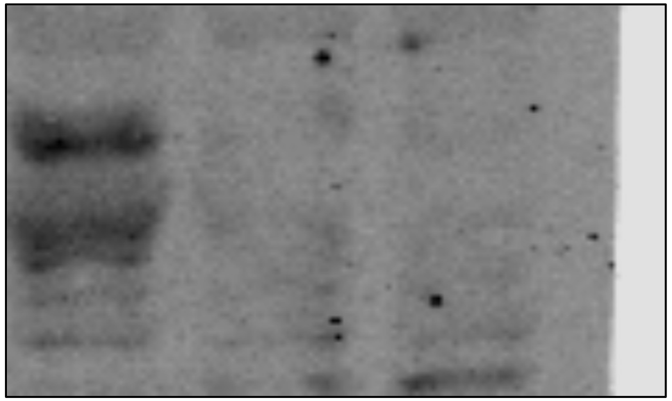

FOSL1

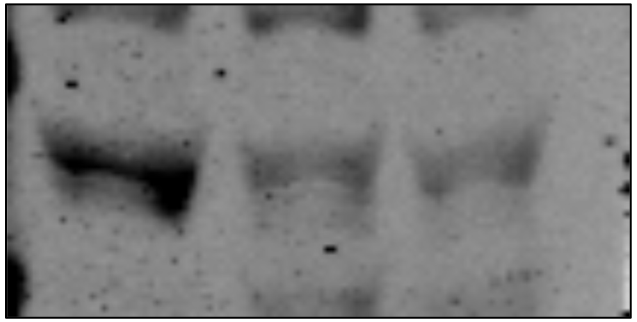

JUN
